# Engineering *g*‐C_3_N_4_/CsPbCl_3_ Heterojunctions for Selective Photocatalytic Oxidation of Benzylic Alcohols

**DOI:** 10.1002/cssc.71146

**Published:** 2026-10-14

**Authors:** Camilla Callegari, Emanuele Bosetti, Giulia Giovilli, Davide Ravelli, Lorenzo Malavasi

**Affiliations:** ^1^ PhotoGreen Lab Department of Chemistry University of Pavia Pavia Italy; ^2^ Energy and Materials Chemistry Group Department of Chemistry and INSTM University of Pavia Pavia Italy

**Keywords:** carbonyls, heterojunction, oxidation, perovskites, photocatalysis

## Abstract

The selective oxidation of benzylic alcohols to carbonyl compounds is a fundamental transformation in the synthesis of fine chemicals and pharmaceuticals, yet conventional methods often rely on stoichiometric oxidants and generate significant waste. Herein, we report a sustainable heterogeneous photocatalytic system based on engineered *g*‐C_3_N_4_/CsPbCl_3_ heterojunctions for the aerobic oxidation of benzylic alcohols under visible‐light irradiation. A series of heterojunction photocatalysts with different compositions was prepared and evaluated, identifying the material containing 10%_W/W_ CsPbCl_3_ as the most efficient catalyst. Under mild conditions and using molecular oxygen as the sole oxidant, a broad range of primary and secondary benzylic alcohols was selectively converted into the corresponding aldehydes and ketones in good to excellent yields. The methodology proved scalable to gram‐scale synthesis and the photocatalyst could be readily recovered and reused over multiple cycles while preserving both catalytic activity and structural integrity. Mechanistic studies indicate that substrate activation proceeds through distinct pathways depending on its redox properties, involving either direct single‐electron oxidation or oxidation mediated by superoxide radical anion. This work demonstrates the potential of metal halide perovskite/carbon nitride heterojunctions as robust and recyclable photocatalysts for sustainable aerobic oxidation processes and green organic synthesis.

## Graphical abstract

A recyclable *g*‐C_3_N_4_/CsPbCl_3_ heterojunction photocatalyst enables the selective aerobic oxidation of primary and secondary benzylic alcohols under visible light irradiation using O_2_ as the terminal oxidant. Efficient charge separation at the semiconductor interface drives the radical‐mediated formation of aldehydes and ketones under mild conditions with good substrate scope and catalyst reusability.

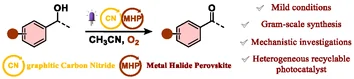

## 1 Introduction

The oxidation of benzylic alcohols to the corresponding aldehydes or ketones is one of the most important transformations in synthetic organic chemistry [1, 2, 3, 4, 5, 6], owing to the broad utility of aromatic carbonyl compounds in the pharmaceutical and fine chemical industries [7]. Conventional oxidation methods typically rely on stoichiometric amounts of toxic transition‐metal‐based reagents, generating significant environmental and sustainability concerns. In response, considerable efforts have been devoted to the development of greener alternatives [8, 9], including the adoption of metal‐free oxidants [10, 11], electrochemical methodologies [12, 13], and catalytic aerobic oxidation processes [14] that employ molecular oxygen as an environmentally benign oxidant [15, 16]. Among these approaches, visible‐light photoredox catalysis has recently emerged as a promising strategy, enabling the oxidation of benzylic alcohols under mild and environmental‐friendly conditions through the use of photoexcited catalysts [3, 17, 18, 19, 20, 21].

In parallel, the development and exploitation of innovative heterogeneous photocatalysts are increasingly recognized as a sustainable approach, with broad applications in renewable energy generation, environmental remediation, and green synthetic methodologies [22]. In the field of organic synthesis, such strategy has gained significant interest because heterogeneous catalysts can be readily separated from the crude reaction mixtures containing the product of interest. This feature allows streamlining downstream purification procedures and enables catalyst reuse, enhancing both practicality and sustainability [23]. Along this line, metal halide perovskites (MHPs) have recently emerged as promising heterogeneous photocatalysts owing to their outstanding optoelectronic properties, including strong light absorption, efficient charge generation, and highly tunable electronic structures [24]. Beyond their established role in photovoltaics, MHPs have demonstrated significant potential in organic synthesis, enabling a variety of photoredox transformations such as selective oxidations, reductions, and C─C bond‐forming reactions under mild conditions [25]. A unique feature of MHPs is the possibility of tailoring their bandgap and band edge positions through compositional engineering. In particular, the nature of the halide plays a crucial role in determining the redox properties of the material. Moving from iodide‐ to bromide‐ and chloride‐based perovskites generally results in a progressive widening of the bandgap and a shift of the valence and conduction bands, thereby modifying the driving force available for oxidation and reduction processes [26, 27]. Such flexibility, combined with the ease of synthesis and compositional versatility of MHPs, makes them attractive platforms for the development of selective heterogeneous catalysts for organic transformations.

On the other hand, the coupling of MHPs with a second semiconductor to form a heterojunction promotes charge separation and suppresses electron–hole recombination. Among the various partners explored in the current literature, polymeric carbon nitride (C_3_N_4_) has emerged as a particularly attractive candidate owing to its convenient band alignment, chemical stability, low toxicity, and tunable structure. This combines with a facile synthesis from inexpensive, widely available precursors and a proven ability to facilitate interfacial charge transfer [28, 29, 30]. In addition, C_3_N_4_‐based materials exhibit excellent activity in the aerobic oxidation of boronic acids and the selective oxidation of alcohols to ketones and aldehydes or carboxylic acids, underscoring their versatility in synthetic transformations [31]. Both MHPs and MHP‐based heterojunction systems have demonstrated remarkable performance in diverse applications, such as hydrogen evolution, nitrogen fixation, CO_2_ reduction, and pollutant degradation [32]. Beyond these well‐established areas, growing interest has recently emerged in exploiting MHP photocatalysts for a wide variety of organic synthetic transformations [28, 33].

In order to show the potential of MHP‐based heterojunctions in the oxidation of benzylic alcohols, we devised a heterogeneous photocatalytic system based on CsPbCl_3_ and C_3_N_4_ coupled to form a heterojunction. CsPbCl_3_ has been selected as MHP due to its favorable band edge position to run oxidation reactions [27]. This novel *g*‐C_3_N_4_/CsPbCl_3_ heterojunction system is capable of behaving as a sustainable heterogeneous photocatalyst for the aerobic oxidation of a broad range of benzylic alcohols under visible‐light irradiation.

## 2 Results and Discussion

The heterogeneous photocatalysts (**PC1‐6**) employed in this work have been prepared as described in the Supporting Information (see Section 2.2). Among them, four *g*‐C_3_N_4_/CsPbCl_3_ heterojunctions were synthesized with different weight ratios (%_W/W_) of the two components, comprising 10, 30, 50, and 90%_W/W_ expressed as the relative amount of the perovskite component (**PC2‐5**, respectively). At the same time, the pure semiconductors, namely, nanoexfoliated *g*‐C_3_N_4_ (**PC1**) and CsPbCl_3_ (**PC6**), have been prepared, and their performance evaluated as well for comparison purposes (see Figure 1a). Powder X‐ray diffraction (XRD) patterns of *g*‐C_3_N_4_ and CsPbCl_3_ are reported in Figure 1b (against the calculated cubic structure [34], see Section S1.1), while Figure 1c reports the XRD patterns of the **PC2‐5** heterojunctions. The perovskite crystal structure is preserved in all heterojunction samples, as confirmed by the XRD patterns reported in Figure 1c. As expected, the diffraction profiles are dominated by the intense reflections of CsPbCl_3_, while the characteristic *g*‐C_3_N_4_ diffraction peak at approximately 27° is clearly visible in samples **PC2‐4**. UV–vis absorption spectra of the reference materials (CsPbCl_3_ and *g*‐C_3_N_4_) and the heterojunctions are reported in Figures S2 and S3, respectively (see Supporting Information). Tauc plot analysis revealed optical bandgaps of approximately 2.8 eV for CsPbCl_3_ and 2.7 eV for *g*‐C_3_N_4_, in good agreement with literature values [35, 36]. The absorption spectra of the **PC2‐5** heterojunctions (Figure S3) show a progressive extension of the absorption range with increasing C_3_N_4_ content, accompanied by the emergence of an absorption tail that becomes more pronounced at high C_3_N_4_ loadings.

**FIGURE 1:**
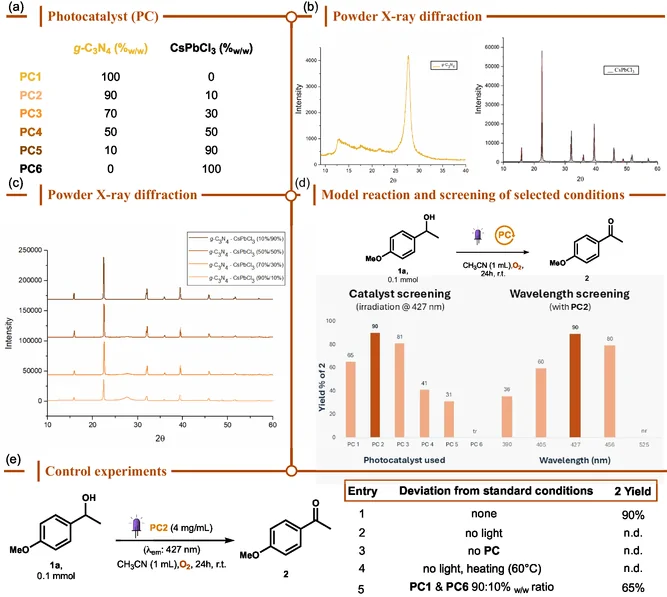
(a) Photocatalysts (**PC**) employed in the present work. (b) Powder X‐ray diffraction (XRD) patterns of *g*‐C_3_N_4_ and CsPbCl_3_ against the calculated cubic structure (PDF card 75‐0408). (c) XRD patterns of the **PC2‐5** heterojunctions. (d) Model reaction involving the photocatalytic oxidation of 1‐(4‐methoxyphenyl)ethan‐1‐ol (**1a**) at the benzylic position under aerobic conditions and screening of selected reaction conditions. (e) Control experiments for the model reaction; GC‐FID yields are reported (see general procedure **GP‐*a*** in Section S3.1 for further details). n.d., not detected; tr., traces.

We next evaluated the performance of the prepared heterojunctions as heterogeneous photocatalysts in the model oxidation at the benzylic position of 1‐(4‐methoxyphenyl)ethan‐1‐ol **1a** to 1‐(4‐methoxyphenyl)ethan‐1‐one **2** under aerobic conditions (Figure 1d). Systematic screening of several reaction parameters enabled maximize the formation of the desired product **2** (see Section S1.3 for full details). Under optimized conditions, irradiation of **1a** (0.1 M in MeCN; a solvent screening is reported in Table S3) for 24 h in the presence of the selected photocatalyst (**PC**) under an O_2_ atmosphere (bubbling O_2_ for ~1 min) afforded product **2** in up to 90% yield, as determined by G,as chromatography ‍‐‍ flame ionization detector (GC‐FID) analysis. Among the materials examined, **PC2** exhibited the best performance, delivering the highest yield of **2** upon irradiation with violet light (LED lamp with emission centered at 427 nm), which also proved to be the most suitable irradiation source (Figure 1d). Control experiments conducted in the dark (irradiation omitted), in the absence of **PC**, or under purely thermal conditions (reaction mixture heated to 60 °C without irradiation) resulted in no formation of **2**, confirming the photocatalytic nature of the transformation. Moreover, the use of a physical mixture of *g*‐C_3_N_4_ and CsPbCl_3_ as the photocatalyst led to diminished activity (Figure 1e).

With the optimized conditions in hand, we next investigated the scope and versatility of the benzylic oxidation under preparative conditions (0.5  mmol scale on 5 mL reaction mixture, Table 1). The complete list of starting materials, together with the observed scope limitations, is provided in Section 1.2 of the Supporting Information. We initially explored the substrate scope for the benzylic oxidation of secondary alcohols, starting with *para*‐substituted derivatives **1a–e**, which afforded the corresponding products **2–5** in variable yields, depending on the nature of the substituent attached to the aromatic ring. Among these substrates, only the *para*‐fluoro derivative **1e** proved unreactive under the optimized conditions (no product **6** formed). We then turned our attention to *meta*‐substituted substrates **1f–i**, which underwent smooth conversion to aromatic ketone products **7–10** in modest to excellent yields. The same substituents were then examined at the *ortho*‐position as well (substrates **1j–m**). In this case, a general decrease in reactivity was observed: the *ortho*‐methoxy‐substituted alcohol (**1j**) afforded the desired product **11** in reduced yield (<50% of isolated product), while the *ortho*‐methyl derivative **1k** showed no reactivity at all (**12** was not detected, and **1k** was fully recovered). Halogen‐substituted substrates bearing bromine (**1l**) or chlorine (**1m**) at the *ortho*‐position consistently provided the desired products only with low yields. We also evaluated the reactivity of unsubstituted 1‐phenylethanol **1n**, which was smoothly converted to provide acetophenone **15** in a decent yield. By contrast, the disubstituted aromatic substrate **1o** resulted in complete recovery of the starting material (no **16** formed). Finally, conformationally constrained benzo‐fused substrates **1p**,**q** furnished the corresponding oxidation products in modest to good yields (**17,18**).

**TABLE 1:** Substrate scope for the aerobic oxidation of benzylic alcohols (**1**) to the corresponding carbonyl compounds (**2‐26**).^a^

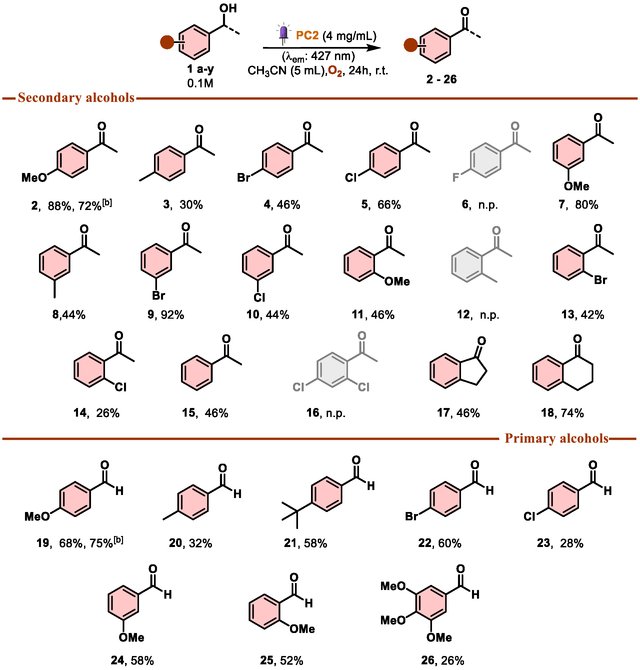

a Reaction conditions: A solution of benzylic alcohol **1** (0.5 mmol, 1 equiv.) in CH_3_CN (5 mL, 0.1 M) in the presence of **PC2** (20 mg) was bubbled with O_2_ (~5 min.), capped and then irradiated with a LED lamp (40 W; emission centered at 427 nm) at room temperature for 24 h. Yields of isolated product after column chromatography on silica gel (cyclohexane/ethyl acetate mixture as eluant) have been reported (see general procedure **GP**‐***b*** in Section 3.1 for further details).

b Gram scale reaction (10 mmol; see general procedure **GP**‐***c*** in Section 3.1 for further details).

After investigating secondary benzylic alcohols, we then turned our attention to primary derivatives, which pose a selectivity issue, potential products being either aldehydes and/or the corresponding carboxylic acids. As with secondary alcohols, we began by examining a series of *para*‐substituted substrates (**1r–v**), all of which underwent selective oxidation to the corresponding aldehydes in satisfactory yields (**19–23**). Prolonged irradiation (40 h) of alcohol **1r** did not affect the reaction outcome, with aldehyde **19** as the only product formed (see Supporting Information for additional details). The *meta*‐methoxy‐substituted substrate **1w** also afforded the desired aldehyde in good yield (**24**). Consistent with the trend observed for secondary benzylic alcohols, introducing the same substituent at the *ortho*‐position (substrate **1x**) resulted in a ∼50% yield of **25**. Finally, a trisubstituted aromatic scaffold was evaluated (**1y**) and successfully converted to the corresponding product, although only low conversion of the alcohol substrate and a modest yield of **26** were obtained.

Following exploration of the reaction scope, the preparation of compounds **2** and **19** was successfully scaled (10 mmol). The desired products were consistently obtained in 72% and 75% isolated yield, respectively, with >1 g of compound **2** isolated. The robustness of the method was further assessed through catalyst recycling experiments (Figure 2). In the synthesis of compound **2**, the catalytic system maintained high performance over three recovery cycles, consistently delivering yields of 88% (Figure ‍2a,b). After each cycle, the integrity of the heterojunction was verified by XRD analysis, confirming the stability of the material structure (Figure 2c; see Section S1.4 for full details). A slight decrease in the intensity of the perovskite reflections can be related to a progressive reduction in crystallinity. Inductively coupled plasma‐mass spectrometry (ICP‐MS) analysis after the first reaction run revealed a limited Pb leaching of approximately 8% of the initial Pb content (see Section S1.5).

**FIGURE 2:**
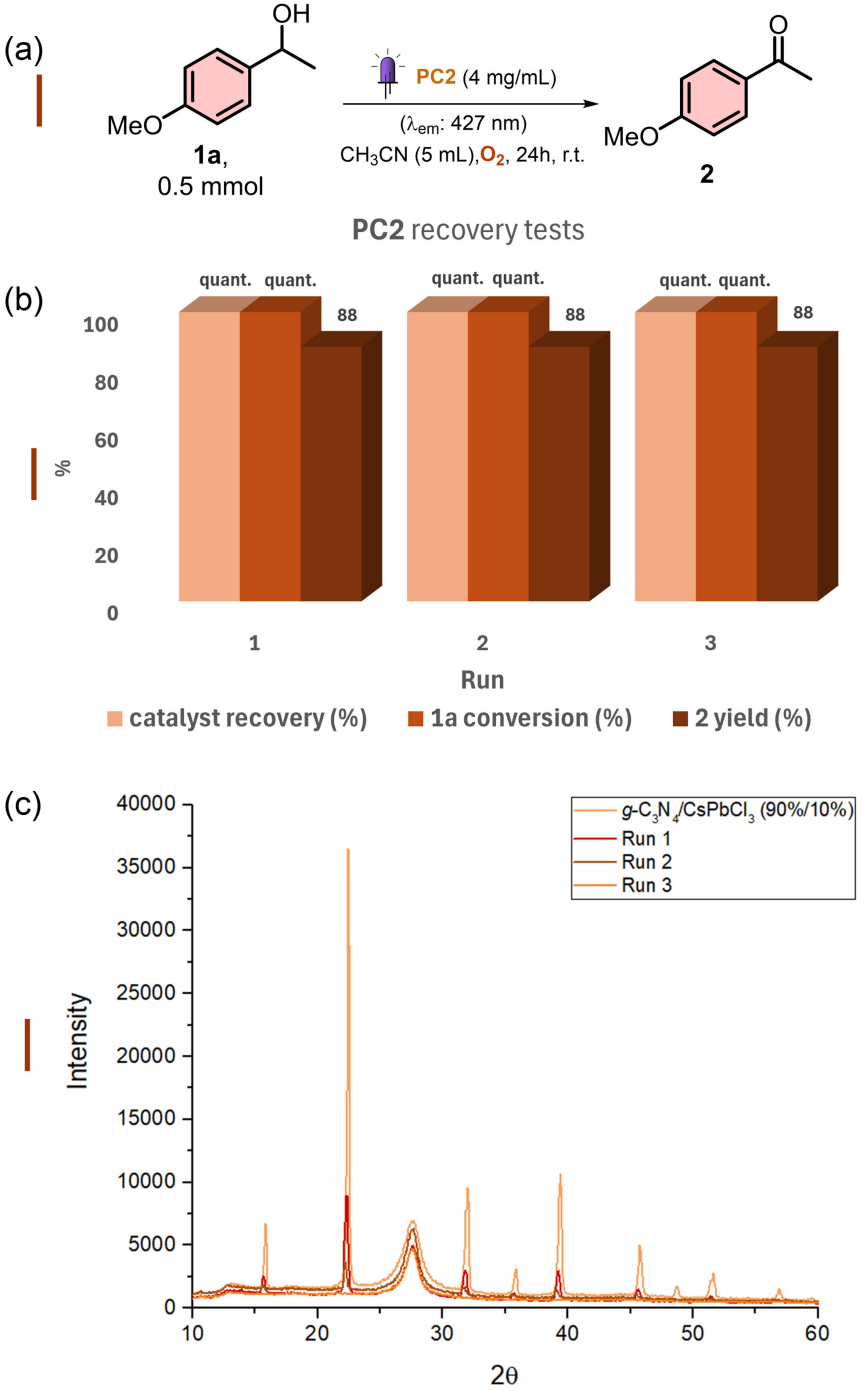
Results from multiple recycling of the *g*‐C_3_N_4_/CsPbCl_3_ (90%/10%) catalyst (**PC2**): (a) model oxidation reaction of **1a** into **2** tested; (b) conversion of substrate **1a**, yield of product **2** and catalyst recovery (%) at the three catalytic runs; and (c) XRD diffraction patterns of pristine and recovered catalyst. Reference pattern for CsPbCl_3_ in agreement with PDF card 75‐0408.

Next, mechanistic investigations were undertaken to gain insight into the reaction pathway (see Section S1.6 for full details). The addition of the radical scavenger 2,2,6,6‐tetramethylpiperidine *N*‐oxyl (TEMPO), known to intercept free‐radical intermediates [37], resulted in a concentration‐dependent decrease in the conversion of **1a** to **2**. In particular, only 38% **1a** conversion was achieved in the presence of 1.5 equiv. of TEMPO (Scheme 1a). These results are consistent with the involvement of radical species in the reaction mechanism.

**SCHEME 1:**
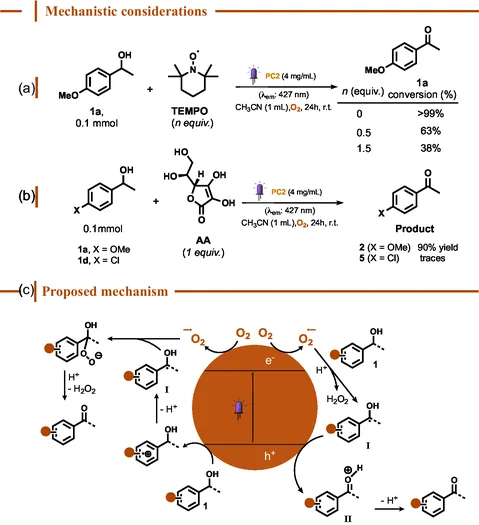
Mechanistic considerations supporting a radical pathway and proposed mechanistic scenario.

Given that molecular oxygen was found to be essential for productive reactivity, we next investigated the possible involvement of reactive oxygen species (ROS). To this end, ascorbic acid (AA), a well‐known scavenger of the superoxide anion radical (O_2_^•–^), was employed as an additive [37]. The effect of AA was examined using 1‐(4‐methoxyphenyl)ethan‐1‐ol (**1a**) and 1‐(4‐chlorophenyl)ethan‐1‐ol (**1d**), two substrates displaying markedly different oxidation potentials [13]. Strikingly, distinct outcomes were observed: whereas the addition of 1 equiv. of AA had no appreciable effect on the conversion of **1a** into **2**, the same amount of AA completely suppressed the formation of **5** from **1d** (Scheme 1b). These results suggest that different activation pathways may operate depending on the redox properties of the substrate. Further evidence of the electronic interaction between the two semiconductor components was obtained from steady‐state photoluminescence (PL) measurements (Figure S4). Compared with pristine *g*‐C_3_N_4_, the normalized PL spectrum of **PC2** shows a clear redistribution of the emission profile, with an increased relative contribution of the high‐energy component. This behavior indicates that the coupling with the perovskite modifies the excited‐state relaxation pathways of carbon nitride, supporting interfacial electronic coupling within the heterojunction.

Based on the redox properties of the *g*‐C_3_N_4_/CsPbCl_3_ heterojunction photocatalyst (estimated *E*_VB_ of +1.26 V vs. SCE and *E*_CB_ −1.29 V vs. SCE) [38] and taking into account the results of the above‐mentioned mechanistic experiments, the aerobic oxidation of benzylic alcohols to the corresponding carbonyl compounds is proposed to proceed according to the pathways outlined in Scheme 1c. For substrates characterized by relatively low oxidation potentials (e.g., $E_{p/2}^{\text{OX}}$(**1a**) = +1.62 V vs. SCE) [13], a possible pathway involving interaction with photogenerated holes or interfacial excited states is suggested. Such activation may involve defect‐mediated processes beyond a simple outer‐sphere electron transfer to holes accumulated on *g*‐C_3_N_4_, as proposed for related MHP/carbon nitride heterojunctions [29, 39]. This step generates radical cation **1a** ^**•+**^, which upon deprotonation affords ketyl radical **I**. Subsequent reaction with molecular oxygen [40] and liberation of hydrogen peroxide ultimately deliver the carbonyl product (see Scheme 1c, left part).

In contrast, substrates exhibiting significantly higher oxidation potentials (e.g., $E_{p/2}^{\text{OX}}$(**1d**) = +2.24 V vs. SCE) [13] are proposed to follow an indirect pathway. In this case, photogenerated electrons in the conduction band first reduce molecular oxygen to the superoxide radical anion [41] O_2_^•–^ (*E* ^0^(O_2_/O_2_^•–^ ∼ −0.33 V vs. SCE) [42]. In turn, the latter species can abstract, either directly or through its protonated form hydroperoxyl radical (HOO^•^) [43], a hydrogen atom from the benzylic position to generate ketyl radical **I**. Such intermediates, characterized by an extremely low oxidation potential ($E_{p/2}^{\text{OX}}$ in the −0.46 to −0.26 V vs. SCE have been determined for benzyl‐type *α*‐hydroxyalkyl radicals) [44], are then oxidized by photogenerated holes in the valence band to finally deliver the oxidized product of interest together with hydrogen peroxide as a byproduct (see Scheme 1c, right part). Notably, this dual mechanistic scenario is fully consistent with the results obtained in the presence of AA, which selectively suppresses the oxidation of the more challenging substrate **1d** while leaving the conversion of **1a** essentially unaffected (see Scheme 1b).

A final comment regarding the chemoselectivity observed with primary benzylic alcohols is warranted. In principle, the first‐formed aldehyde products could undergo a subsequent oxidation step to furnish the corresponding carboxylic acids. However, this pathway is not observed under the present conditions, even upon prolonged irradiation. On the one hand, aldehydes are significantly more difficult to oxidize than the parent alcohols (e.g., the oxidation potential for benzaldehyde is +2.30 V vs. SCE) [45], rendering direct single‐electron oxidation unfavorable. On the other hand, hydrogen‐atom abstraction from the formyl C─H bond is intrinsically less favorable than from the benzylic position of the corresponding alcohol, as reflected by the higher bond dissociation energy of benzaldehyde (89 kcal·mol^–1^) [46] compared with benzyl alcohol (79 kcal·mol^–1^) [46]. Collectively, these factors suppress overoxidation and account for the selective formation of aldehydes under the reaction conditions.

In terms of reaction scope, different outcomes are observed depending on the actual structure of the substrate. As a general trend, electron‐donating substituents on the aromatic ring appear to improve the performance of the described oxidation protocol, possibly because such substrates have lower oxidation potentials and can benefit from both of the mechanistic pathways described above. Conversely, steric hindrance tends to have a negative impact as alcohols bearing *ortho*‐substituents typically afford lower yields of the corresponding carbonyl products. Additionally, the presence of benzylic C─H positions is generally associated with poor performance, possibly owing to their competitive, yet unproductive, activation.

## 3 Conclusions

In conclusion, this work presents a versatile heterogeneous photocatalytic protocol based on carbon nitride/lead perovskite heterojunctions. The methodology enables the aerobic oxidation of benzylic alcohols, selectively affording either ketones or aldehydes depending on the substrate. The reaction proceeds under mild conditions, requires no additives, and can be readily performed on a gram scale. Moreover, the robustness of the photocatalytic material allows for its reuse over multiple cycles without any significant loss of activity or structural integrity.

Mechanistic investigations revealed that the oxidation proceeds through radical pathways involving photogenerated charge carriers and ROS. Depending on the substrate redox properties, oxidation may occur either through direct single‐electron transfer from the alcohol to the photoexcited heterojunction or through a superoxide‐mediated pathway, ultimately converging to common ketyl radical intermediates. These findings provide valuable insight into the reactivity of MHP‐based heterojunctions in photocatalysis applied to organic transformations.

More broadly, this work demonstrates that coupling MHPs with carbon nitride offers an effective strategy to enhance charge separation and exploit the tunable redox properties of perovskite materials for sustainable organic synthesis. We anticipate that this approach will stimulate the development of new perovskite‐based heterojunction photocatalysts for selective oxidation reactions and other value‐added photochemical transformations.

## Funding

This study was supported by the European Union’s Horizon Europe research and innovation program under Grant Agreement 101046836 (project acronym “CATART”).

## Conflicts of Interest

The authors declare no conflicts of interest.

## Supporting information

Supplementary Material

## Data Availability

The data that support the findings of this study are available in the supplementary material of this article.
