# Supplementary material for: Engineering g‐C3N4/CsPbCl3 Heterojunctions for Selective Photocatalytic Oxidation of Benzylic Alcohols
Source: ChemSusChem. 2026 Oct 14;19(20):e71146. doi: 10.1002/cssc.71146 (PMC13667783; doi:10.1002/cssc.71146)

Supporting information for

Engineering *g*-C_3N_4/$CsPbCl_3$ Heterojunctions for Selective Photocatalytic Oxidation of Benzylic Alcohols

Camilla Callegari,[a] Emanuele Bosetti,[a,b] Giulia Giovilli,[b] Davide Ravelli,[a,*] Lorenzo Malavasi[b,*]

[a] *PhotoGreen Lab, Department of Chemistry, University of Pavia, Viale Taramelli 12, 27100 Pavia (PV), Italy. E-mail:* davide.ravelli@unipv.it, *web:* https://photogreenlab.unipv.it/

[b] *Energy and Materials Chemistry Group, Department of Chemistry and INSTM, University of Pavia, Viale Taramelli 16, 27100 Pavia (PV), Italy. E-mail:* lorenzo.malavasi@unipv.it, *web:* https://malavasi.unipv.it/

TABLE OF CONTENTS (53 pages)

1. Data cited in the main text — p. S2

- **1.1 Catalysts characterization — p. S2**
- **1.2 Chart of starting materials — p. S5**
- **1.3 Optimization of reaction conditions — p. S6**
- **1.4 Catalyst recovery — p. S9**
- **1.5 Metal leaching evaluation — p. S10**
- **1.6 Mechanistic investigations — p. S11**

2. Experimental details — p. S18

- **2.1 General information — p. S18**
- **2.2 General procedure for the synthesis of the catalyst (GPC) — p. S19**

3. Photocatalyzed aerobic oxidation reactions — p. S20

- **3.1 General procedures for photocatalyzed aerobic oxidation reactions — p. S20**
- **3.2 Characterization data for products 2-26 — p. S23**

4. References — p. S31

5. Copy of ^1H and ^{13}C NMR spectra — p. S32

1. Data cited in the main text

1.1 Catalysts characterization

The *g*-C_3N_4/$CsPbCl_3$ heterojunctions were prepared with the same procedure adopted for analogous systems in the past by our group.[1] The following weight ratios between nano exfoliated *g*-C_3N_4 and $CsPbCl_3$ have been investigated: 0, 10, 30, 50, 90 and 100 (these values do refer to the $\%_{W/W}$ of perovskite in the material).

The obtained samples were characterized by room temperature X-Ray Diffraction. The patterns of the pristine carbon nitride and perovskite samples are reported in the main text (**Figure 1b**). However, **Figure S1** reports the diffraction pattern of $CsPbCl_3$ against the calculated cubic structure (lattice parameter 5.71 Å).

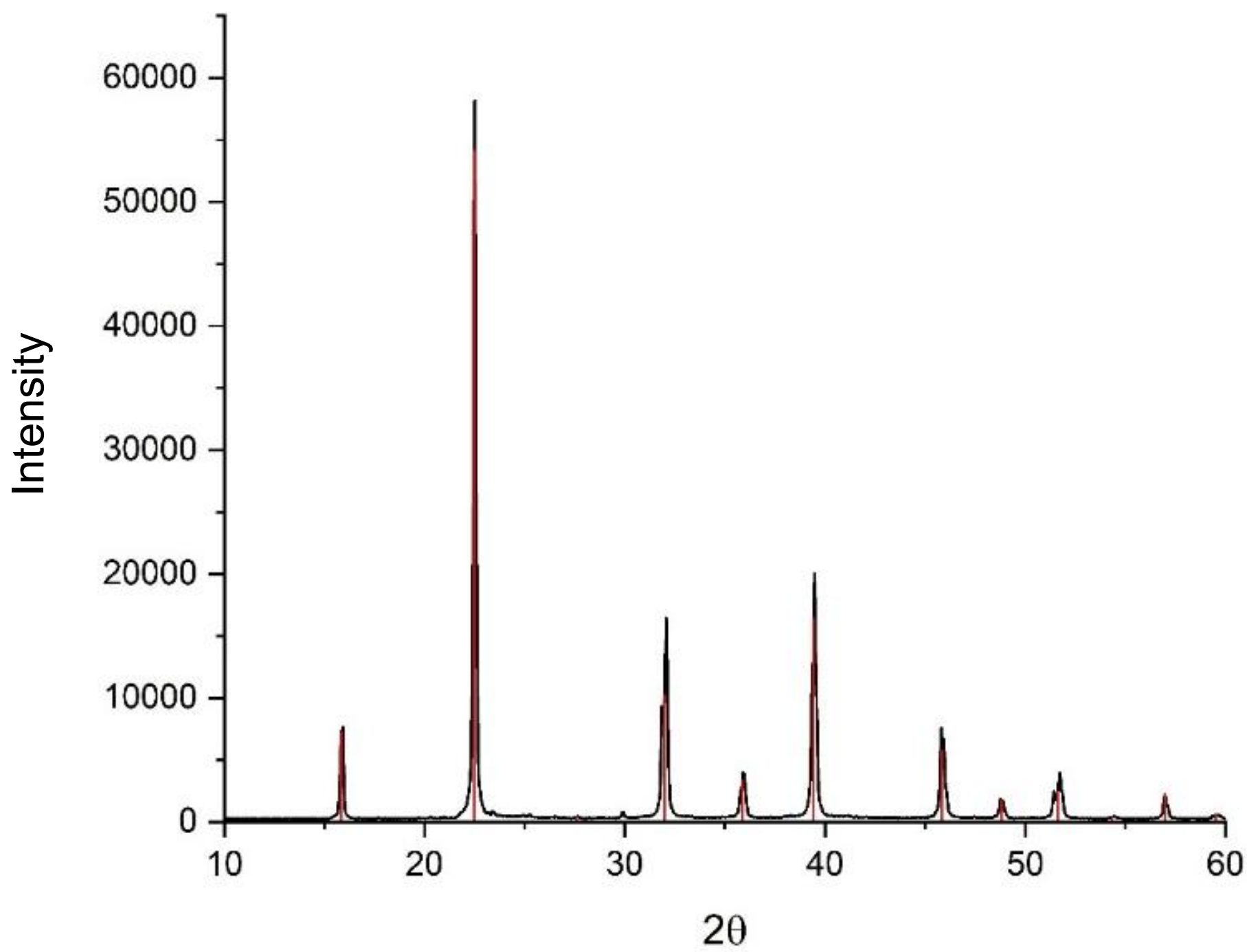

Figure S1. XRD pattern of $CsPbCl_3$ (black line) superimposed to the calculated cubic pattern (PDF card 75-0408).

XRD patterns of the heterojunctions are reported in **Figure 1c** of the main text.

Figure S2 reports the UV-Vis spectra of nano-exfoliated *g*-C_3N_4 (**PC1**; panel a) and $CsPbCl_3$ (**PC6**; panel b), respectively. From the Tauc plot (reported in the inset), the optical bandgap for the perovskites has been found to be around 2.8 eV while that of carbon nitride around 2.7 eV.

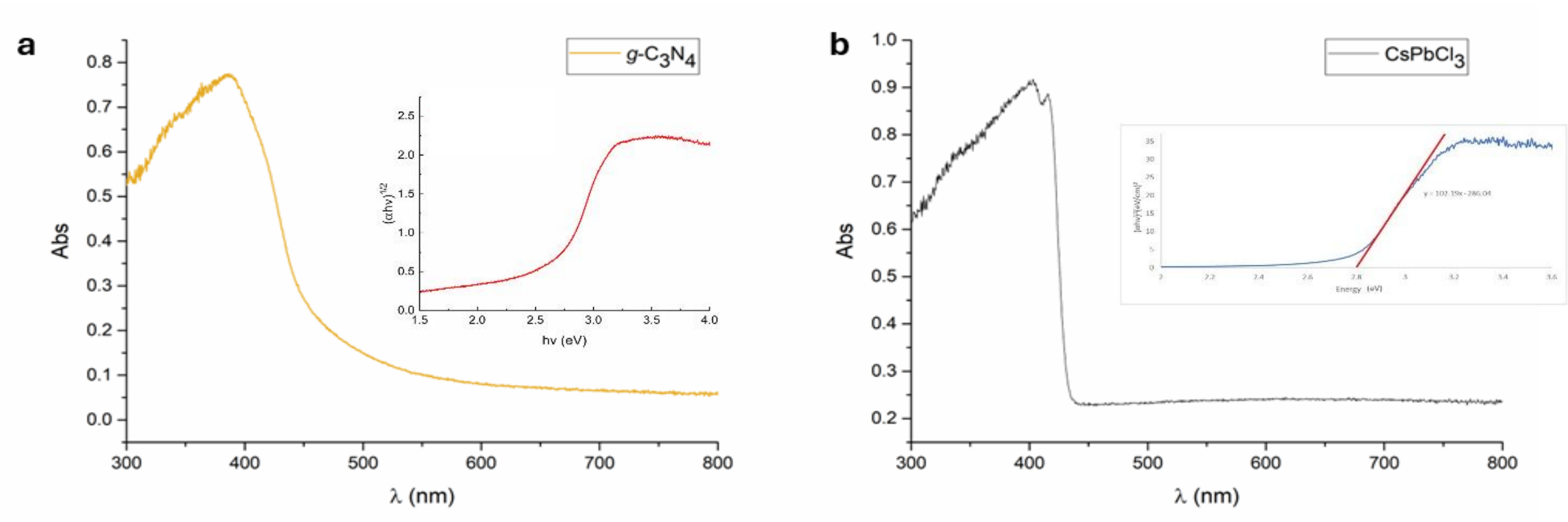

Figure S2. UV-Vis spectra of: a) *g*-C_3N_4 and b) $CsPbCl_3$.

The UV-Vis spectra of the complete series of the prepared *g*-C_3N_4/$CsPbCl_3$ heterojunctions (**PC2-5**), and pure $CsPbCl_3$ (**PC6**) are reported in **Figure S3**. As for the **PC2-5** heterojunctions, they all show a strong absorption at the edge between UV and visible light, with a tail extending up to *ca*. 600 nm. As a rule of thumb, it can be concluded that the composites show an absorption spectrum similar to the most abundant component.

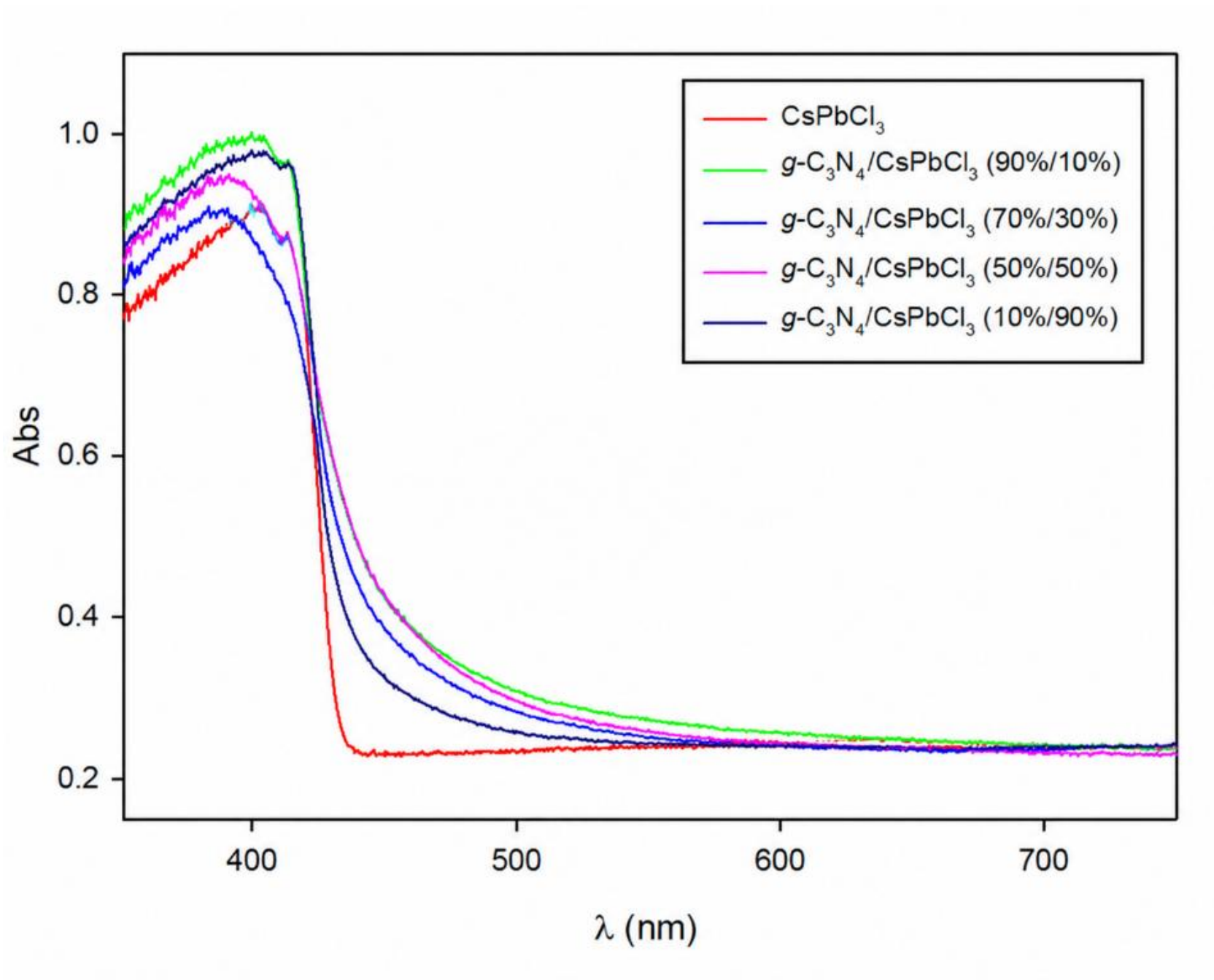

Figure S3. UV-Vis spectra of the **PC2-5** heterojunction together with that of $CsPbCl_3$ (**PC6**).

Figure S4 shows the steady-state photoluminescence (PL) spectra of pristine *g*-C_3N_4 (**PC1**) and the best-performing *g*-C_3N_4/$CsPbCl_3$ (90:10$_{W/W}$) heterojunction (**PC2**). Compared with pristine *g*-C_3N_4, the best performing heterojunction **PC2** shows a clear modification of the emission profile, with an increased relative contribution of the high-energy component associated with near-band-edge transitions of *g*-C_3N_4.

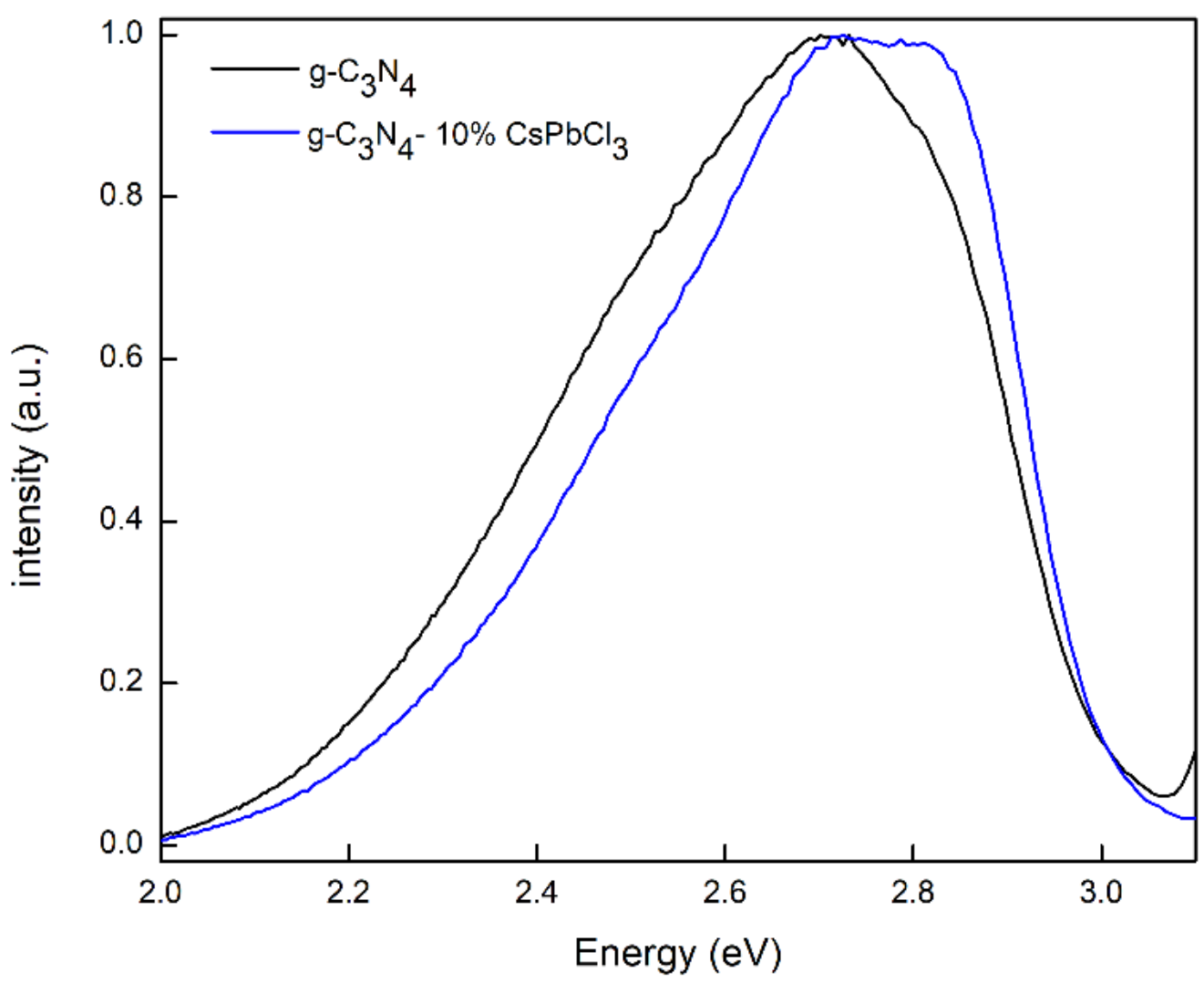

Figure S4. Normalized steady-state photoluminescence (PL) spectra of pristine *g*-C_3N_4 (**PC1**) and the best-performing *g*-C_3N_4/$CsPbCl_3$ (90:10$_{W/W}$) heterojunction (**PC2**).

1.2 Chart of starting materials

Compounds shown in gray color refer to limitations of the substrate scope.

Starting materials (benzylic alcohols) used in Table 1

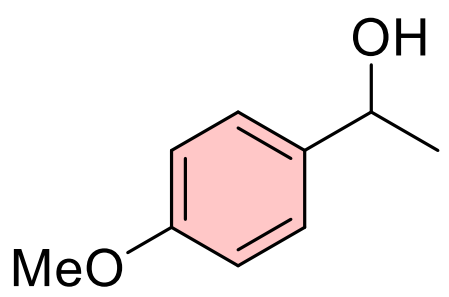

1a

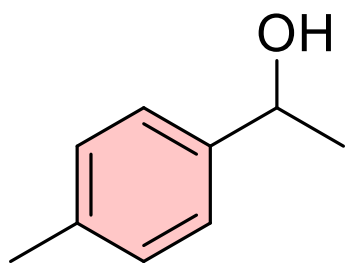

1b

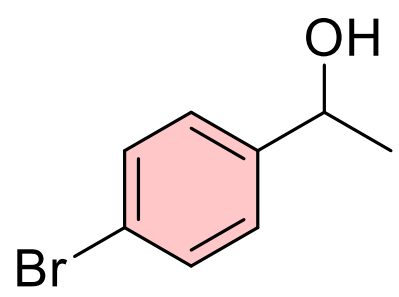

1c

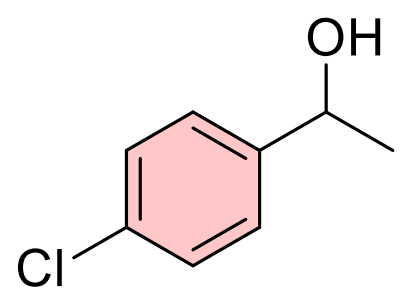

1d

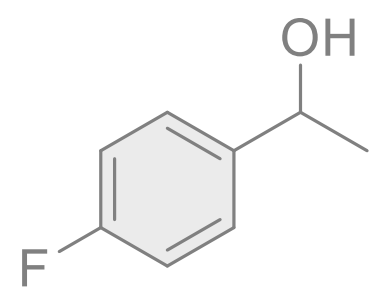

1e

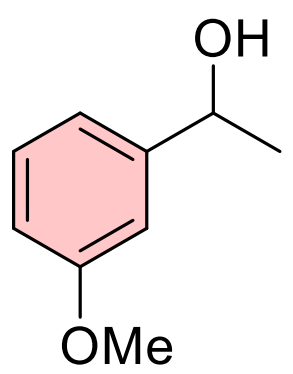

1f

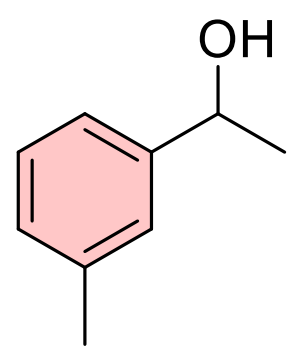

1g

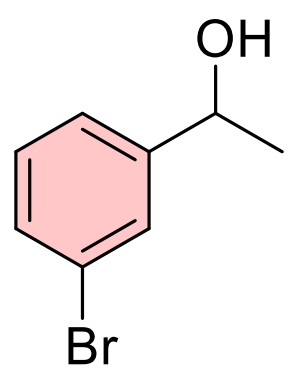

1h

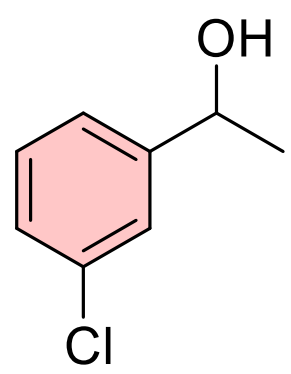

1i

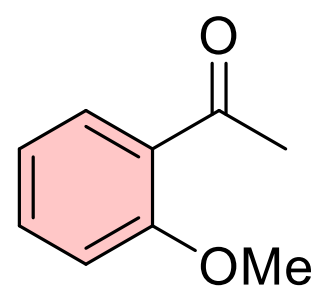

1j

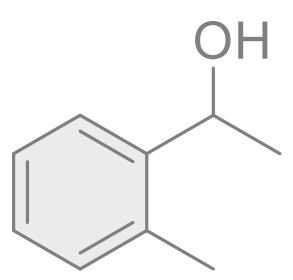

1k

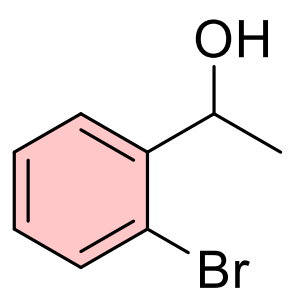

1l

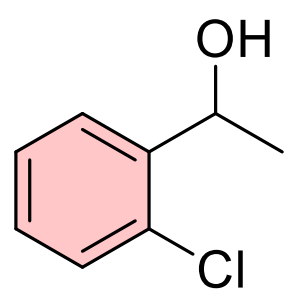

1m

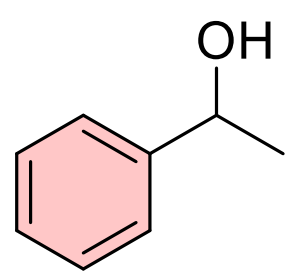

1n

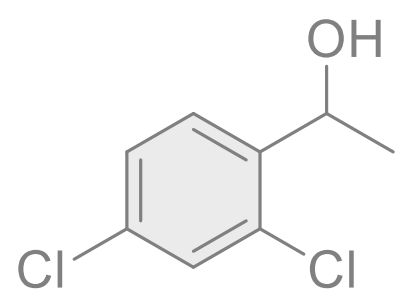

1o

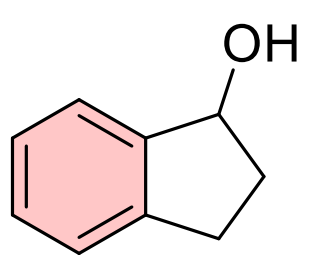

1p

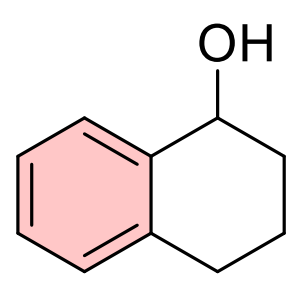

1q

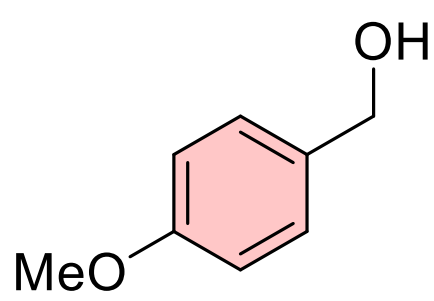

1r

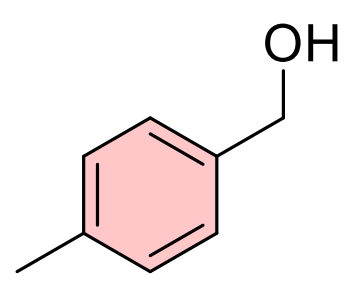

1s

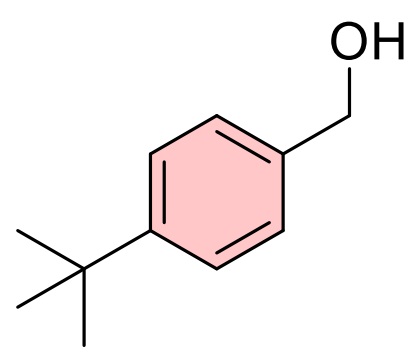

1t

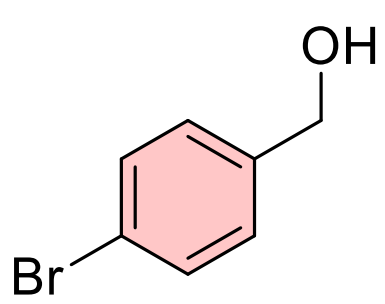

1u

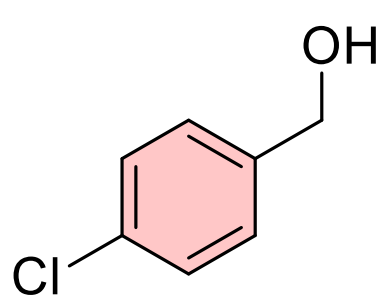

1v

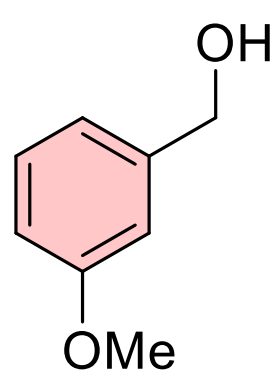

1w

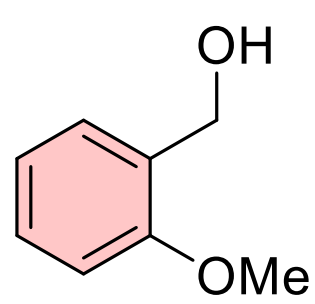

1x

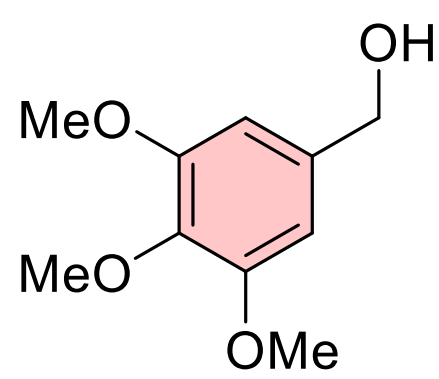

1y

1.3 Optimization of reaction conditions

The reaction conditions were optimized using the experimental setup described in the **GP-*a*** method (see **SECTION 3.1** below), by studying the model reaction involving 1-(4-methoxyphenyl)ethan-1-ol (**1a**; 0.1 mmol, 0.1 M) to prepare the oxidation product 1-(4-methoxyphenyl)ethan-1-one (**2**). Yields reported in the **TABLEs S1-S6** were determined via GC analysis using 1,3,5-trimetoxybenzene as external standard (0.1 mmol, 0.1 M, 1.0 equiv.), unless otherwise specified.

Table S1. Catalyst screening.

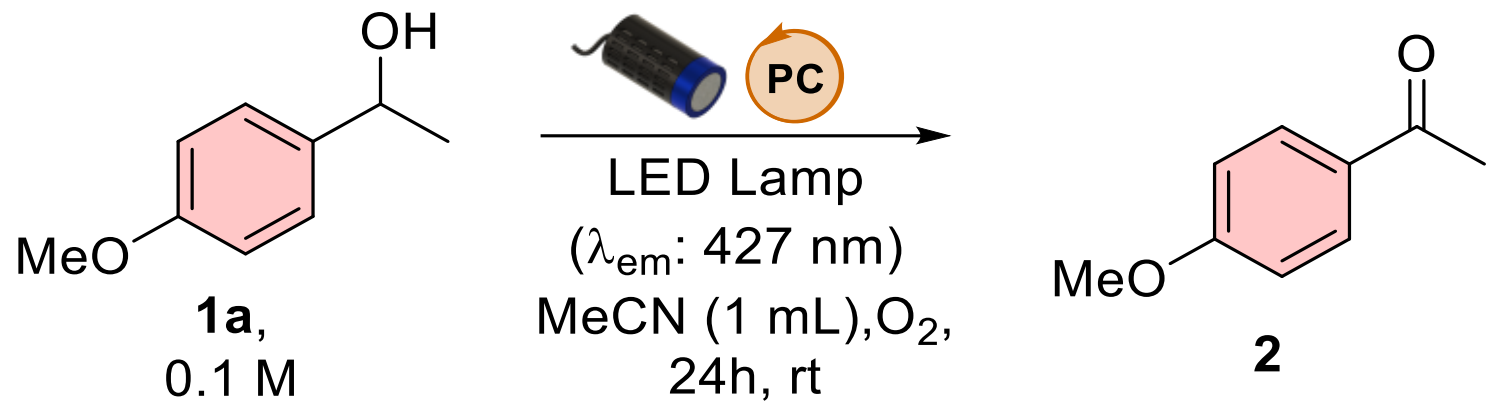

Entry	PC	Catalyst (4 mg)	**2** Yield
1	**PC1**	*g*-C_3N_4	65%
**2**	**PC2**	***g*-C_3N_4/$CsPbCl_3$ 90%/10%**	**90%, 88%[a]**
3	**PC3**	*g*-C_3N_4/$CsPbCl_3$ 70%/30%	81%
4	**PC4**	*g*-C_3N_4/$CsPbCl_3$ 50%/50%	41%
5	**PC5**	*g*-C_3N_4/$CsPbCl_3$ 10%/90%	31%
6	**PC6**	$CsPbCl_3$	traces

[a] isolated yield of **2** after column chromatography on silica gel (hexane/ethyl acetate as eluants); see method GP-*b* in Section 3.1.

Table S2. Catalyst loading.

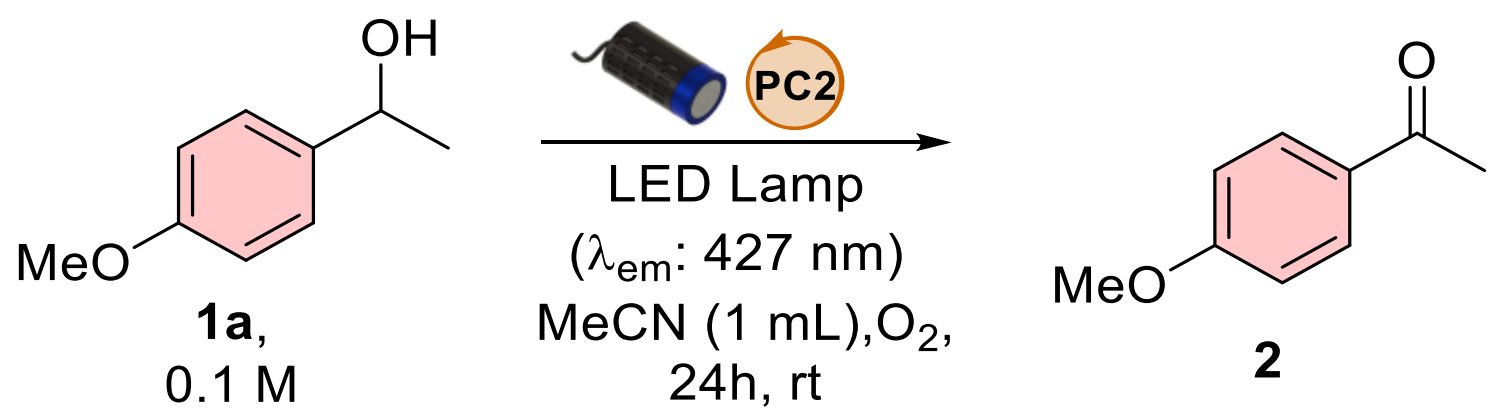

Entry	**Catalyst loading**	**2** Yield
1	2 mg	32%
2	3 mg	78%
**3**	**4 mg**	**90%, 88%[a]**
4	5 mg	88%
5	7 mg	71%

[a] isolated yield of **2** after column chromatography on silica gel (hexane/ethyl acetate as eluants); see method GP-*b* in Section 3.1.

Table S3. Solvent screening.

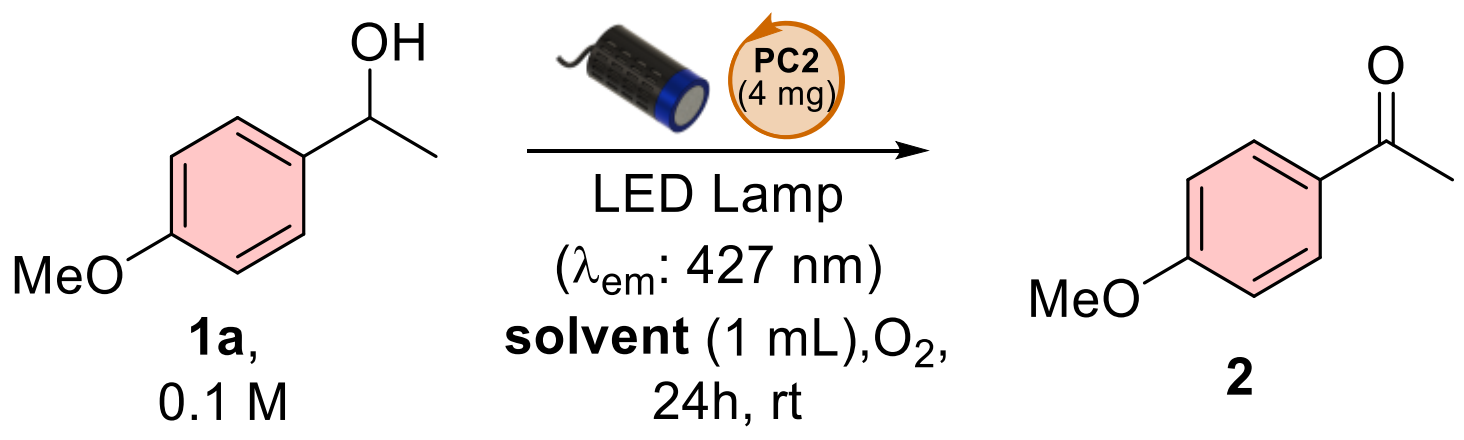

Entry	**Solvent**	**2** Yield
1	MeOH	62%
2	EtOAc	82%
**3**	**MeCN**	**90%, 88%[a]**
4	THF	22%
5	$MeCN/H_2O$ 95:5	71%

[a] isolated yield of **2** after column chromatography on silica gel (hexane/ethyl acetate as eluants); see method GP-*b* in Section 3.1.

Table S4. Wavelength screening.

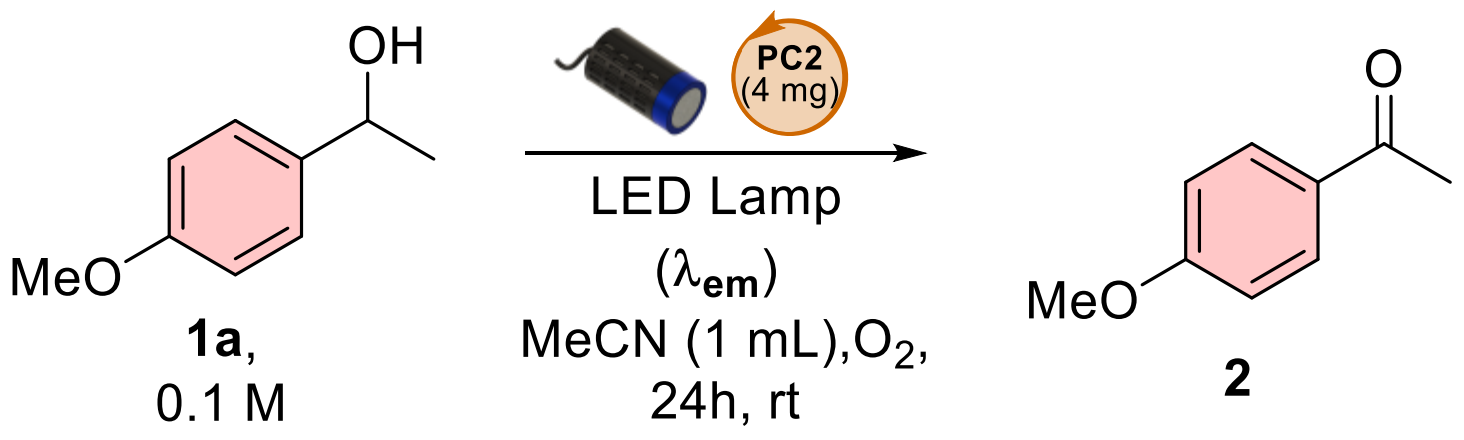

Entry	λ_{em}	**2** Yield
1	390 nm	36%
2	405 nm	60%
**3**	**427 nm**	**90%, 88%[a]**
4	456 nm	80
5	525 nm	n.d.

[a] isolated yield of **2** after column chromatography on silica gel (hexane/ethyl acetate as eluants); see method GP-*b* in Section 3.1. n.d.: not detected.

Table S5. Reaction atmosphere.

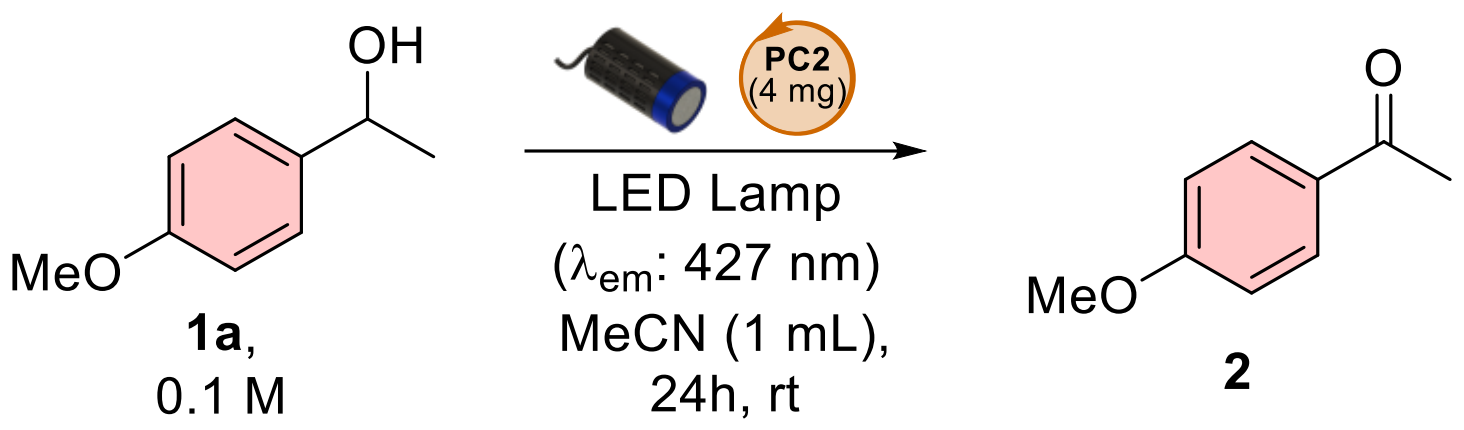

Entry	**Atmosphere**	**2** Yield
**1**	O_2	**90%, 88%[a]**
2	N_2	7%
3	Air	33%
4	Ar	traces

[a] Isolated yield of **2** after column chromatography on silica gel (hexane/ethyl acetate as eluants); see method GP-*b* in Section 3.1.

Table S6. Control experiments.

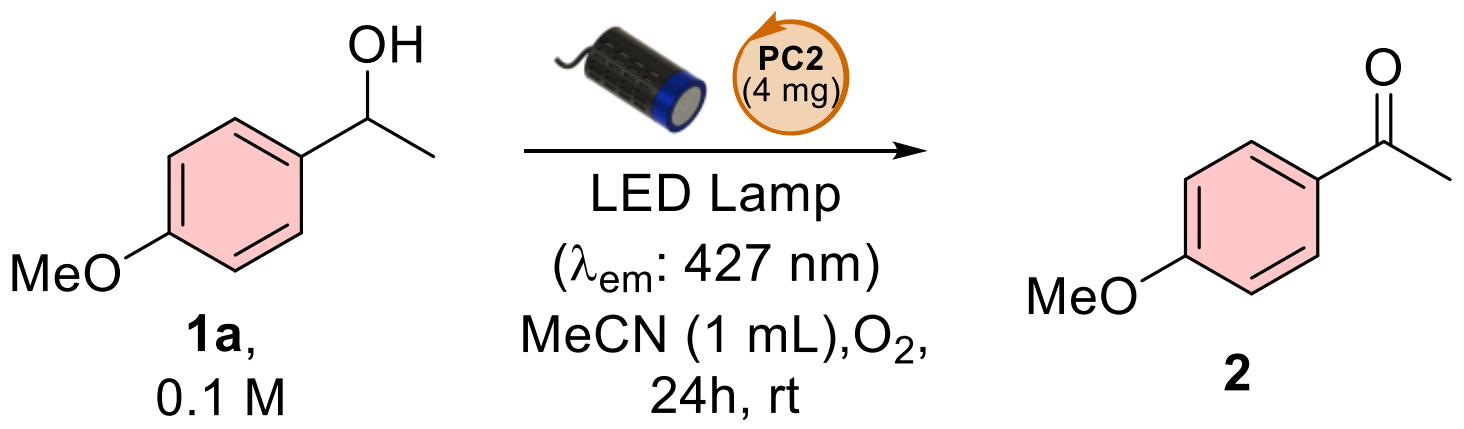

Entry	**Control experiment**	**2** Yield
1	no light	n.d.
2	no **PC**	n.d.
3	no light, heating (60°C)	n.d.
4[a]	**PC1**/**PC6** 90:10%$_{w/w}$ ratio	65%

[a] A *g*-C_3N_4/$CsPbCl_3$ physical mixture has been used in the role of heterogeneous photocatalyst. n.d.: not detected.

1.4 Catalyst recovery

To demonstrate the recyclability of the *g*-C_3N_4/$CsPbCl_3$ (90%/10%) catalyst (**PC2**), we run the model reaction involving 1-(4-methoxyphenyl)ethan-1-ol (**1a**; 0.1 mmol, 0.1 M) to prepare the oxidation product 1-(4-methoxyphenyl)ethan-1-one (**2**) according to the conditions described in general procedure **GP-*b***. The reported yield values do refer to the isolated yield of **3** obtained after each run.

After each reaction run, the solids were centrifuged for 10 minutes at 10.000 rpm, and the supernatant was subjected to the purification described in general procedure **GP-*b*** to isolate **2**. For each cycle, the catalyst was washed with acetonitrile, centrifuged, and then dried under vacuum overnight. The recovered sample of the *g*-C_3N_4/$CsPbCl_3$ (90%/10%) catalyst (**PC2**) was then recovered for 3 runs, demonstrating unchanged performance over each run (see **TABLE S7**).

TABLE S7. Results from multiple recycling of the *g*-C_3N_4/$CsPbCl_3$ (90%/10%) catalyst.

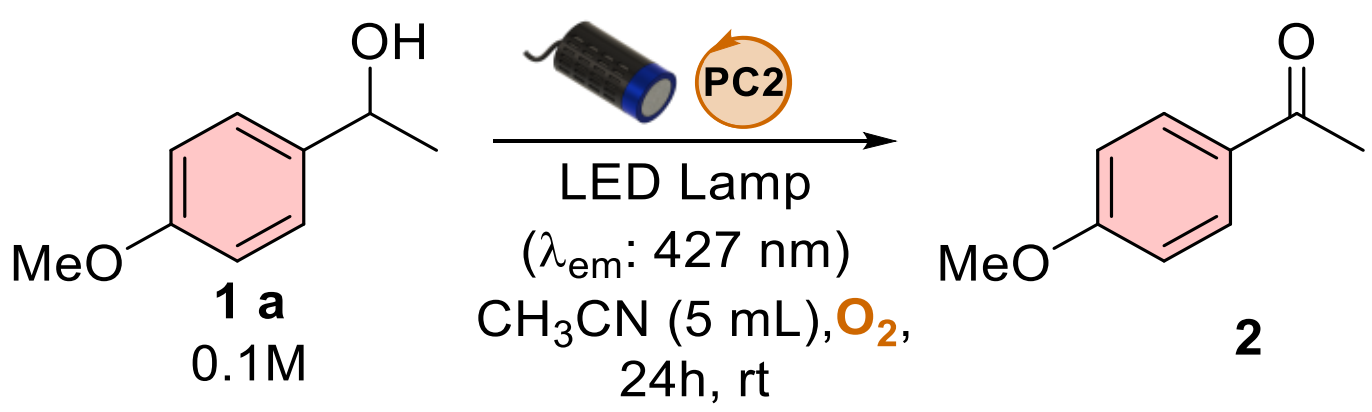

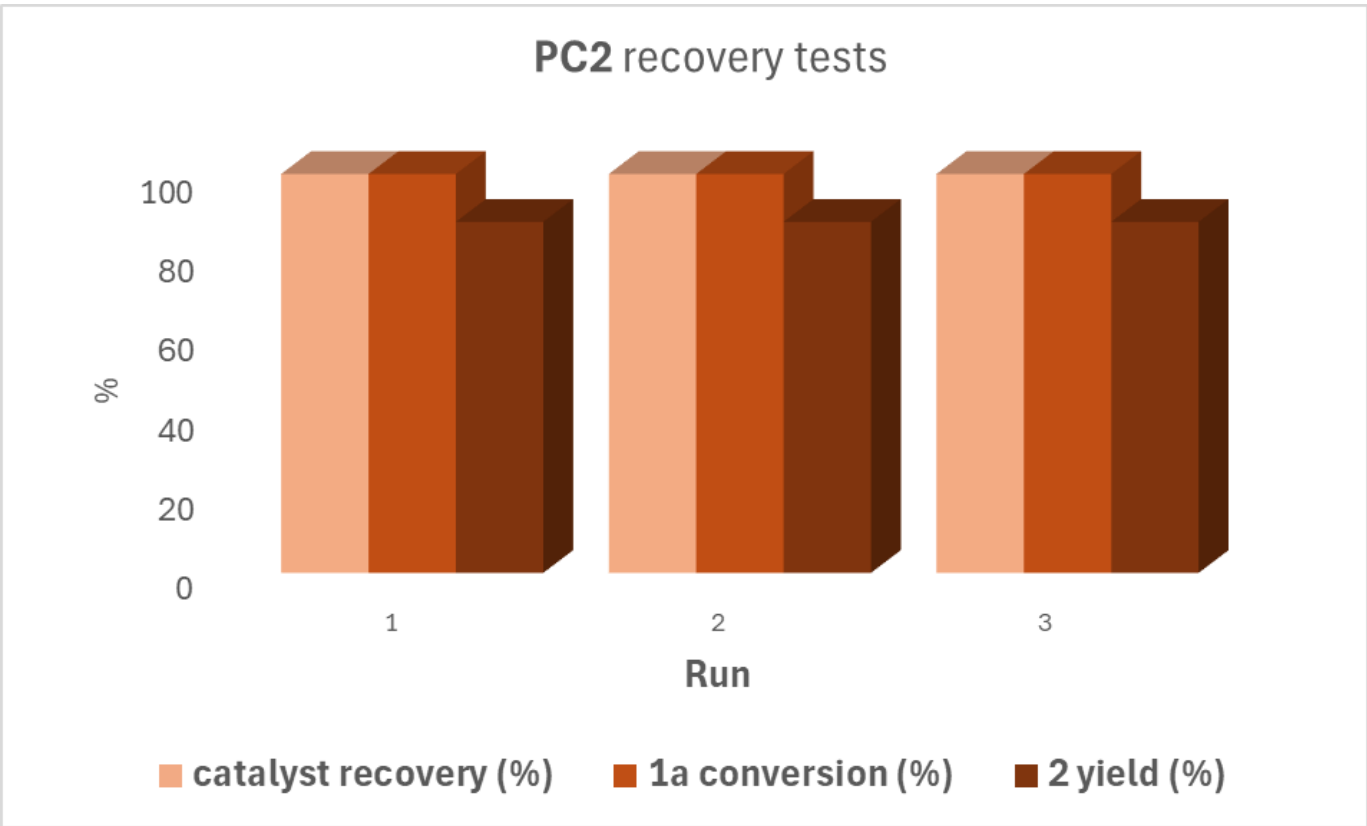

Run	2 Yield (%)
1	88
2	88
3	88

1.5 Metal leaching evaluation

The leaching of lead (Pb) from the reference catalyst (**PC2**; *g*-C_3N_4/$CsPbCl_3$, 90%/10%) was evaluated by inductively coupled plasma (ICP) analysis of the liquid phase obtained after the catalytic reaction involving 1-(4-methoxyphenyl)ethan-1-ol (**1a**; 0.1 mmol, 0.1 M) to prepare the oxidation product 1-(4-methoxyphenyl)ethan-1-one (**2**) according to the conditions described in general procedure **GP-*b*** (see reaction equation in **TABLE S7**).

Following the reaction, the catalyst was separated from the crude mixture by filtration (nylon; 0.45 μm), then the organic solvent (HPLC-type acetonitrile) was removed from the resulting liquid sample by rotary evaporation, then diluted with HPLC-type water. The resulting solution was finally analyzed by ICP to determine the concentration of the leached metal species. The amount of metal released from the catalyst was quantified based on the calibration curves and, where appropriate, normalized to the initial metal content of the catalyst. By comparison, the amount of lead (Pb) metal present in the employed solvent was also evaluated. Full details are reported in the table below.

**Sample**	**Pb concentration (ppm, mg·L^{-1})**
Crude reaction mixture	16
MeCN	< LOQ
H_2O	< LOQ

LOQ: limit of quantification

1.6 Mechanistic investigations

1.6.1 Experiments with scavengers for reactive species

To have insights into the reaction mechanism, we focused our attention on two different processes, namely:

- The reaction involving 1-(4-methoxyphenyl)ethan-1-ol (**1a**; 0.1 mmol, 0.1 M) to prepare the oxidation product 1-(4-methoxyphenyl)ethan-1-one (**2**);
- The reaction involving 1-(4-chlorophenyl)ethan-1-ol (**1d**; 0.1 mmol, 0.1 M) to prepare the oxidation product 1-(4-chlorophenyl)ethan-1-one (**5**).

Both processes were run according to the conditions described in general procedure **GP-*a*** (see **SECTION 3.1** below) in the presence of scavengers known to selectively quench different reactive species.[2]

TEMPO as scavenger The reaction involving 1-(4-methoxyphenyl)ethan-1-ol (**1a**; 0.1 mmol, 0.1 M) to prepare the oxidation product 1-(4-methoxyphenyl)ethan-1-one (**2**) was run in the presence of the additive TEMPO (2,2,6,6-tetramethylpyrrolidine *N*-oxyl) as free radical scavenger. Different experiments were performed, as indicated in **Scheme S1**, while the corresponding reaction crudes have been subjected to GC-MS analysis (**Figure S5**). The resulting GC traces were compared with those of selected reference compounds (**Figure S6**).

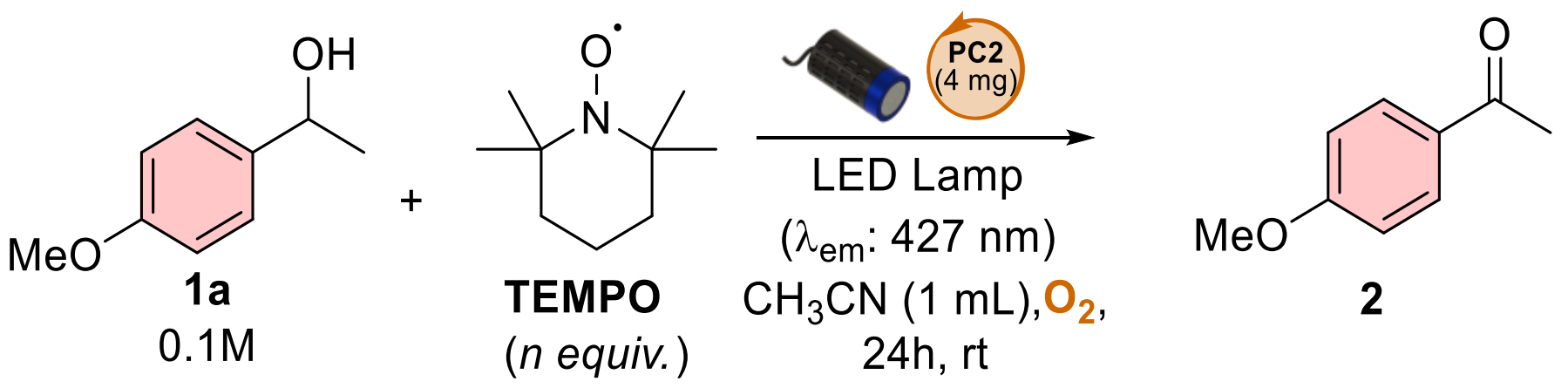

TEMPO *n* (equiv.)	1a Conversion (%)
0	>99%
0.5	63%
1.5	38%

Scheme S1. Experiments performed to evaluate the effect of the TEMPO additive on the model reaction involving 1-(4-methoxyphenyl)ethan-1-ol (**1a**).

Upon inspection of the GC charts in **Figure S5**, it is apparent the effect of the TEMPO additive on the reaction course, clearly demonstrating the radical nature of the reported process. Thus, the model reaction in **Scheme S1** (0 equiv. TEMPO added) cleanly leads to product **2** (peak at min. 12.25 in

Figure S5, *i*; compare also **Figure S6, *i***), while its formation is largely suppressed by the presence of TEMPO (**Figure S5, *ii*** and ***iii***). On the other hand, the latter two traces show peaks of residual starting materials, namely **1a** (peak at *ca*. 14.5 min.; compare also **Figure S6, *ii***) and TEMPO (peak at *ca*. 8.18 min.; compare also **Figure S6, *iii***).

i) ***0 equiv. TEMPO***

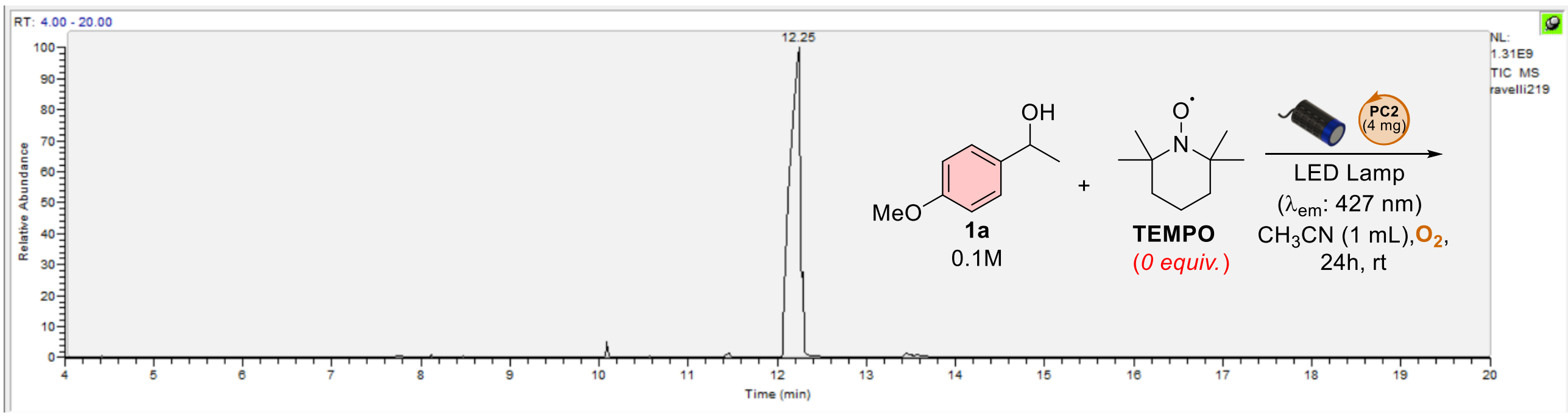

ii) ***0.5 equiv. TEMPO***

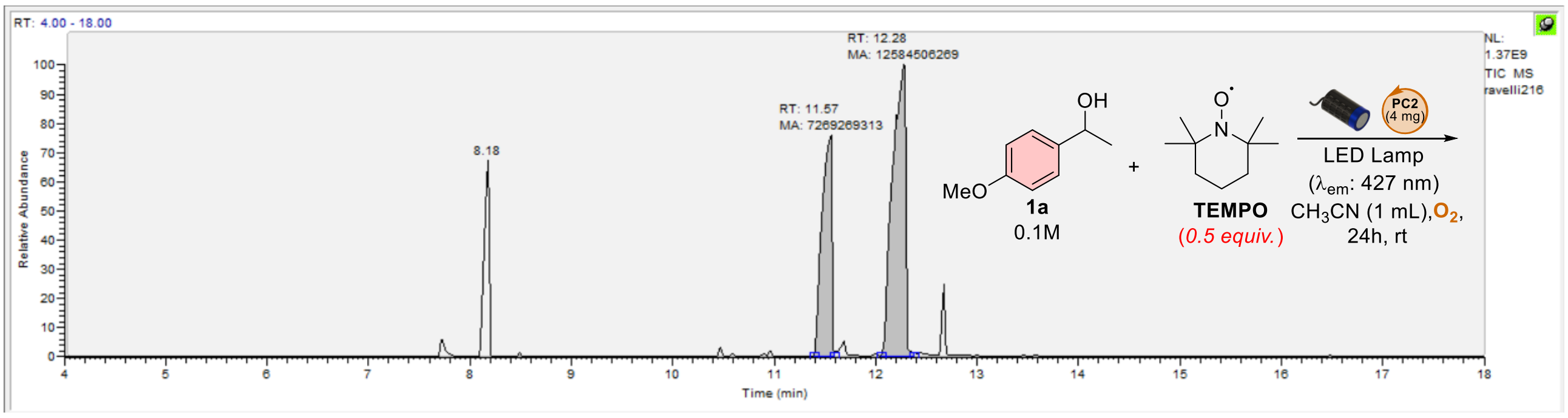

iii) ***1.5 equiv. TEMPO***

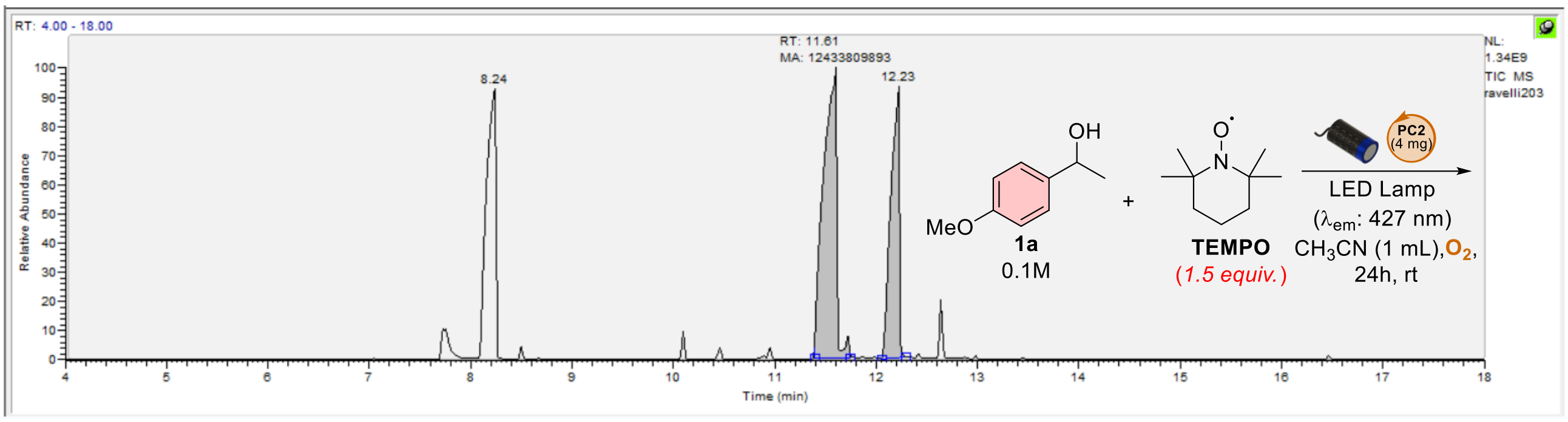

Figure S5. GC-MS traces of the crude reaction mixtures corresponding to the experiments on **1a** described in **Scheme S1**, in the presence of different amounts of the TEMPO additive: *i)* 0 equiv., *ii)* 0.5 equiv. and *iii)* 1.5 equiv.

i) ***Reference compound: 2***

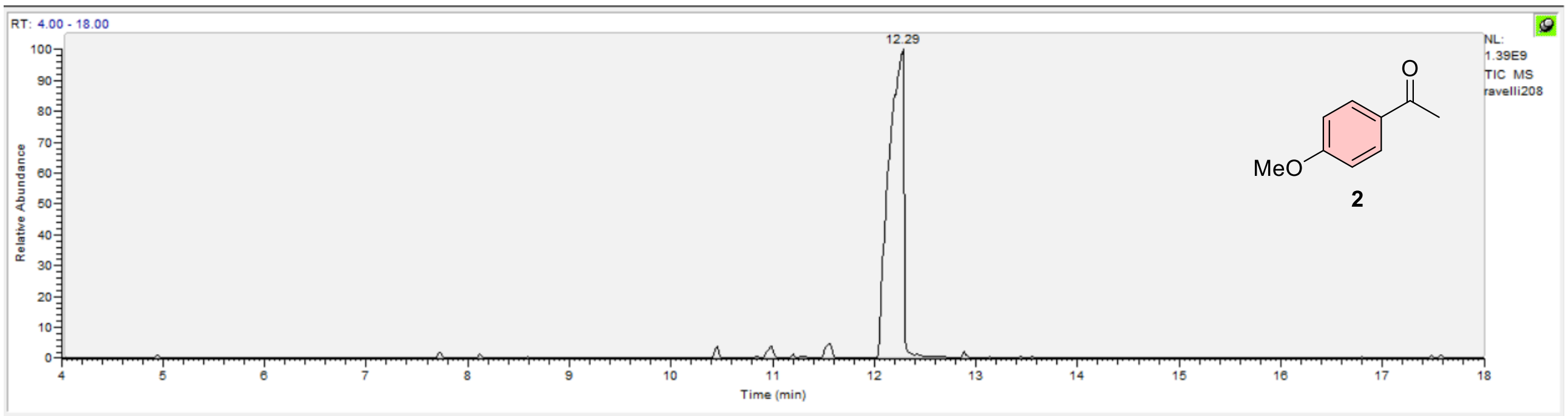

ii) ***Reference compound: 1a***

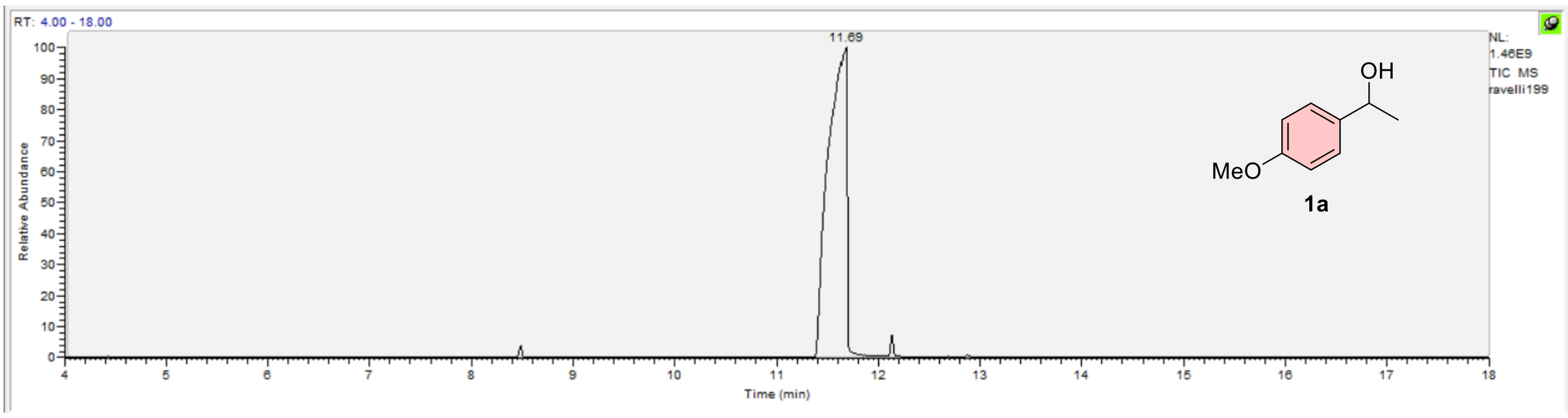

iii) ***Reference compound: TEMPO***

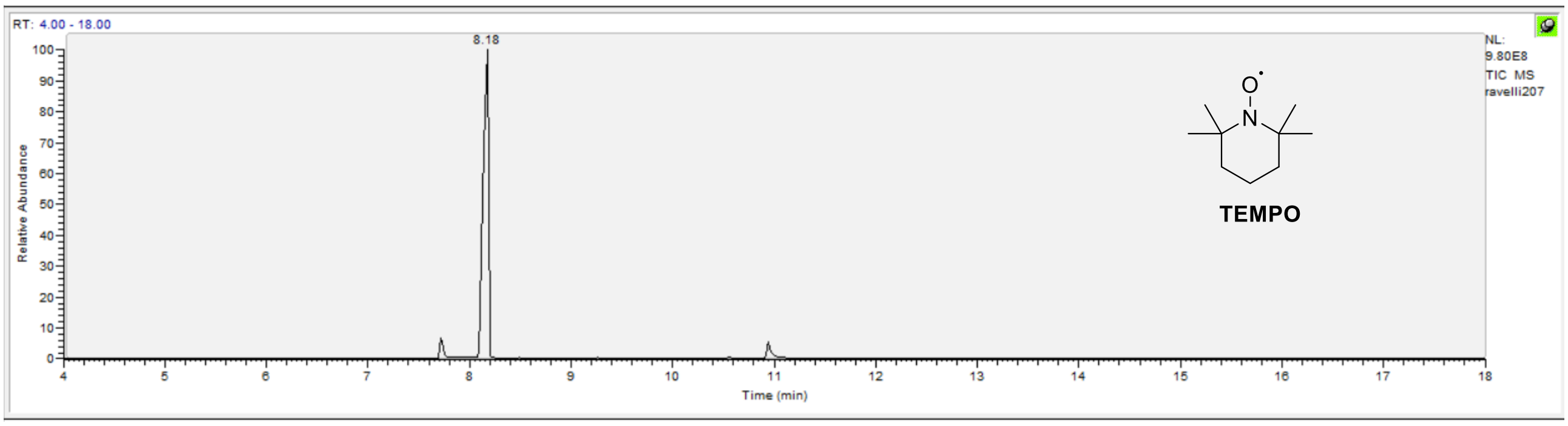

Figure S6. GC-MS traces of selected reference compounds: *i)* product **2**; *ii)* 1-(4-methoxyphenyl)ethan-1-ol (**1a**); *iii)* TEMPO additive.

Ascorbic acid (AA) as scavenger The model reaction involving 1-(4-methoxyphenyl)ethan-1-ol (**1a**; 0.1 mmol, 0.1 M) to prepare the oxidation product 1-(4-methoxyphenyl)ethan-1-one (**2**) was run in the presence of different amounts of the additive ascorbic acid (AA), a known superoxide radical anion ($O_2^{\bullet-}$) scavenger. The experiment was performed as indicated in **Scheme S2**, while the corresponding reaction crudes have been subjected to GC-MS analysis (**Figure S7**). The resulting GC traces were compared with those of selected reference compounds (**Figure S6**).

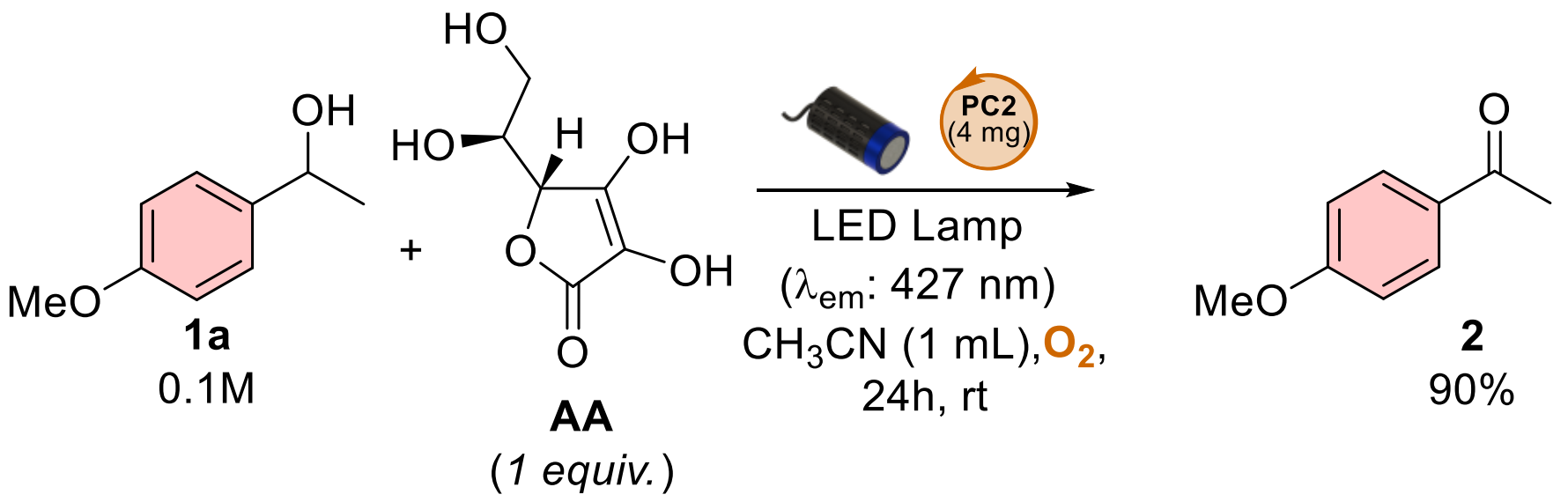

AA<br>*n* (equiv.)	2<br>Yield (%)
0	90%
1.0	90%

Scheme S2. Experiment performed to evaluate the effect of the ascorbic acid additive (AA) on the model reaction involving 1-(4-methoxyphenyl)ethan-1-ol (**1a**).

Upon inspection of the GC chart in **Figure S7**, it is apparent that the ascorbic acid additive doesn't impact on the reactivity, ruling out the involvement of the superoxide radical anion ($O_2^{\bullet-}$) and pointing to a direct interaction of the catalyst with the substrate **1a**. As apparent from **Scheme S2**, the formation of product **2** (peak at min. 12.29 in **Figure S6**, ***i***) is not suppressed in the presence of ascorbic acid, offering a reaction performance comparable to that observed with no additive present (**Figure S7, *i*** and ***ii***).

i) ***0 equiv. ascorbic acid***

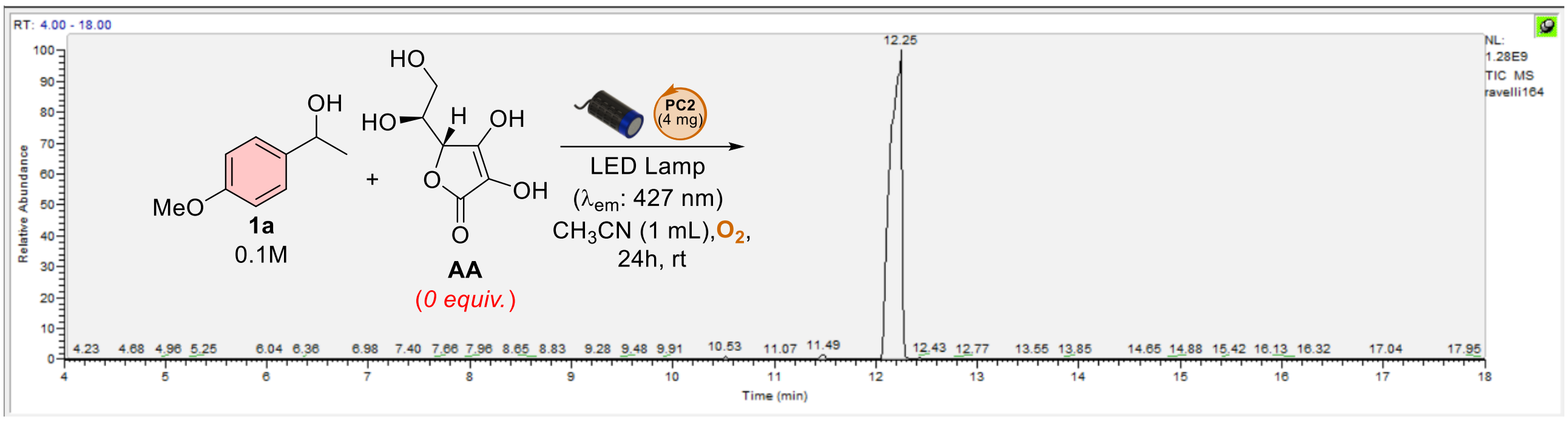

ii) ***1 equiv. ascorbic acid***

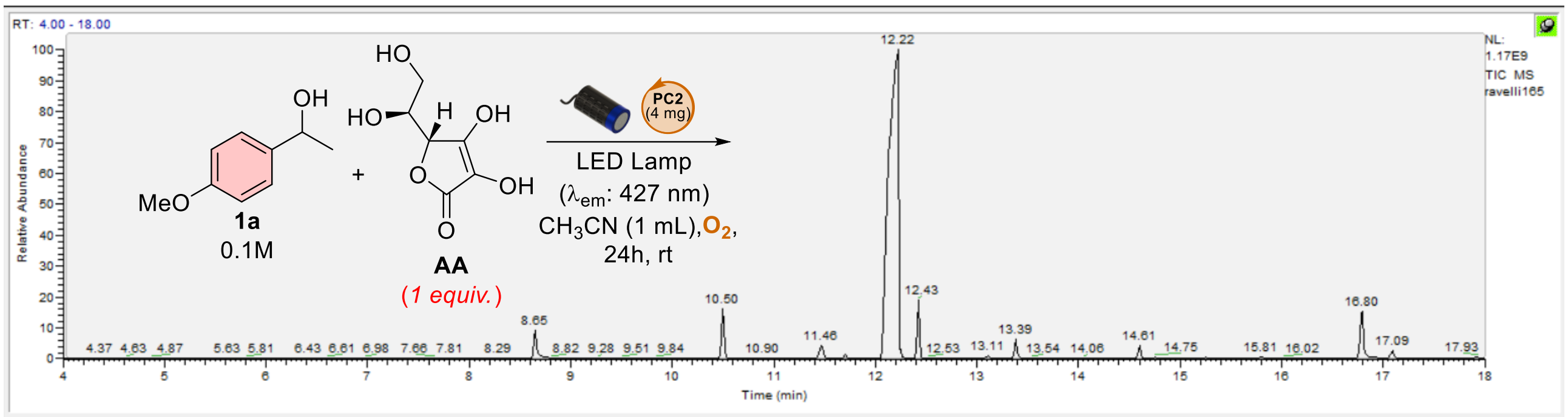

Figure S7. GC-MS trace of the crude reaction mixtures corresponding to the experiment on **1a** gathered in **Scheme S2**, run in the presence of the ascorbic acid additive (AA): *i)* 0 equiv.; *ii)* 1 equiv.

Ascorbic acid (AA) as scavenger The model reaction involving 1-(4-chlorophenyl)ethan-1-ol (**1d**; 0.1 mmol, 0.1 M) to prepare the oxidation product 1-(4-chlorophenyl)ethan-1-one (**5**) was run in the presence of different amounts of the additive ascorbic acid (AA), a known superoxide radical anion ($O_2^{\bullet-}$) scavenger. The experiment was performed as indicated in **Scheme S3**, while the corresponding reaction crudes have been subjected to GC-MS analysis (**Figure S8, *i-ii***). The resulting GC traces were compared with those of selected reference compounds (**Figure S8, *iii***).

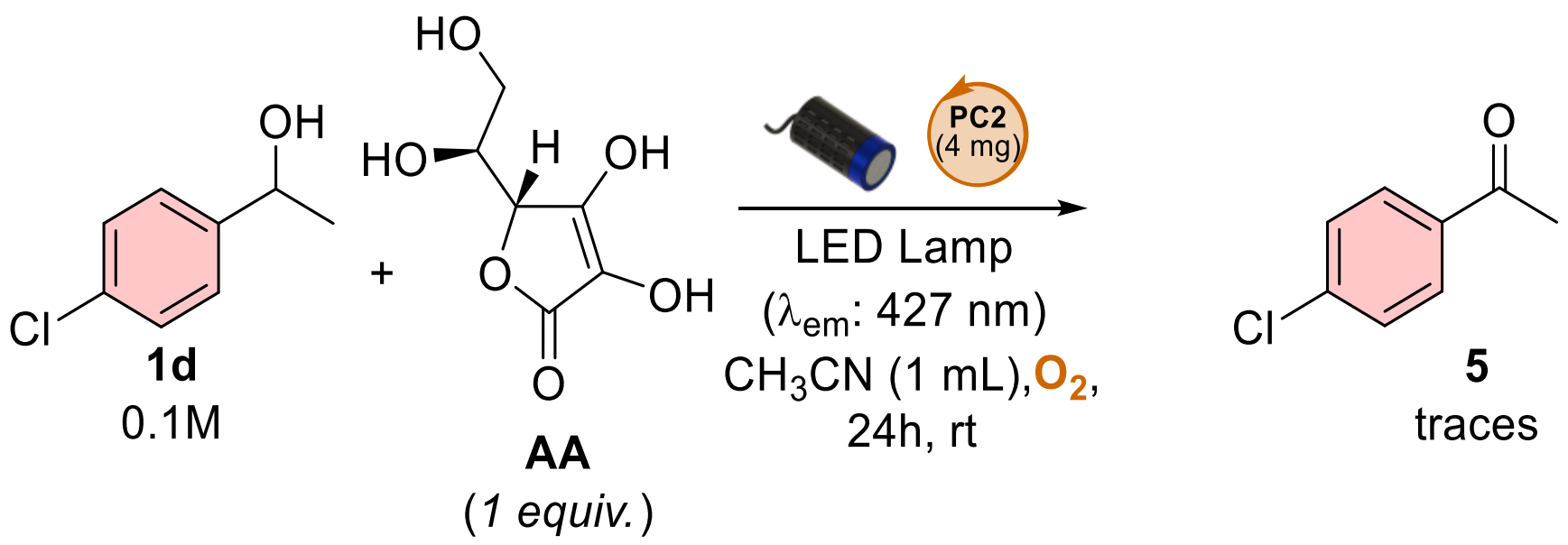

**AA** *n* (equiv.)	**5** Yield (%)
0	72%
1.0	traces

Scheme S3. Experiment performed to evaluate the effect of the ascorbic acid additive (AA) on the model reaction involving 1-(4-chlorophenyl)ethan-1-ol (**1d**).

Upon inspection of the GC chart in **Figure S8**, it is apparent the impact of the ascorbic acid additive, clearly demonstrating the role for the $O_2^{\bullet-}$ radical. As apparent from **Scheme S3**, the formation of product **5** (peak at min. 10.21 in **Figure S8**, ***iii***) is suppressed in the presence of ascorbic acid (**Figure S8, *ii***).

i) *0 equiv. ascorbic acid*

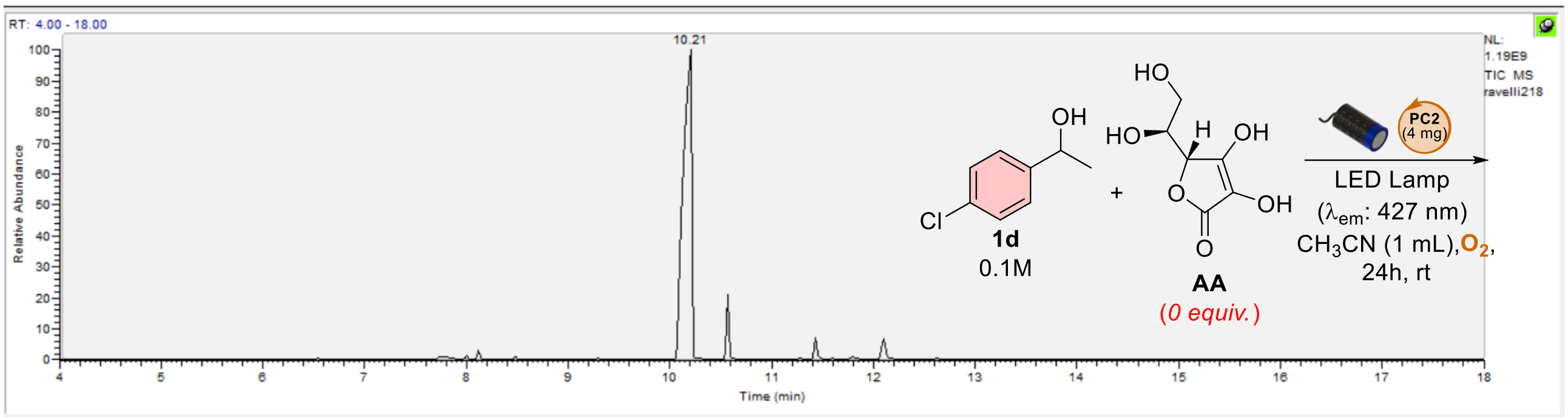

ii) *1 equiv. ascorbic acid*

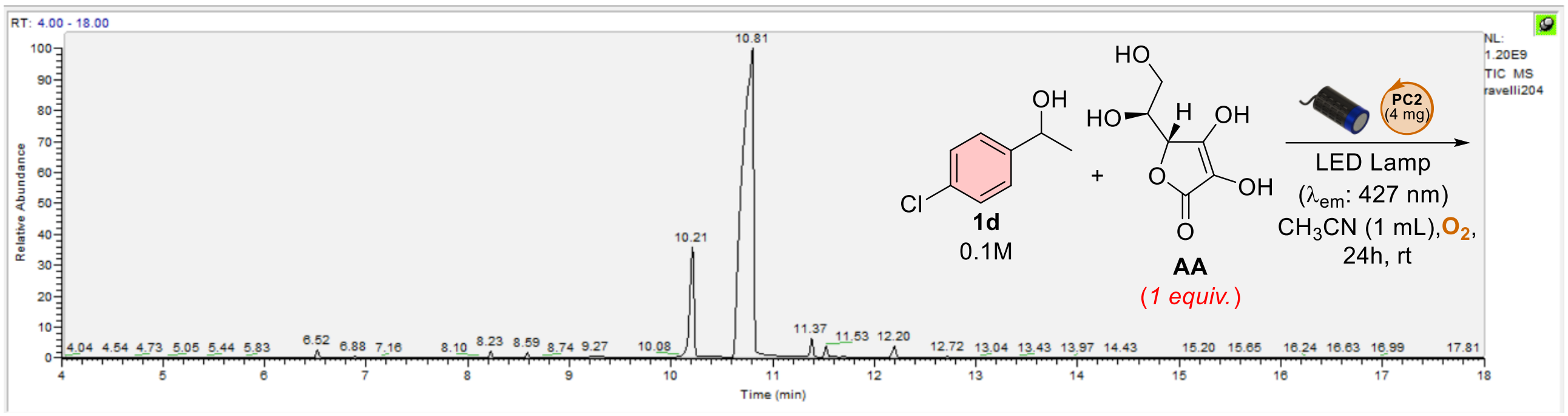

iii) *Reference compound: 5*

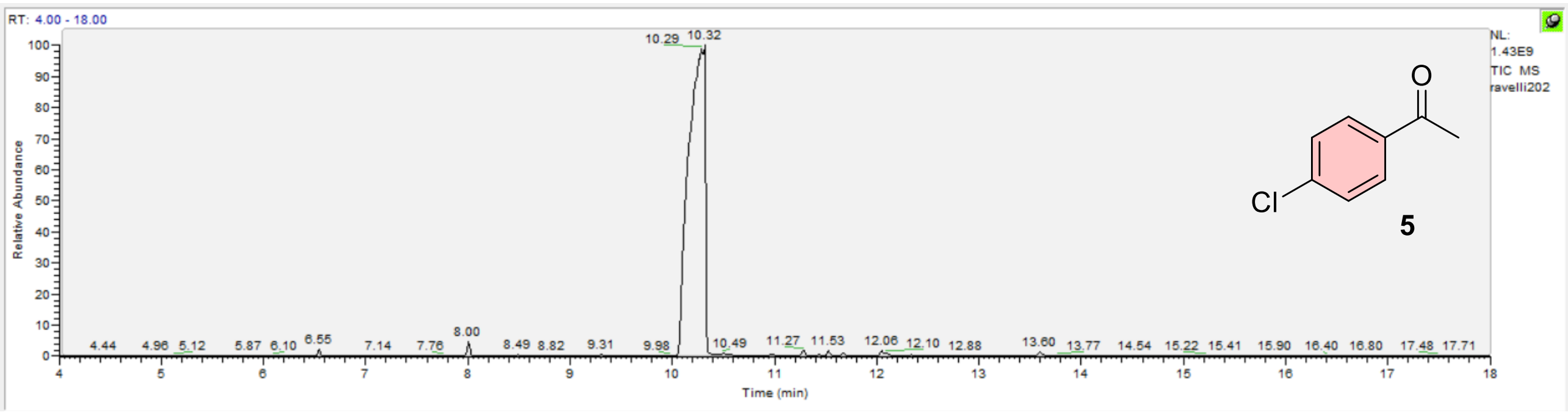

Figure S8. GC-MS trace of the crude reaction mixtures corresponding to the experiment on **1d** gathered in **Scheme S3**, run in the presence of the ascorbic acid additive (AA): *i)* 0 equiv.; *ii)* 1 equiv.; *iii)* reference compound: 1-(4-chlorophenyl)ethan-1-one (**5**).

2. Experimental details

2.1 General information

All reagents, chemicals and solvents used in this work were sourced from various commercial suppliers (TCI Europe, Merck - Sigma Aldrich, Fluorochem, Carlo Erba). Solvents and reagents were used as supplied, without any further purification. The *g*-C_3N_4/$CsPbCl_3$ materials used as photocatalyst were prepared according to procedure GPC (see **SECTION 2.2** below).

The light source adopted for photochemical experiments was a 40 W Kessil LED lamp (with emission centered at the specified wavelength; no filters used), operated at full power (technical specifications at https://www.kessil.com/science/PR160L) and positioned at *ca*. 10 cm from the reaction vessel.

Analytical thin layer chromatography (TLC) plates (silica gel 60 F254) were visualized either with a UV lamp (254 nm), or by submersion in the chosen stain for TLC. Column chromatography was carried out using 40-63 μm particle sized silica gel.

NMR spectra were recorded on a 400 (for ^{1}H) or 101 (for ^{13}C) MHz spectrometer; the attributions were made based on ^{1}H and ^{13}C NMR. Data for ^{1}H NMR are reported as follows: chemical shifts referred to TMS (δ ppm), multiplicity (s = singlet, bs = broad singlet, d = doublet, dd= double doublet, t = triplet, dt = double triplet, q = quadruplet, quint = quintuplet, m = multiplet), coupling constant (Hz) and integration. NMR spectra processing was performed by means of the MestreNova software.

GC/MS analyses were carried out on a Thermo Scientific DSQII single quadrupole GC/MS system. The injection in the GC/MS system was performed at 250°C in split mode. A Restek Rxi-5ms 30 m × 0.25 mm × 0.25 μm film thickness capillary column was used with helium as the carrier gas at a constant flow rate of 1.0 mL·min^{-1}. The transfer line temperature was held at 270°C and the ion source temperature was 250°C. Electron ionization mode was used with 70 eV and the ions were registered in full scan mode in a mass range of m/z 40-600 amu. The chromatogram acquisition, detection of mass spectral peaks and their waveform processing were performed using Xcalibur MS Software Version 2.2 (Thermo Scientific Inc.).

GC-FID analyses were performed on an Agilent 7820A chromatograph. The injection was performed at 250 °C in split mode. The initial oven temperature of 80 °C was maintained for 2 min, increased by 10 °C/min to 250 °C and held for 5 min. An Agilent HP5 30 m × 0.32 mm × 0.25 μm film thickness capillary column was used with nitrogen as the carrier gas at a constant flow rate of 6.0 mL·min^{-1}. GC yields were calculated using 1,3,5-trimethoxybenzene as internal standard.

A single quadrupole inductively coupled plasma mass spectrometer (SQ-ICP-MS, iCAP RQ Thermo FisherScientific), equipped with a quartz cyclonic chamber cooled at 3 °C, a MicroMist nebulizer 179 (400µL/min), a quartz torch, a Ni sampler, skimmer cones, and a QCell pressurized with helium (3V, KED mode), was used for ICP analysis.

For catalyst recovery purposes, an OHAUS Frontier™ Multi Pro FC5816 centrifuge with 6×50 mL rotor has been adopted.

Photoluminescence (PL) measurements were conducted using a Horiba Jobin Yvon Nanolog 3-11 spectrofluorometer equipped with a photomultiplier tube (PMT) detector. Powdered samples were placed between two quartz substrates. Emission spectra were collected in the range of 400–630 nm under 375 nm excitation, with entrance/exit slit widths set to 5 nm and an integration time of 0.1 s.

2.2 General procedure for the synthesis of the catalyst (GPC)

$CsPbCl_3$ powders were synthesized by mechanochemical ball milling. Stoichiometric amounts of $PbCl_2$ and CsCl (both Aldrich, 99.99%) were loaded into tungsten carbide milling jars together with tungsten carbide balls, using a ball-to-powder weight ratio of 10:1. The mixtures were milled at 300 rpm for seven cycles of 30 min. each, consisting of 20 min of effective milling followed by a 10 min. pause to prevent overheating.

Bulk *g*-C_3N_4 was first synthesized by the thermal polymerization of dicyandiamide (DCD). DCD was placed in an alumina crucible inside a quartz tube furnace under a continuous nitrogen flow. The sample was heated to 550 °C at a rate of 4 °C·min^{-1}, maintained at this temperature for 4 h, and then cooled to room temperature with a rate of 6 °C·min^{-1}. The obtained bulk *g*-C_3N_4 was ground into a fine powder and subsequently exfoliated by thermal treatment in air. Briefly, 1 g of bulk *g*-C_3N_4 was heated in a tubular furnace to 545 °C at a heating rate of 4 °C·min^{-1}, held at this temperature for 2 h, and finally cooled to room temperature with a rate of 6 °C·min^{-1}, yielding exfoliated *g*-C_3N_4 nanosheets, according to previously published procedure.[3]

The *g*-C_3N_4/$CsPbCl_3$ heterojunctions were prepared with the same procedure adopted for analogous systems in the past by our group.[1] The following weight ratios between nano exfoliated *g*-C_3N_4 and $CsPbCl_3$ have been investigated: 0, 10, 30, 50, 90 and 100 (these values do refer to the $\%_{W/W}$ of perovskite in the material).

3. Photocatalyzed aerobic oxidation reactions

3.1 General procedures for photocatalyzed aerobic oxidation reactions

3.1.1 Typical procedure for small scale reactions (GP-*a*)

In a Pyrex glass reaction tube equipped with a magnetic microstir bar, the chosen benzylic alcohol (**1**, 0.1 mmol, 0.1 M) was dissolved in the chosen solvent mixture (MeCN, 1 mL) in the presence of 4 mg of the *g*-C_3N_4/$CsPbCl_3$ catalyst (90:10$_{W/W}$, **PC2**). After bubbling O_2 for 1 minute, the tube was capped, and the resulting mixture was then placed in a home-made photoreactor inspired by the design reported by Leonori[4] and equipped with a side fan and irradiated for 24h with a Kessil lamp (λ_{em} = 427 nm, 40W; see **Figure S9**) at room temperature (*ca*. 20/25 °C). After solvent removal under vacuum, the amount of product formed and residual unreacted starting material (if any) was monitored via GC analysis using 1,3,5-trimethoxybenzene as internal standard (0.1 mmol, 0.1 M).

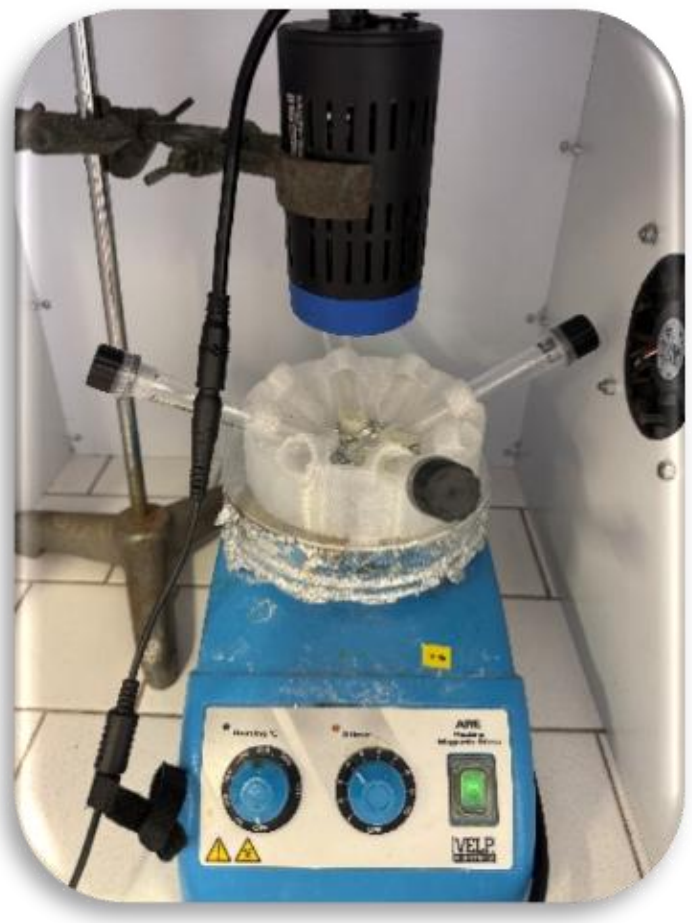

Figure S9. Pyrex glass irradiation tubes containing the reaction mixture in the experimental apparatus used for small scale experiments, consisting of a home-made photoreactor equipped with a Kessil Lamp (λ_{em} = 427 nm, 40W).

3.1.2 Typical procedure for preparative reactions (GP-*b*)

In a Pyrex glass reaction tube equipped with a magnetic stir bar, the chosen benzylic alcohol (**1**; 0.5 mmol, 0.1 M) was dissolved in acetonitrile (5 mL) in the presence of 20 mg of the *g*-C_3N_4/$CsPbCl_3$ catalyst (90:10$_{W/W}$, **PC2**). After bubbling O_2 for 5 minutes, the tube was capped, and the resulting mixture was irradiated at room temperature (*ca*. 20/25°C) for 24h in a box equipped with a side fan

with a Kessil lamp (λ_{em} = 427 nm, 40W; see **Figure S10**) kept at *ca*. 10 cm from the reaction mixture. After solvent removal under vacuum, the crude mixture was purified by column chromatography (silica gel; cyclohexane/ethyl acetate mixture as eluant) to afford the pure product.

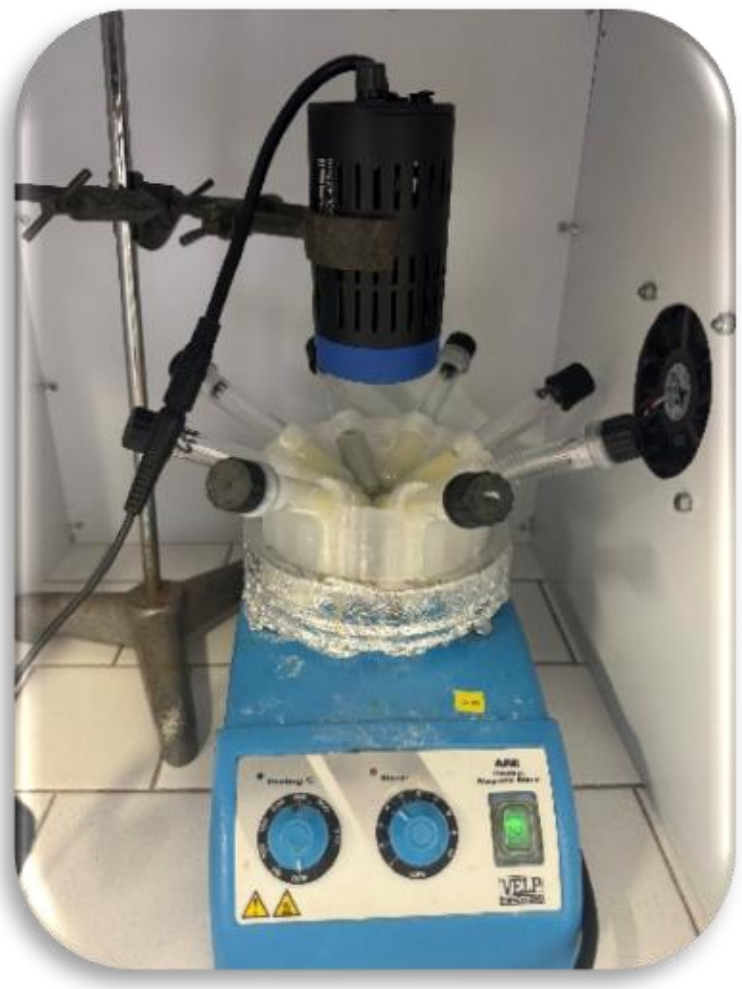

Figure S10. Typical reaction setup adopted for preparative experiments, consisting of a Pyrex glass reaction tube containing the reaction mixture and equipped with a magnetic stir bar, irradiated from the top with a Kessil Lamp (λ_{em} = 427 nm, 40W) kept at *ca*. 10 cm from the glass vessel in a fan-cooled box.

3.1.3 Typical procedure for gram scale reactions (GP-*c*)

In a Pyrex round bottom flask equipped with a magnetic stir bar, the chosen benzylic alcohol (**1**, 10 mmol, 0.1 M) was dissolved in acetonitrile (100 mL) in the presence of 400 mg of the *g*-C_3N_4/$CsPbCl_3$ catalyst (90:10$_{W/W}$, **PC2**). After bubbling O_2 for 15 minutes, the glass vessel was capped, and the resulting mixture was irradiated at room temperature (*ca*. 20/25°C) in a box equipped with a side fan with a Kessil lamp (λ_{em} = 427 nm, 40W; see **Figure S11**) kept at *ca*. 10 cm from the flask. The irradiation was monitored by TLC until completion (generally, 48h); after solvent removal under vacuum, the crude mixture was purified by column chromatography (silica gel; cyclohexane/ethyl acetate mixture as eluant) to afford the pure product.

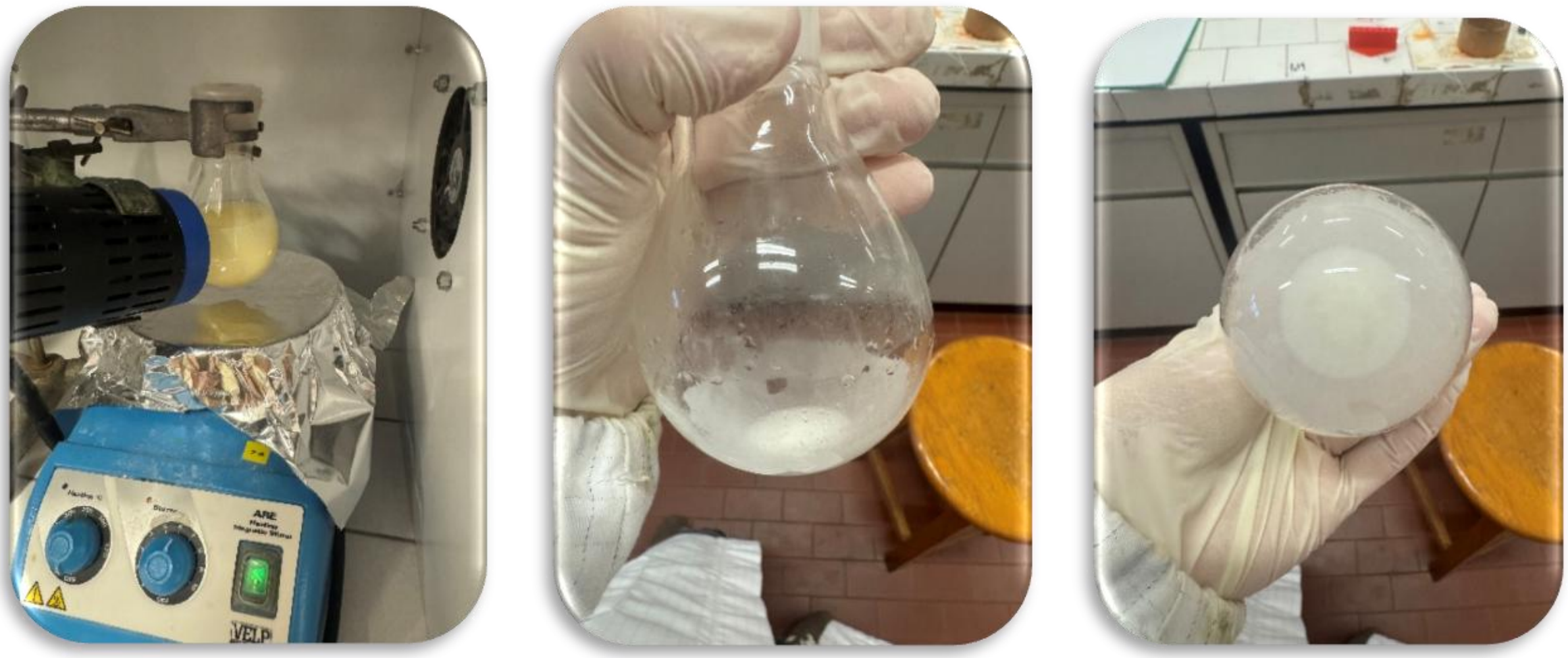

Figure S11. Left part: experimental setup adopted for gram-scale reactions, consisting of a Pyrex round bottom flask containing the solution mixture and equipped with a magnetic stir bar, irradiated with a Kessil Lamp (λ_{em} = 427 nm, 40W) kept at *ca*. 10 cm from the flask in a fan-cooled box. Center & right part: Pyrex round bottom flask with the obtained purified product.

3.2 Characterization data for products 2-26

1-(4-Methoxyphenyl)ethan-1-one (2)

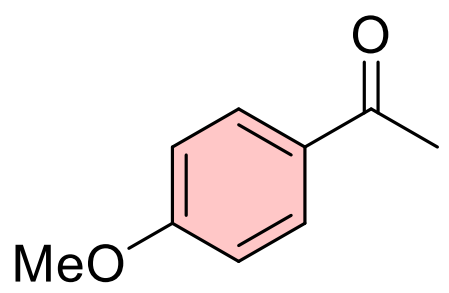

Following **GP-*b***, starting from 70 µL (0.5 mmol, 0.1M) of 1-(4-methoxyphenyl)ethan-1-ol (**1a**) and 20 mg of *g*-$C_3N_4/CsPbCl_3$ (90%/10%) catalyst in 5 mL of acetonitrile. The crude mixture was purified using silica gel chromatography (95:5 cyclohexane/ethyl acetate) to afford 67 mg of **2** (0.44 mmol, 88% yield) as a white solid (m.p.: 37-38°C; Lit.:[5] 36-38°C). Spectroscopic data for **2** are in accordance with literature.[5]

Following **GP-*c***, starting from 1.4 mL (10.0 mmol, 0.1M) of 1-(4-methoxyphenyl)ethan-1-ol (**1a**) and 400 mg of *g*-$C_3N_4/CsPbCl_3$ (90%/10%) catalyst in 100 mL of acetonitrile. The crude mixture was purified using silica gel chromatography (95:5 cyclohexane/ethyl acetate) to afford 1100 mg of **2** (7.3 mmol, 73% yield).

^{1}H NMR (400 MHz, $CDCl_3$) δ 7.96 – 7.88 (m, 2H), 6.98 – 6.88 (m, 2H), 3.85 (s, 3H), 2.54 (s, 3H).

$^{13}C\{^1H\}$ NMR (101 MHz, $CDCl_3$) δ 196.9, 163.6, 130.7, 130.5, 113.8, 55.6, 26.4.

1-(*p*-Tolyl)ethan-1-one (3)

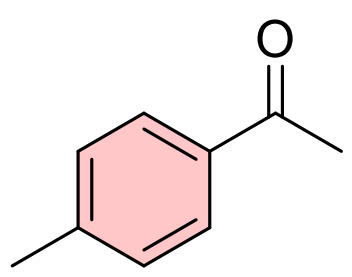

Following **GP-*b***, starting from 69 µL (0.5 mmol, 0.1M) of 1-(*p*-tolyl)ethan-1-ol (**1b**) and 20 mg of *g*-$C_3N_4/CsPbCl_3$ (90%/10%) catalyst in 5 mL of acetonitrile. The crude mixture was purified using silica gel chromatography (95:5 cyclohexane/ethyl acetate) to afford 20 mg of **3** (0.15 mmol, 30% yield) as a colorless oil. Spectroscopic data for **3** are in accordance with literature.[5]

^{1}H NMR (400 MHz, $CDCl_3$) δ 7.77 – 7.69 (m, 2H), 7.17 – 7.09 (m, 2H), 2.45 (s, 3H), 2.28 (s, 3H).

$^{13}C\{^1H\}$ NMR (101 MHz, $CDCl_3$) δ 198.0, 144.0, 134.9, 129.4, 128.6, 26.6, 21.7.

1-(4-Bromophenyl)ethan-1-one (4)

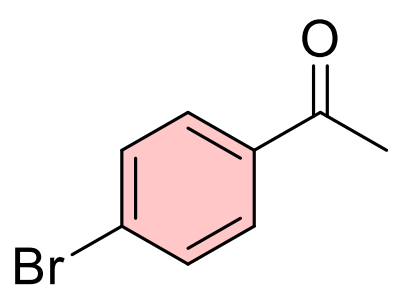

Following **GP-*b***, starting from 77 µL (0.5 mmol, 0.1M) of 1-(4-bromophenyl)ethan-1-ol (**1c**) and 20 mg of *g*-$C_3N_4/CsPbCl_3$ (90%/10%) catalyst in 5 mL of acetonitrile. The crude mixture was purified using silica gel chromatography (95:5

cyclohexane/ethyl acetate) to afford 45 mg of **4** (0.23 mmol, 46% yield) as a white solid (m.p.: 49-51°C; Lit.:[5] 48-52°C). Spectroscopic data for **4** are in accordance with literature.[5]

^{1}H NMR (400 MHz, CDCl$_3$) δ 7.84 – 7.76 (m, 2H), 7.63 – 7.54 (m, 2H), 2.56 (s, 3H).

^{13}C{^{1}H} NMR (101 MHz, CDCl$_3$) δ 197.1, 135.9, 132.0, 129.9, 128.4, 26.6.

1-(4-Chlorophenyl)ethan-1-one (5)

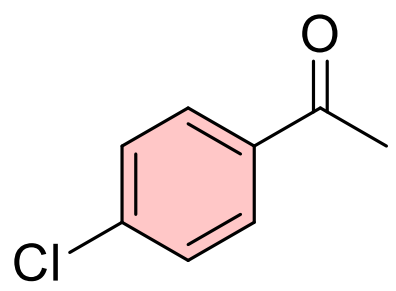

Following **GP-*b***, starting from 67 µL (0.5 mmol, 0.1M) of 1-(4-chlorophenyl)ethan-1-ol (**1d**) and 20 mg of *g*-C_3N_4/$CsPbCl_3$ (90%/10%) catalyst in 5 mL of acetonitrile. The crude mixture was purified using silica gel chromatography (95:5 cyclohexane/ethyl acetate) to afford 51 mg of **5** (0.33 mmol, 66% yield) as a pale yellow oil. Spectroscopic data for **5** are in accordance with literature.[5]

^{1}H NMR (400 MHz, CDCl$_3$) δ 7.93 – 7.84 (m, 2H), 7.47 – 7.38 (m, 2H), 2.58 (s, 3H).

^{13}C{^{1}H} NMR (101 MHz, CDCl$_3$) δ 196.9, 139.7, 135.6, 129.9, 129.0, 26.7.

1-(3-Methoxyphenyl)ethan-1-one (7)

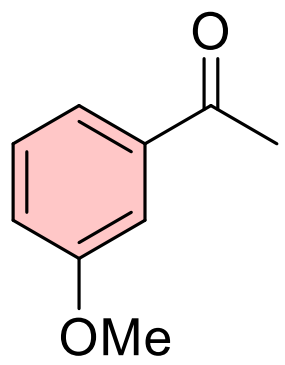

Following **GP-*b***, starting from 76 µL (0.5 mmol, 0.1M) of 1-(3-methoxyphenyl)ethan-1-ol (**1f**) and 20 mg of *g*-C_3N_4/$CsPbCl_3$ (90%/10%) catalyst in 5 mL of acetonitrile. The crude mixture was purified using silica gel chromatography (95:5 cyclohexane/ethyl acetate) to afford 60 mg of **7** (0.40 mmol, 80% yield) as a colorless oil. Spectroscopic data for **7** are in accordance with literature.[6]

^{1}H NMR (400 MHz, CDCl$_3$) δ 7.53 (ddd, J = 7.6, 1.6, 1.0 Hz, 1H), 7.48 (dd, J = 2.7, 1.5 Hz, 1H), 7.36 (t, J = 7.9 Hz, 1H), 7.10 (ddd, J = 8.2, 2.7, 1.0 Hz, 1H), 3.85 (s, 3H), 2.59 (s, 3H).

^{13}C{^{1}H} NMR (101 MHz, CDCl$_3$) δ 198.1, 159.9, 138.6, 129.7, 121.2, 119.7, 112.5, 55.5, 26.8.

1-(*m*-Tolyl)ethan-1-one (8)

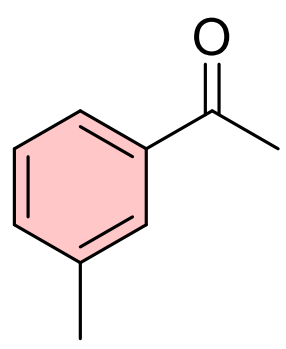

Following **GP-*b***, starting from 62 µL (0.5 mmol, 0.1M) of 1-(*m*-tolyl)ethan-1-ol (**1g**) and 20 mg of *g*-C_3N_4/$CsPbCl_3$ (90%/10%) catalyst in 5 mL of acetonitrile. The crude mixture was purified using silica gel chromatography (95:5 cyclohexane/ethyl acetate)

to afford 29 mg of **8** (0.22 mmol, 44% yield) as a yellowish oil. Spectroscopic data for **8** are in accordance with literature.[7]

^{1}H NMR (400 MHz, CDCl$_3$) δ 7.80 – 7.72 (m, 2H), 7.42 – 7.29 (m, 2H), 2.59 (s, 3H), 2.41 (s, 3H).

^{13}C{^{1}H} NMR (101 MHz, CDCl$_3$) δ 198.6, 138.5, 137.3, 134.0, 128.9, 128.6, 125.7, 26.8, 21.5.

1-(3-Bromophenyl)ethan-1-one (9)

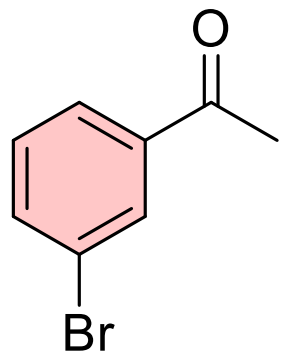

Following **GP-*b***, starting from 68 μL (0.5 mmol, 0.1M) of 1-(3-bromophenyl)ethan-1-ol (**1h**) and 20 mg of *g*-C_3N_4/$CsPbCl_3$ (90%/10%) catalyst in 5 mL of acetonitrile. The crude mixture was purified using silica gel chromatography (95:5 cyclohexane/ethyl acetate) to afford 91 mg of **9** (0.46 mmol, 92% yield) as a colorless oil. Spectroscopic data for **9** are in accordance with literature.[5]

^{1}H NMR (400 MHz, CDCl$_3$) δ 8.07 (t, *J* = 1.8 Hz, 1H), 7.86 (ddd, *J* = 7.8, 1.7, 1.1 Hz, 1H), 7.67 (ddd, *J* = 7.9, 2.0, 1.0 Hz, 1H), 7.33 (t, *J* = 7.9 Hz, 1H), 2.58 (s, 3H).

^{13}C{^{1}H} NMR (101 MHz, CDCl$_3$) δ 196.7, 138.9, 136.0, 131.5, 130.3, 127.0, 123.1, 26.7.

1-(3-Chlorophenyl)ethan-1-one (10)

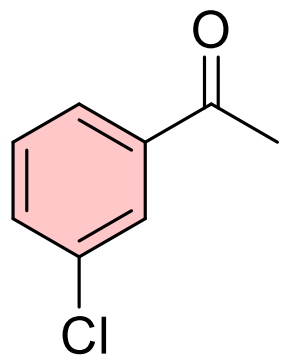

Following **GP-*b***, starting from 67 μL (0.5 mmol, 0.1M) of 1-(3-chlorophenyl)ethan-1-ol (**1i**) and 20 mg of *g*-C_3N_4/$CsPbCl_3$ (90%/10%) catalyst in 5 mL of acetonitrile. The crude mixture was purified using silica gel chromatography (95:5 cyclohexane/ethyl acetate) to afford 34 mg of **10** (0.22 mmol, 44% yield) as a yellowish oil. Spectroscopic data for **10** are in accordance with literature.[8]

^{1}H NMR (400 MHz, CDCl$_3$) δ 7.93 (t, *J* = 1.9 Hz, 1H), 7.83 (ddd, *J* = 7.8, 1.6, 1.1 Hz, 1H), 7.54 (ddd, *J* = 8.0, 2.1, 1.1 Hz, 1H), 7.41 (t, *J* = 7.9 Hz, 1H), 2.60 (s, 3H).

^{13}C{^{1}H} NMR (101 MHz, CDCl$_3$) δ 196.9, 138.8, 135.1, 133.2, 130.1, 128.6, 126.5, 26.8.

1-(2-Methoxyphenyl)ethan-1-one (11)

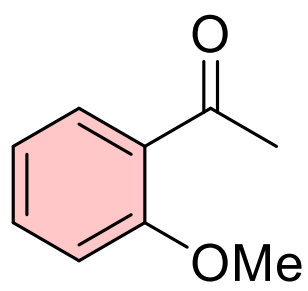

Following **GP-*b***, starting from 76 mg (0.5 mmol, 0.1M) of 1-(2-methoxyphenyl)ethan-1-ol (**1j**) and 20 mg of *g*-C_3N_4/$CsPbCl_3$ (90%/10%) catalyst in 5 mL of acetonitrile. The crude mixture was purified using silica gel chromatography (95:5

cyclohexane/ethyl acetate) to afford 34 mg of **11** (0.23 mmol, 46% yield) as a colorless oil. Spectroscopic data for **11** are in accordance with literature.[5]

^{1}H NMR (400 MHz, CDCl$_3$) δ 7.73 (dd, *J* = 7.7, 1.8 Hz, 1H), 7.46 (ddd, *J* = 8.3, 7.3, 1.8 Hz, 1H), 7.05 – 6.92 (m, 2H), 3.91 (s, 3H), 2.61 (s, 3H).

^{13}C{^{1}H} NMR (101 MHz, CDCl$_3$) δ 200.0, 159.0, 133.8, 130.5, 128.4, 120.7, 111.7, 55.6, 31.9.

1-(2-Bromophenyl)ethan-1-one (13)

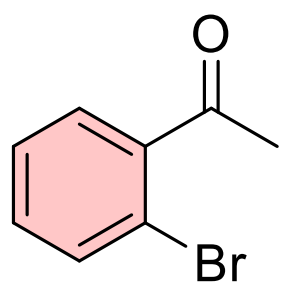

Following **GP-*b***, starting from 68 µL (0.5 mmol, 0.1M) of 1-(2-bromophenyl)ethan-1-ol (**1l**) and 20 mg of *g*-C_3N_4/$CsPbCl_3$ (90%/10%) catalyst in 5 mL of acetonitrile. The crude mixture was purified using silica gel chromatography (95:5 cyclohexane/ethyl acetate) to afford 42 mg of **13** (0.21 mmol, 42% yield) as a white solid (m.p.: 49-51 °C; Lit.:[9] 48.5-49.4°C). Spectroscopic data for **13** are in accordance with literature.[10]

^{1}H NMR (400 MHz, CDCl$_3$) δ 7.42 (dd, *J* = 8.0, 1.2 Hz, 1H), 7.27 (dd, *J* = 7.6, 1.8 Hz, 1H), 7.17 (td, *J* = 7.5, 1.2 Hz, 1H), 7.14 – 7.06 (m, 1H), 2.44 (s, 3H).

^{13}C {^{1}H} NMR (101 MHz, CDCl$_3$) δ 201.1, 141.2, 133.6, 131.6, 128.7, 127.2, 118.6, 30.1.

1-(2-Chlorophenyl)ethan-1-one (14)

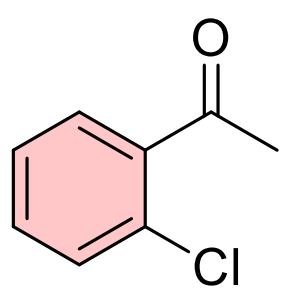

Following **GP-*b***, starting from 67 µL (0.5 mmol, 0.1M) of 1-(2-chlorophenyl)ethan-1-ol (**1m**) and 20 mg of *g*-C_3N_4/$CsPbCl_3$ (90%/10%) catalyst in 5 mL of acetonitrile. The crude mixture was purified using silica gel chromatography (95:5 cyclohexane/ethyl acetate) to afford 20 mg of **14** (0.13 mmol, 26% yield) as a colorless dense oil. Spectroscopic data for **14** are in accordance with literature.[7]

^{1}H NMR (400 MHz, CDCl$_3$) δ 7.56 (dd, *J* = 1.7, 0.6 Hz, 1H), 7.45 – 7.36 (m, 2H), 7.32 (ddd, *J* = 7.5, 6.9, 1.8 Hz, 1H), 2.65 (s, 3H).

^{13}C{^{1}H} NMR (101 MHz, CDCl$_3$) δ 200.6, 139.3, 132.1, 131.5, 130.8, 129.6, 127.1, 30.9.

Acetophenone (15)

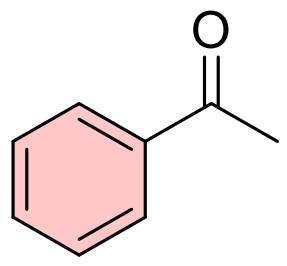

Following **GP-*b***, starting from 60 μL (0.5 mmol, 0.1M) of 1-phenylethan-1-ol (**1n**) and 20 mg of *g*-C_3N_4/$CsPbCl_3$ (90%/10%) catalyst in 5 mL of acetonitrile. The crude mixture was purified using silica gel chromatography (95:5 cyclohexane/ethyl acetate) to afford 27 mg of **15** (0.23 mmol, 46% yield) as a pale yellow oil. Spectroscopic data for **15** are in accordance with literature.[5]

^{1}H NMR (400 MHz, $CDCl_3$) δ 8.01 – 7.92 (m, 2H), 7.61 – 7.52 (m, 1H), 7.52 – 7.42 (m, 2H), 2.61 (s, 3H).

^{13}C{^{1}H} NMR (101 MHz, $CDCl_3$) δ 198.3, 137.3, 133.2, 128.7, 128.4, 26.7.

2,3-dihydro-1H-inden-1-one (17)

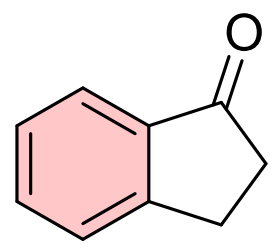

Following **GP-*b***, starting from 67 mg (0.5 mmol, 0.1M) of 2,3-dihydro-1H-inden-1-ol (**1p**) and 20 mg of *g*-C_3N_4/$CsPbCl_3$ (90%/10%) catalyst in 5 mL of acetonitrile. The crude mixture was purified using silica gel chromatography (95:5 cyclohexane/ethyl acetate) to afford 30 mg of **17** (0.23 mmol, 46% yield) as a yellowish solid (m.p.: 41-43°C; Lit.:[11] 40.4-41.9°C). Spectroscopic data for **17** are in accordance with literature.[6]

^{1}H NMR (400 MHz, $CDCl_3$) δ 7.75 (d, *J* = 7.7 Hz, 1H), 7.58 (td, *J* = 7.4, 1.2 Hz, 1H), 7.47 (dt, *J* = 7.8, 0.9 Hz, 1H), 7.40 – 7.32 (m, 1H), 3.17 – 3.10 (m, 2H), 2.74 – 2.64 (m, 2H).

^{13}C{^{1}H} NMR (101 MHz, $CDCl_3$) δ 207.2, 155.3, 137.2, 134.7, 127.4, 126.8, 123.8, 36.3, 25.9.

3,4-Dihydronaphthalen-1(2H)-one (18)

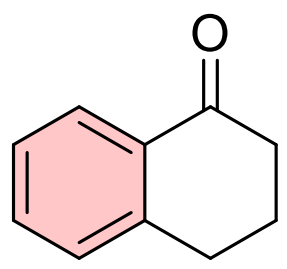

Following **GP-*b***, starting from 68 μL (0.5 mmol, 0.1M) of 1,2,3,4-tetrahydronaphthalen-1-ol (**1q**) and 20 mg of *g*-C_3N_4/$CsPbCl_3$ (90%/10%) catalyst in 5 mL of acetonitrile. The crude mixture was purified using silica gel chromatography (95:5 cyclohexane/ethyl acetate) to afford 52 mg of **18** (0.37 mmol, 74% yield) as a colorless oil. Spectroscopic data for **18** are in accordance with literature.[5]

^{1}H NMR (400 MHz, $CDCl_3$) δ 8.32 (dd, *J* = 7.8, 1.5 Hz, 1H), 7.75 (td, *J* = 7.5, 1.5 Hz, 1H), 7.63 – 7.50 (m, 2H), 3.25 (t, *J* = 6.1 Hz, 2H), 2.94 (dd, *J* = 7.3, 5.8 Hz, 2H), 2.48 – 2.37 (m, 2H).

^{13}C{^{1}H} NMR (101 MHz, $CDCl_3$) δ 198.5, 144.6, 133.5, 132.7, 128.9, 127.3, 126.7, 39.3, 29.8, 23.4.

4-Methoxybenzaldehyde (19)

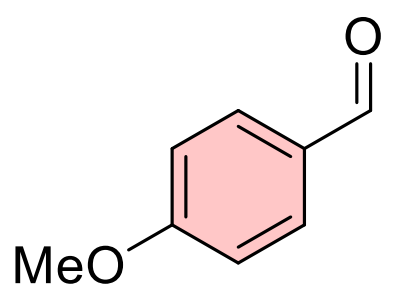

Following **GP-*b***, starting from 63 μL (0.5 mmol, 0.1M) of (4-methoxyphenyl)methanol (**1r**) and 20 mg of *g*-C_3N_4/$CsPbCl_3$ (90%/10%) catalyst in 5 mL of acetonitrile. The crude mixture was purified using silica gel chromatography (95:5 cyclohexane/ethyl acetate) to afford 46 mg of **19** (0.34 mmol, 68% yield) as a yellowish oil. Spectroscopic data for **19** are in accordance with literature.[12] If the irradiation was prolonged up to 40h, compound **19** was consistently observed as the only product (0.32 mmol, 64% yield).

Following **GP-*c***, starting from 1.4 mL (10.0 mmol, 0.1M) of 1-(4-methoxyphenyl)ethan-1-ol (**1a**) and 400 mg of g-C_3N_4/$CsPbCl_3$ (90%/10%) catalyst in 100 mL of acetonitrile. The crude mixture was purified using silica gel chromatography (95:5 cyclohexane/ethyl acetate) to afford 700 mg of **2** (7.3 mmol, 73% yield).

^{1}H NMR (400 MHz, $CDCl_3$) δ 9.88 (s, 1H), 7.87 – 7.79 (m, 2H), 7.04 – 6.96 (m, 2H), 3.88 (d, *J* = 0.8 Hz, 3H).

^{13}C {^{1}H} NMR (101 MHz, $CDCl_3$) δ 190.9, 164.7, 132.1, 130.1, 114.4, 55.7.

4-Methylbenzaldehyde (20)

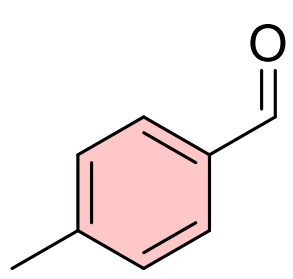

Following **GP-*b***, starting from 61 mg (0.5 mmol, 0.1M) of p-tolylmethanol (**1s**) and 20 mg of *g*-C_3N_4/$CsPbCl_3$ (90%/10%) catalyst in 5 mL of acetonitrile. The crude mixture was purified using silica gel chromatography (95:5 cyclohexane/ethyl acetate) to afford 19 mg of **20** (0.16 mmol, 32% yield) as a colorless oil. Spectroscopic data for **20** are in accordance with literature.[13]

^{1}H NMR (400 MHz, $CDCl_3$) δ 9.96 (s, 1H), 7.82 – 7.73 (m, 2H), 7.33 (dq, *J* = 7.8, 0.6 Hz, 2H), 2.44 (s, 3H).

^{13}C {^{1}H} NMR (101 MHz, $CDCl_3$) δ 192.2, 145.7, 134.4, 130.0, 129.9, 21.9.

4-(*tert*-Butyl)benzaldehyde (21)

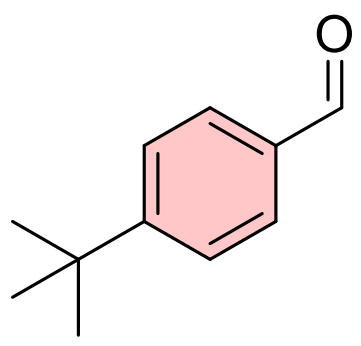

Following **GP-*b***, starting from 85 µL (0.5 mmol, 0.1M) of (4-(*tert*-butyl)phenyl)methanol (**1t**) and 20 mg of *g*-C_3N_4/$CsPbCl_3$ (90%/10%) catalyst in 5 mL of acetonitrile. The crude mixture was purified using silica gel chromatography (95:5 cyclohexane/ethyl acetate) to afford 47 mg of **21** (0.29 mmol, 58% yield) as a colorless dense oil. Spectroscopic data for **21** are in accordance with literature.[13]

^{1}H NMR (400 MHz, $CDCl_3$) δ 9.98 (s, 1H), 7.86 – 7.76 (m, 2H), 7.59 – 7.45 (m, 2H), 1.35 (s, 9H).

^{13}C {^{1}H} NMR (101 MHz, $CDCl_3$) δ 192.2, 158.6, 134.2, 129.8, 126.1, 35.5, 31.2.

4-Bromobenzaldehyde (22)

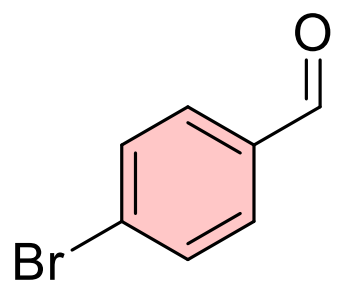

Following **GP-*b***, starting from 94 mg (0.5 mmol, 0.1M) of (4-bromophenyl)methanol (**1u**) and 20 mg of *g*-C_3N_4/$CsPbCl_3$ (90%/10%) catalyst in 5 mL of acetonitrile. The crude mixture was purified using silica gel chromatography (95:5 cyclohexane/ethyl acetate) to afford 60 mg of **22** (0.30 mmol, 60% yield) as a pale yellow oil. Spectroscopic data for **22** are in accordance with literature.[13]

^{1}H NMR (400 MHz, $CDCl_3$) δ 9.97 (s, 1H), 7.77 – 7.72 (m, 2H), 7.70 – 7.65 (m, 2H).

^{13}C {^{1}H} NMR (101 MHz, $CDCl_3$) δ 190.8, 134.9, 132.3, 130.8, 129.6.

4-Chlorobenzaldehyde (23)

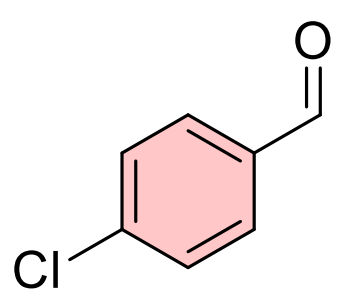

Following **GP-*b***, starting from 72 mg (0.5 mmol, 0.1M) of (4-chlorophenyl)methanol (**1v**) and 20 mg of *g*-C_3N_4/$CsPbCl_3$ (90%/10%) catalyst in 5 mL of acetonitrile. The crude mixture was purified using silica gel chromatography (95:5 cyclohexane/ethyl acetate) to afford 20 mg of **23** (0.14 mmol, 28% yield) as a colorless dense oil. Spectroscopic data for **23** are in accordance with literature.[13]

^{1}H NMR (400 MHz, $CDCl_3$) δ 9.98 (s, 1H), 7.88 – 7.77 (m, 2H), 7.55 – 7.47 (m, 2H).

^{13}C {^{1}H} NMR (101 MHz, $CDCl_3$) δ 191.0, 141.1, 134.9, 131.0, 129.6.

3-Methoxybenzaldehyde (24)

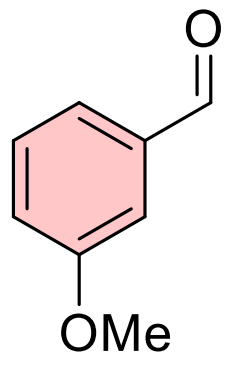

Following **GP-*b***, starting from 62 µL (0.5 mmol, 0.1M) of (3-methoxyphenyl)methanol (**1w**) and 20 mg of *g*-C_3N_4/$CsPbCl_3$ (90%/10%) catalyst in 5 mL of acetonitrile. The crude mixture was purified using silica gel chromatography (95:5 cyclohexane/ethyl acetate) to afford 39 mg of **24** (0.29 mmol, 58% yield) as a yellowish dense oil. Spectroscopic data for **24** are in accordance with literature.[14]

^{1}H NMR (400 MHz, $CDCl_3$) δ 9.97 (s, 1H), 7.50 – 7.41 (m, 2H), 7.40 – 7.38 (m, 1H), 7.17 (dt, *J* = 6.4, 2.7 Hz, 1H), 3.86 (s, 3H).

$^{13}C\{^1H\}$ NMR (101 MHz, $CDCl_3$) δ 192.3, 160.3, 138.0, 130.2, 123.7, 121.7, 112.2, 55.6.

2-Methoxybenzaldehyde (25)

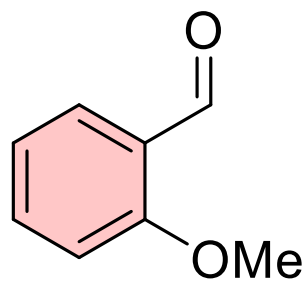

Following **GP-*b***, starting from 66 µL (0.5 mmol, 0.1M) of (2-methoxyphenyl)methanol (**1x**) and 20 mg of *g*-C_3N_4/$CsPbCl_3$ (90%/10%) catalyst in 5 mL of acetonitrile. The crude mixture was purified using silica gel chromatography (95:5 cyclohexane/ethyl acetate) to afford 35 mg of **25** (0.26 mmol, 52% yield) as a yellowish dense oil. Spectroscopic data for **25** are in accordance with literature.[13]

^{1}H NMR (400 MHz, $CDCl_3$) δ 10.47 (d, *J* = 0.8 Hz, 1H), 7.83 (dd, *J* = 7.7, 1.9 Hz, 1H), 7.55 (ddd, *J* = 8.4, 7.3, 1.9 Hz, 1H), 7.08 – 6.92 (m, 2H), 3.93 (s, 3H).

$^{13}C\{^1H\}$ NMR (101 MHz, $CDCl_3$) δ 190.0, 162.0, 136.1, 128.7, 125.0, 120.8, 111.8, 55.8.

3,4,5-Trimethoxybenzaldehyde (26)

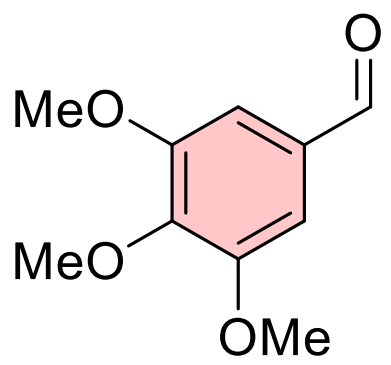

Following **GP-*b***, starting from 83 µL (0.5 mmol, 0.1M) of (3,4,5-trimethoxyphenyl)methanol (**1y**) and 20 mg of *g*-C_3N_4/$CsPbCl_3$ (90%/10%) catalyst in 5 mL of acetonitrile. The crude mixture was purified using silica gel chromatography (95:5 cyclohexane/ethyl acetate) to afford 25 mg of **26** (0.13 mmol, 26% yield) as a white solid (m.p.: 72-75°C; Lit.:[15] 70-73°C). Spectroscopic data for **26** are in accordance with literature.[6]

^{1}H NMR (400 MHz, $CDCl_3$) δ 9.86 (s, 1H), 7.13 (s, 2H), 3.94 (s, 3H), 3.92 (s, 6H).

$^{13}C\{^1H\}$ NMR (101 MHz, $CDCl_3$) δ 191.2, 153.8, 143.8, 131.9, 106.9, 61.1, 56.4.

4. References

[1] C. Callegari, C. Tedesco, A. Corbo, M. Prato, L. Malavasi, D. Ravelli, "Application of Lead-Free Metal Halide Perovskite Heterojunctions for the Carbohalogenation of C–C Multiple Bonds" *Org. Lett.* **2025**, *27*, 3667–3672.

[2] F. Puga, J. A. Navío, M. C. Hidalgo, "A critical view about use of scavengers for reactive species in heterogeneous photocatalysis" *Appl. Catal. A Gen.* **2024**, *685*, 119879.

[3] M. Medina-Llamas, E. Bianchi, M. C. Mozzati, C. Tedesco, C. Milanese, A. Speltini, A. Profumo, V. Armenise, A. Milella, A. Listorti, L. Malavasi, "Synthesis of Carbon Nitride Polymorphs by Sacrificial Template Method: Correlation between Physicochemical Properties and Photocatalytic Performance" *ChemSusChem* **2025**, *18*, DOI 10.1002/cssc.202400918.

[4] T. Constantin, M. Zanini, A. Regni, N. S. Sheikh, F. Juliá, D. Leonori, "Aminoalkyl radicals as halogen-atom transfer agents for activation of alkyl and aryl halides" *Science (1979).* **2020**, *367*, 1021–1026.

[5] J. Zhang, S. Liu, Y. Guo, Y. Liu, K. Zhang, X. Tan, L. Fang, "Trichloroacetonitrile-Mediated Oxidation of Sulfides and Benzylic C–H Bonds via Photoexcited Radical Reactions under Clean Conditions" *Org. Lett.* **2025**, *27*, 5606–5611.

[6] W. Dai, Y. Lv, L. Wang, S. Shang, B. Chen, G. Li, S. Gao, "Highly efficient oxidation of alcohols catalyzed by a porphyrin-inspired manganese complex" *Chemical Communications* **2015**, *51*, 11268–11271.

[7] Y. Yang, A. Qin, K. Zhao, D. Wang, X. Shi, "Design and Synthesis of Alanine Triazole Ligands and Application in Promotion of Hydration, Allene Synthesis and Borrowing Hydrogen Reactions" *Adv. Synth. Catal.* **2016**, *358*, 1433–1439.

[8] K. Moriyama, M. Takemura, H. Togo, "Direct and Selective Benzylic Oxidation of Alkylarenes via C–H Abstraction Using Alkali Metal Bromides" *Org. Lett.* **2012**, *14*, 2414–2417.

[9] S. Zhu, P. Hu, M. Guo, L. Zhao, L. Yang, W.-J. Gu, W. Han, "Eco-friendly iron-catalyzed oxidation of unstrained tertiary aromatic alcohols to ketones" *Chinese Chemical Letters* **2024**, *35*, 108835.

[10] W. Qian, L. Zhang, H. Sun, H. Jiang, H. Liu, "Microwave-Assisted One-Step Synthesis of Acetophenones *via* Palladium-Catalyzed Regioselective Arylation of Vinyloxytrimethylsilane" *Adv. Synth. Catal.* **2012**, *354*, 3231–3236.

[11] Y. Li, T. Ye, F. Li, S. Tan, "Chalcogen-Bonding-Enabled, Light-Driven Decarboxylative Oxygenation of Amino Acid Derivatives and Short Peptides Using O_2" *Angew. Chem. Int. Ed.* **2025**, *64*, DOI 10.1002/anie.202502233.

[12] M. Hayashi, M. Shibuya, Y. Iwabuchi, "Oxidation of Alcohols to Carbonyl Compounds with Diisopropyl Azodicarboxylate Catalyzed by Nitroxyl Radicals" *J. Org. Chem.* **2012**, *77*, 3005–3009.

[13] Y. Liu, W. Xiao, Y. Zhang, L. Ou, Z. Yi, X. Yang, H. Fu, "CF_3SO_2Na-promoted photocatalytic aerobic oxidation of benzyl alcohols to aromatic aldehydes and aromatic esters" *Org. Biomol. Chem.* **2025**, *23*, 3629–3633.

[14] A. B. Leduc, T. F. Jamison, "Continuous Flow Oxidation of Alcohols and Aldehydes Utilizing Bleach and Catalytic Tetrabutylammonium Bromide" *Org. Process Res. Dev.* **2012**, *16*, 1082–1089.

[15] M. Sheykhan, A. Karimi, M. Bahmani, M. Rezanoor, M. Abbasnia, "MOF and Visible Light in Synergy: Decarboxylations Catalyzed by Metal–Organic Frameworks" *Org. Lett.* **2025**, *27*, 11884–11889.

5. Copy of ^{1}H and ^{13}C NMR spectra

1-(4-Methoxyphenyl)ethan-1-one (2)

^{1}H-NMR (400MHz)

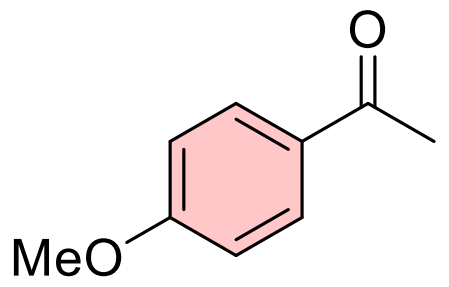

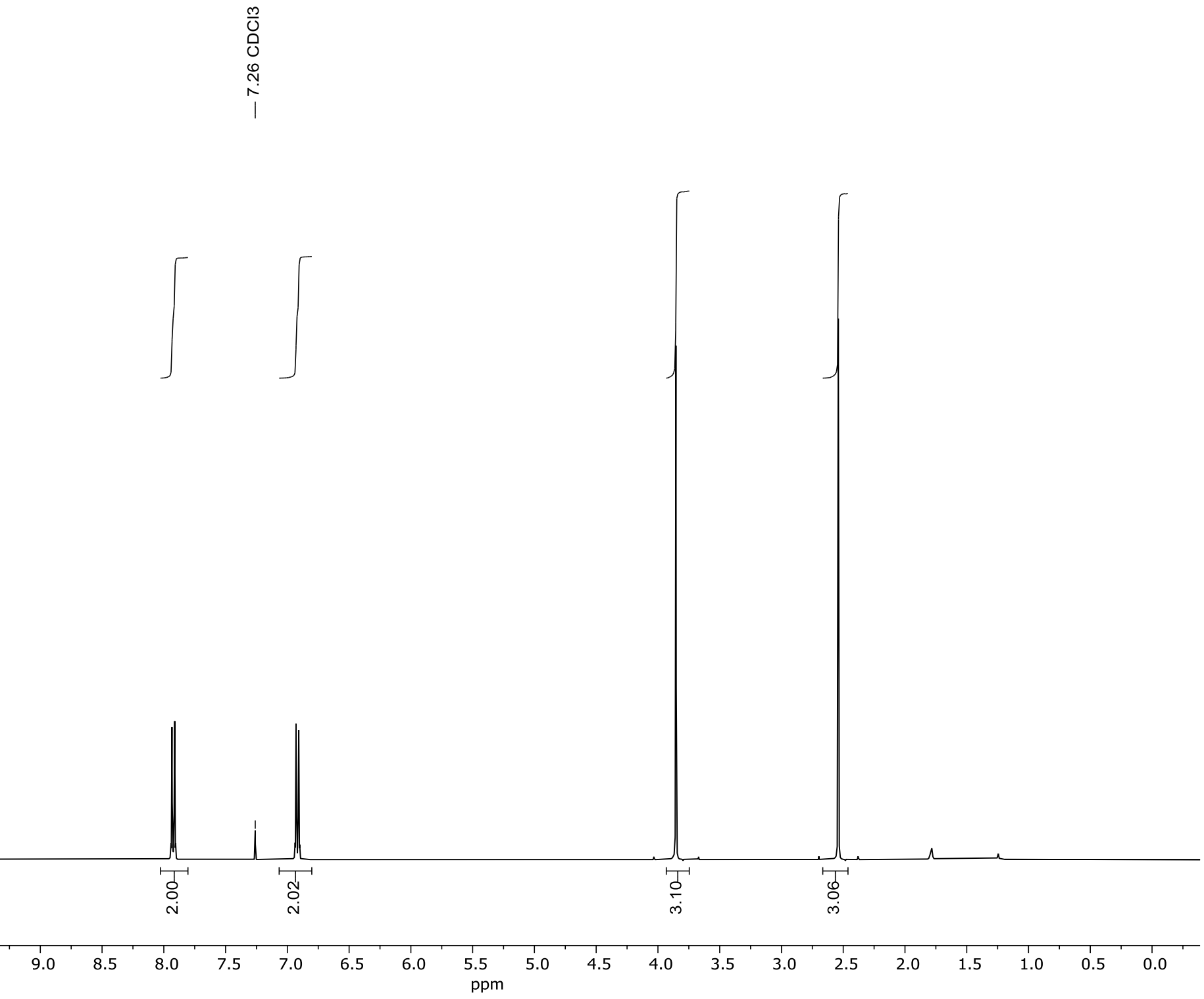

^{13}C-NMR (101MHz)

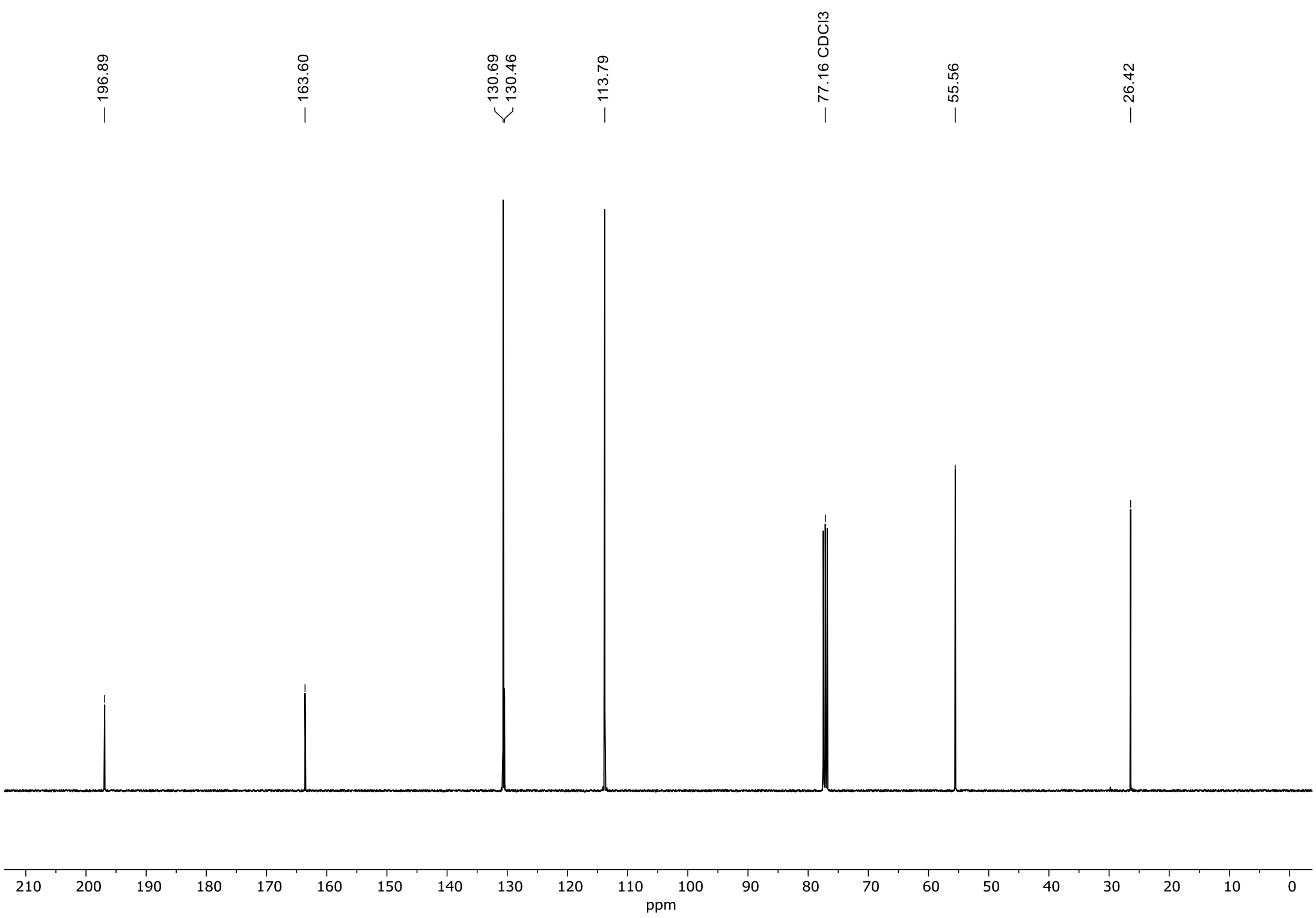

1-(*p*-Tolyl)ethan-1-one (3)

^{1}H-NMR (400MHz)*

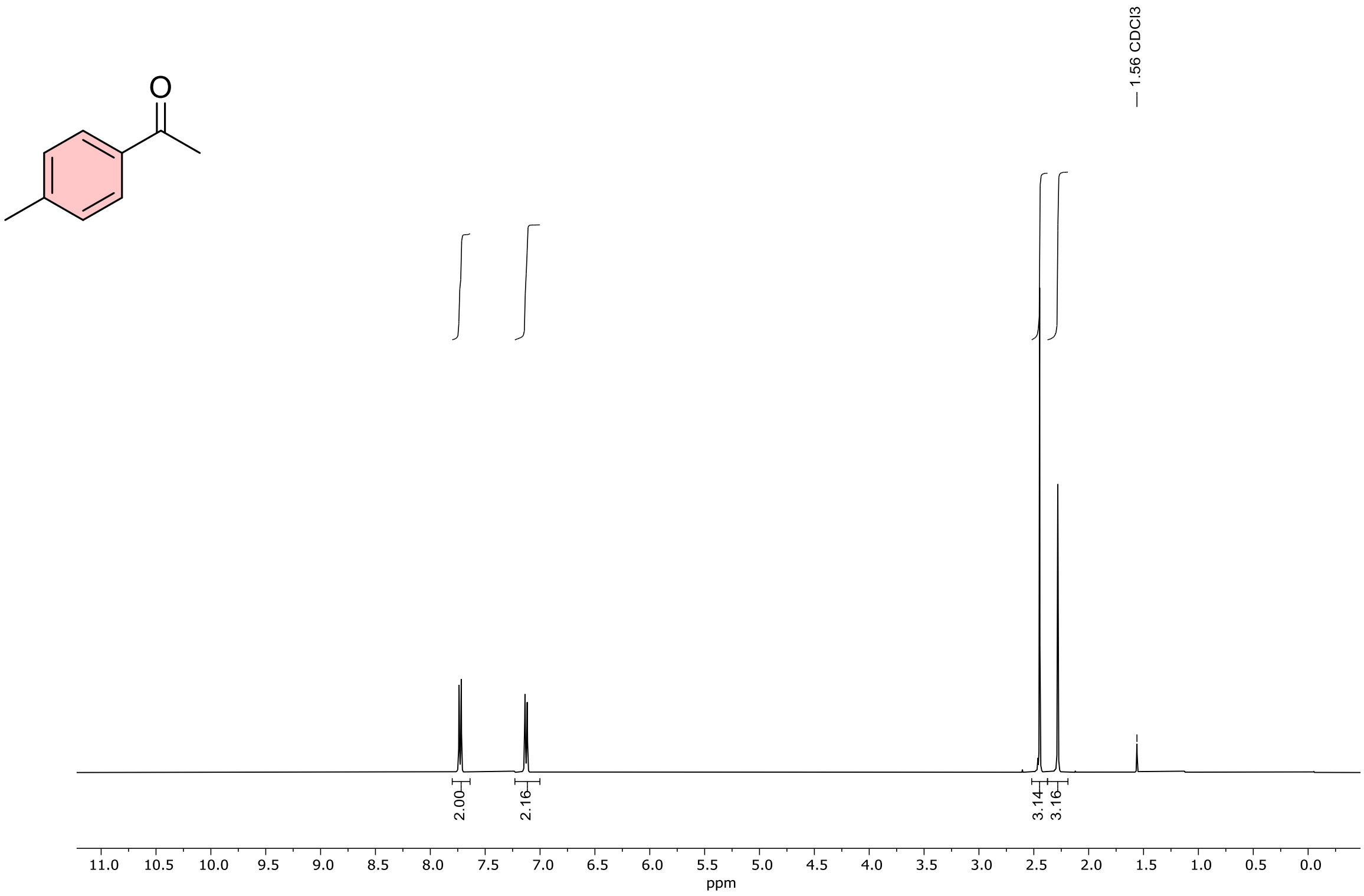

^{13}C-NMR (101MHz)

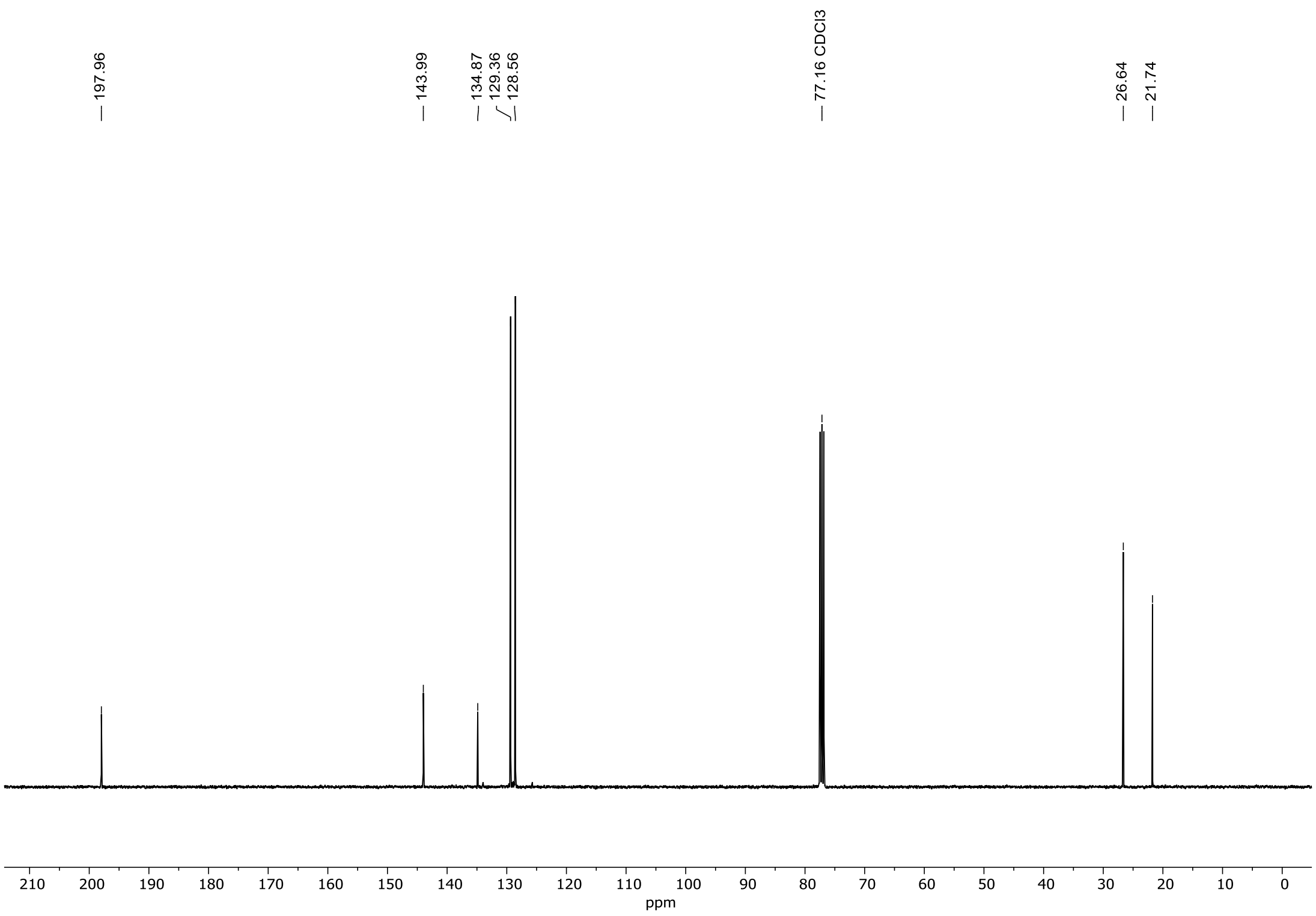

* The ppm scale was referred to the residual residual water peak in $CDCl_3$ (corresponding to 1.56 ppm chemical shift, according to the Mestrenova software database), due to the superimposition of the 7.26 ppm peak with other signals.

1-(4-Bromophenyl)ethan-1-one (4)

^{1}H-NMR (400MHz)

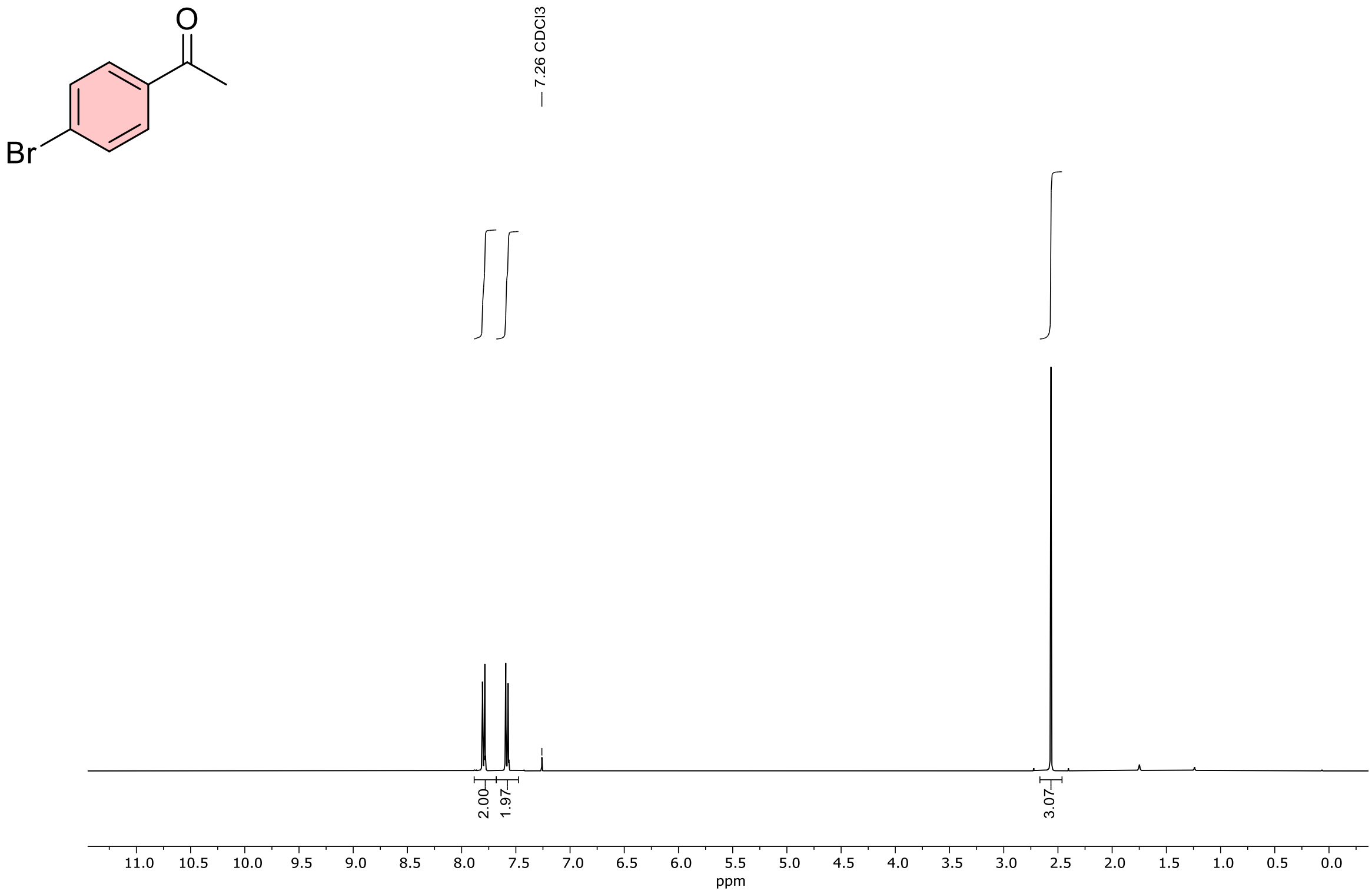

^{13}C-NMR (101MHz)

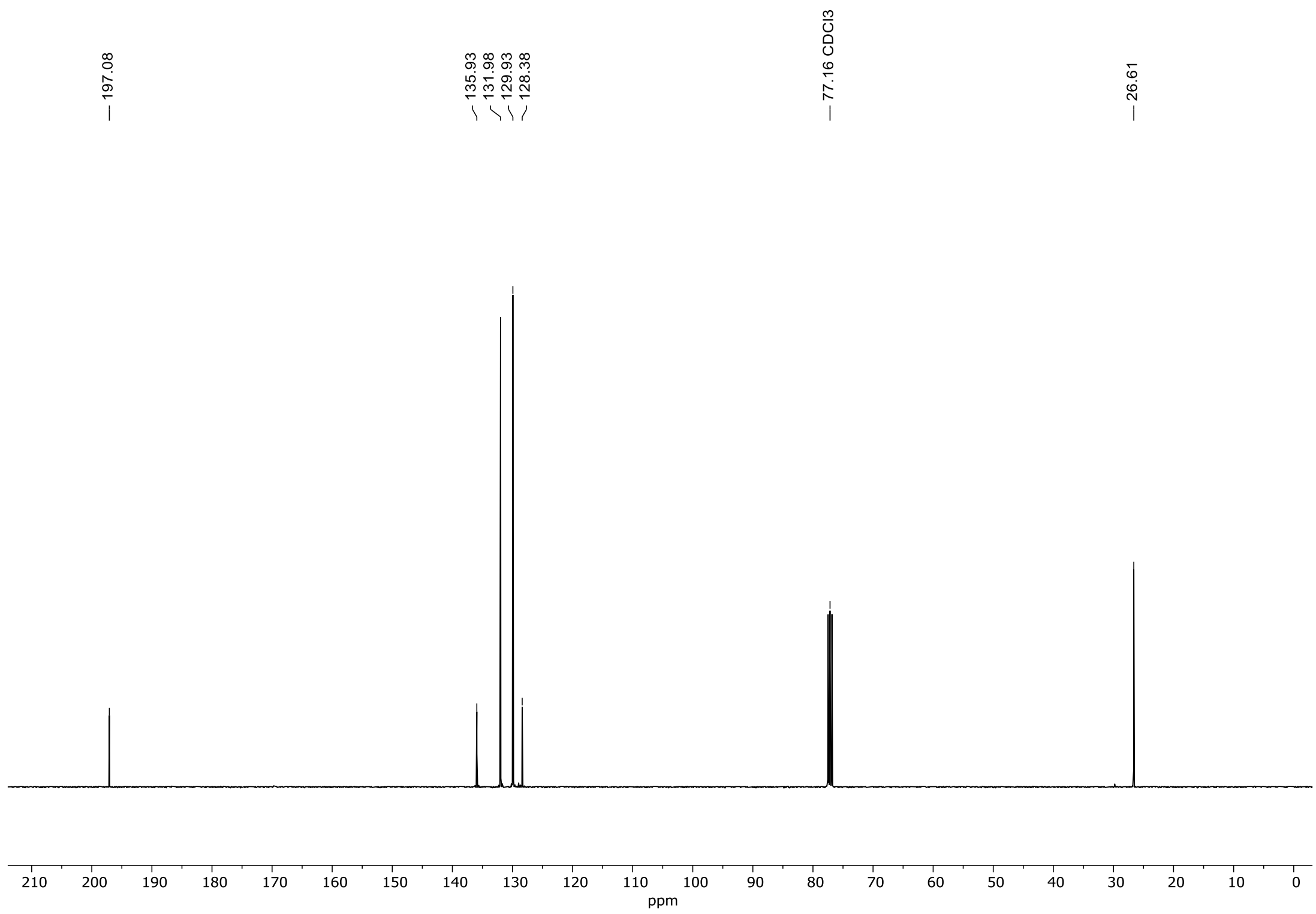

1-(4-Chlorophenyl)ethan-1-one (5)

^{1}H-NMR (400MHz)

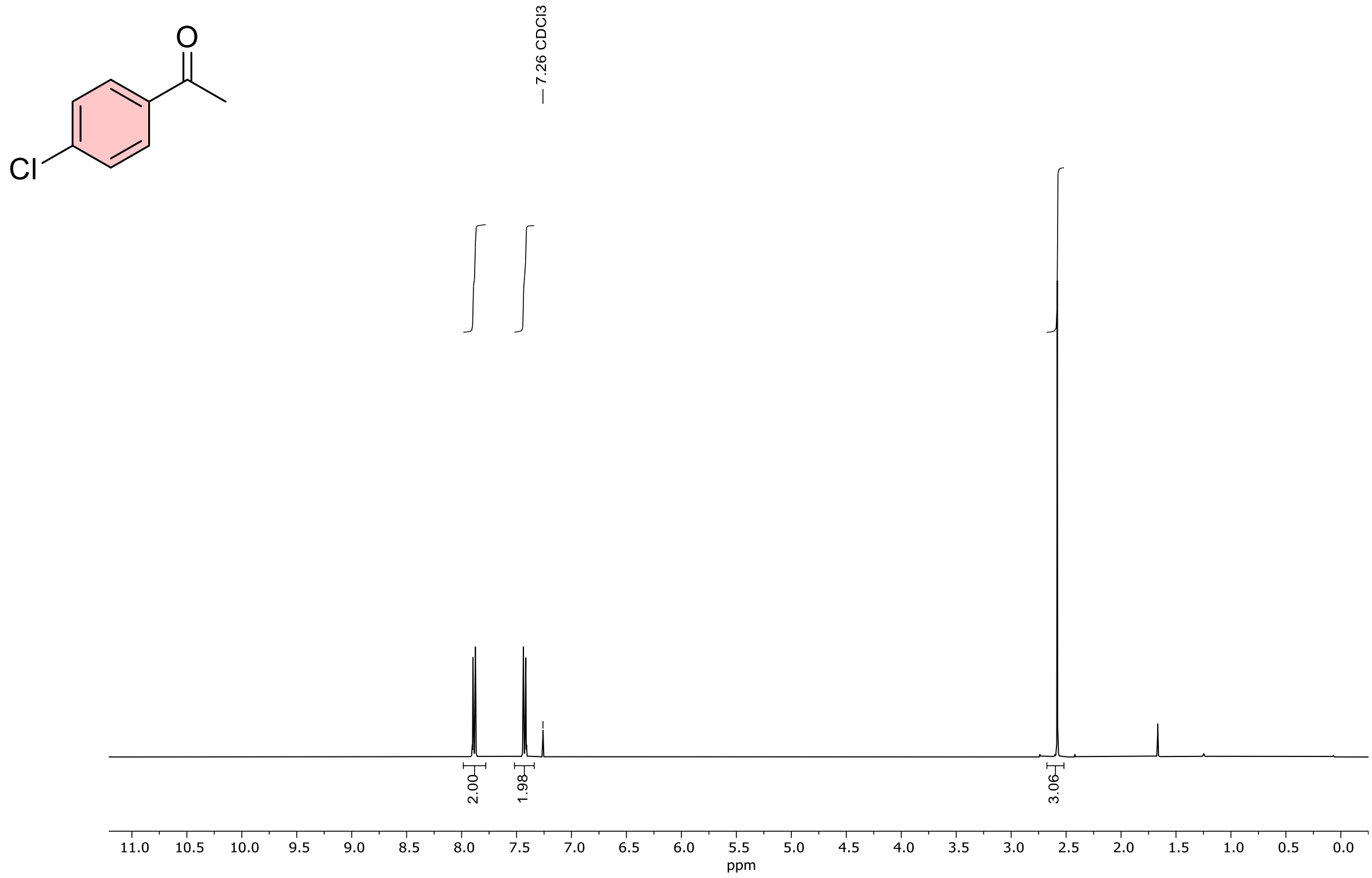

^{13}C-NMR (101MHz)

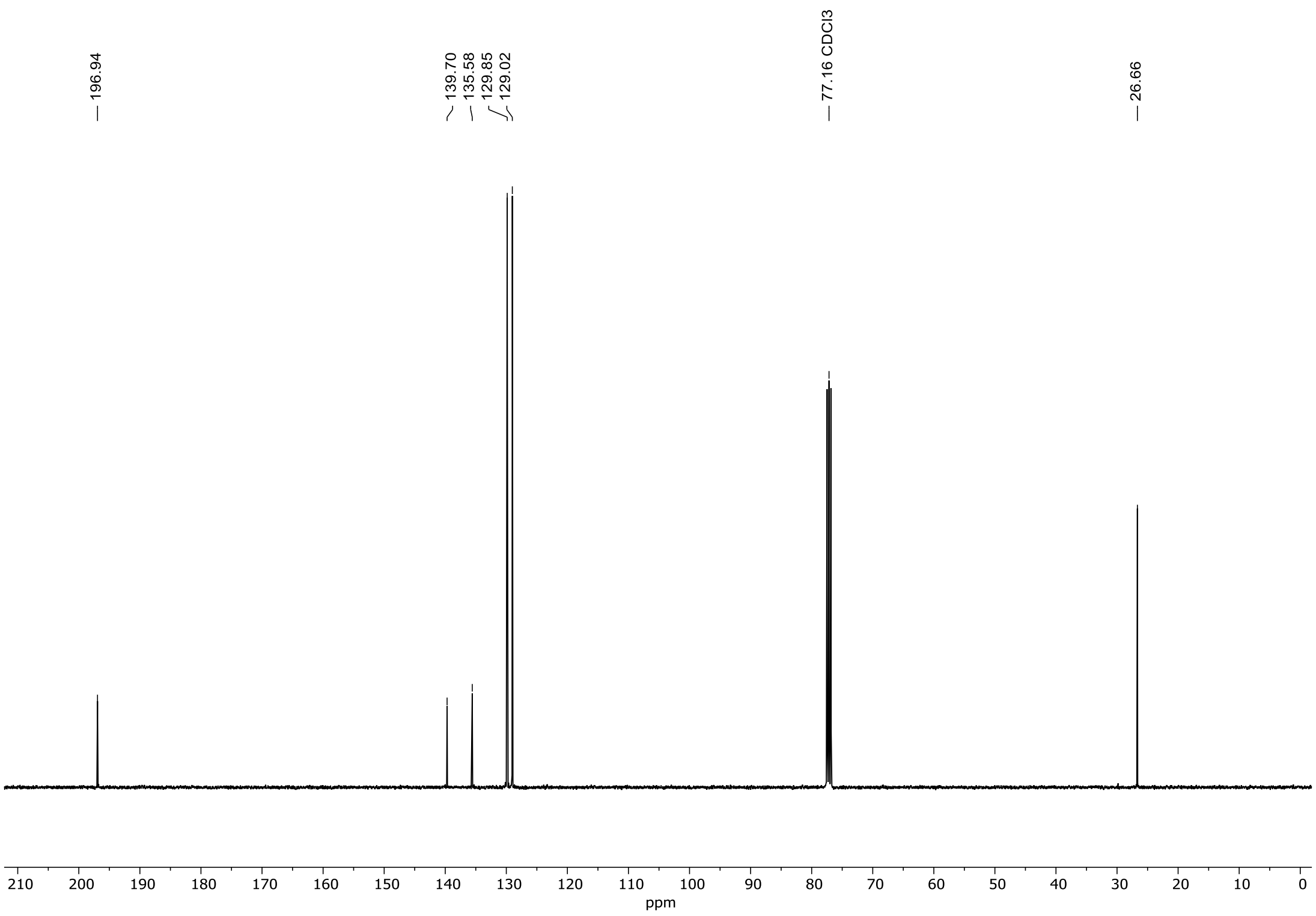

1-(3-Methoxyphenyl)ethan-1-one (7)

^{1}H-NMR (400MHz)

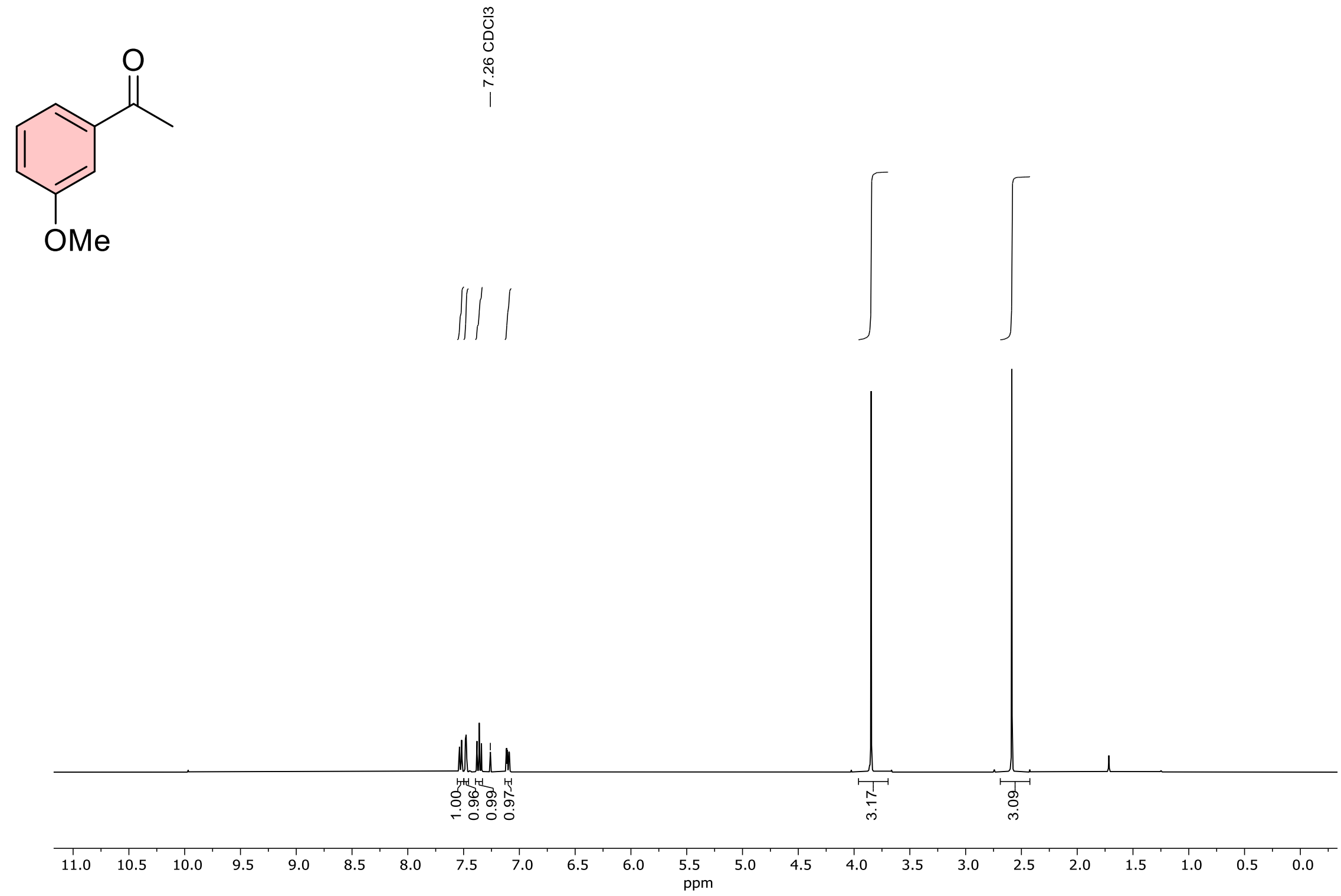

^{13}C-NMR (101MHz)

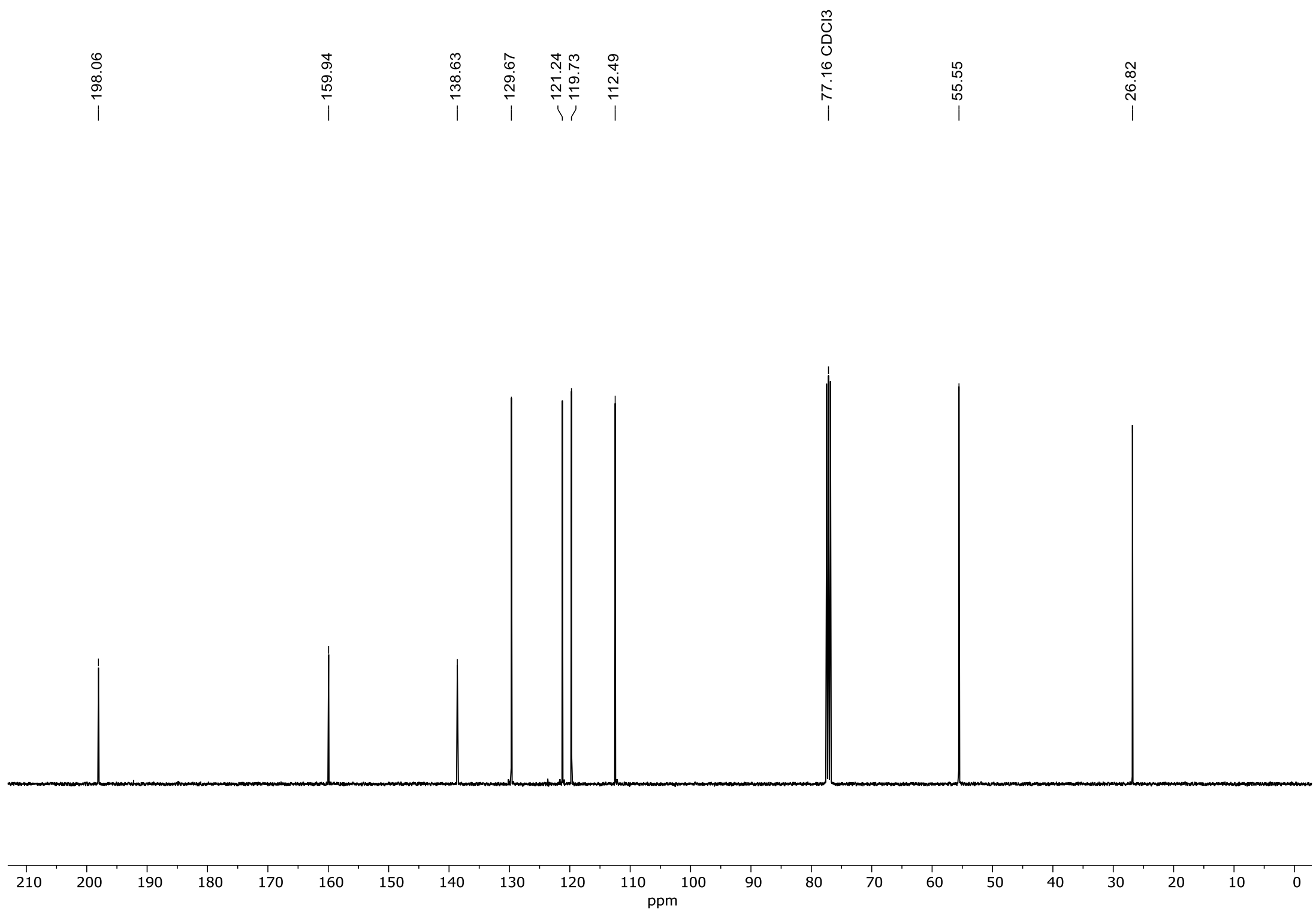

1-(*m*-Tolyl)ethan-1-one (8)

^{1}H-NMR (400MHz)

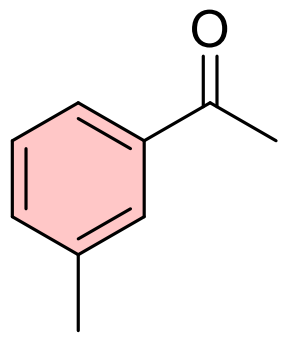

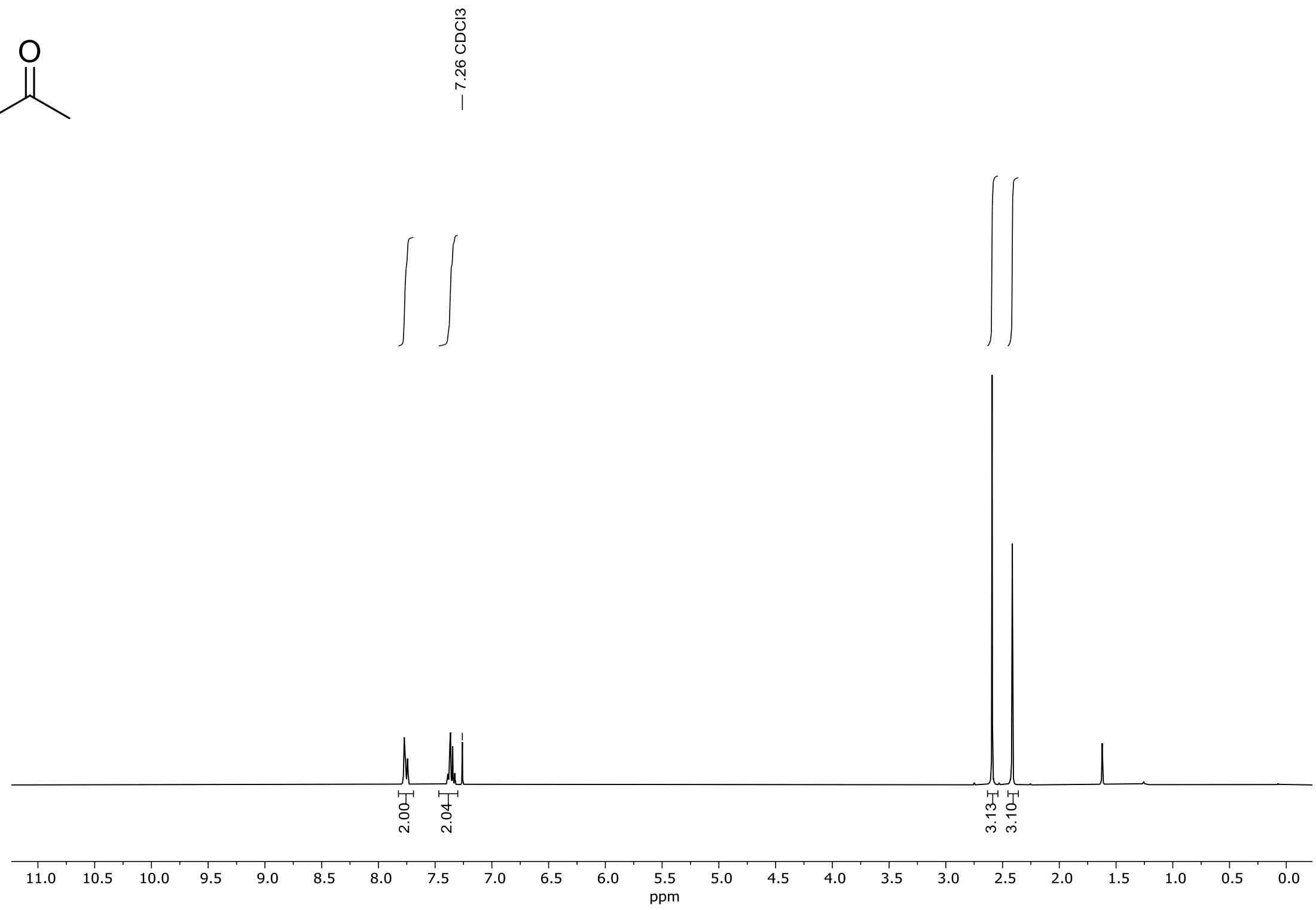

^{13}C-NMR (101MHz)

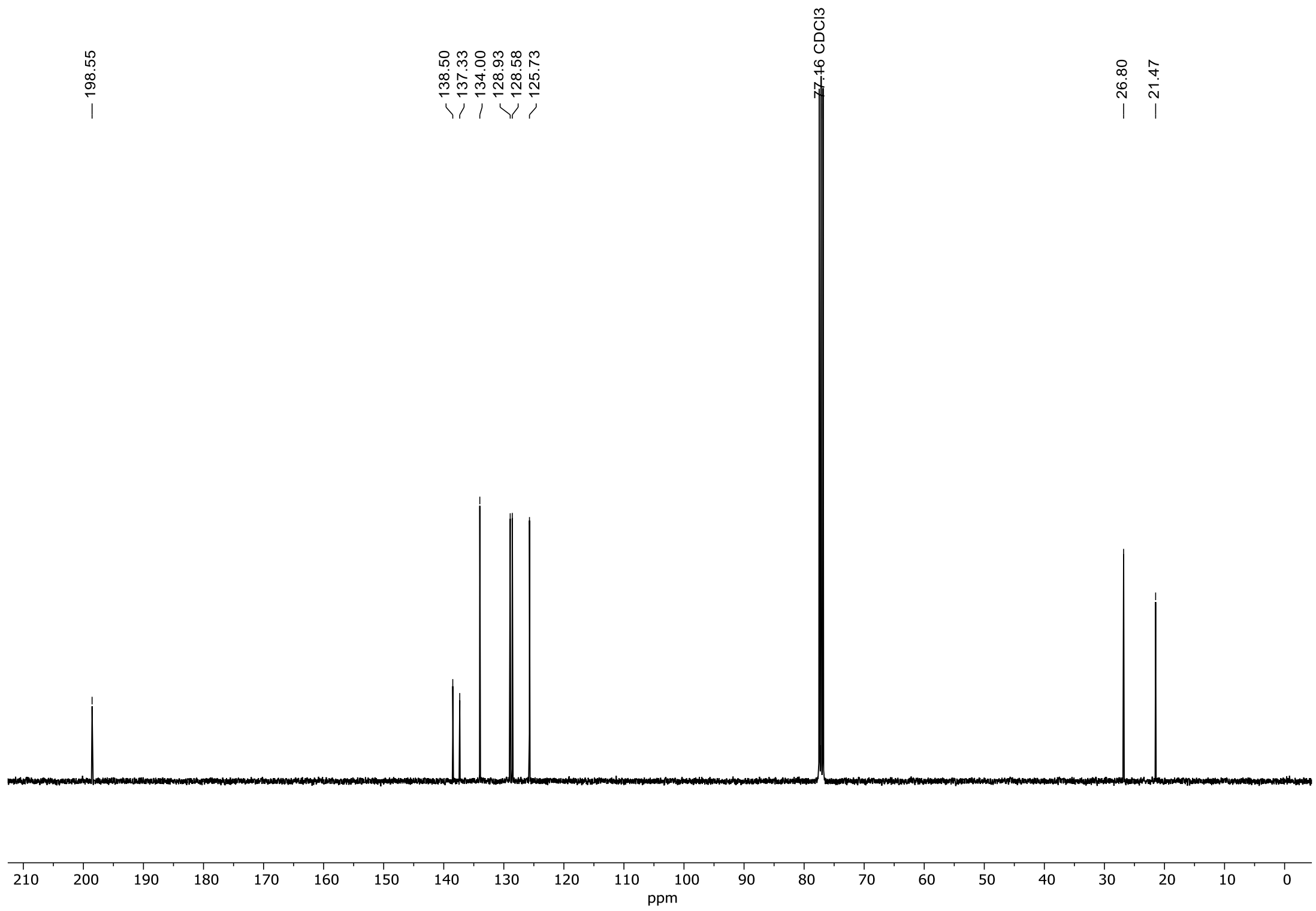

1-(3-Bromophenyl)ethan-1-one (9)

^{1}H-NMR (400MHz)

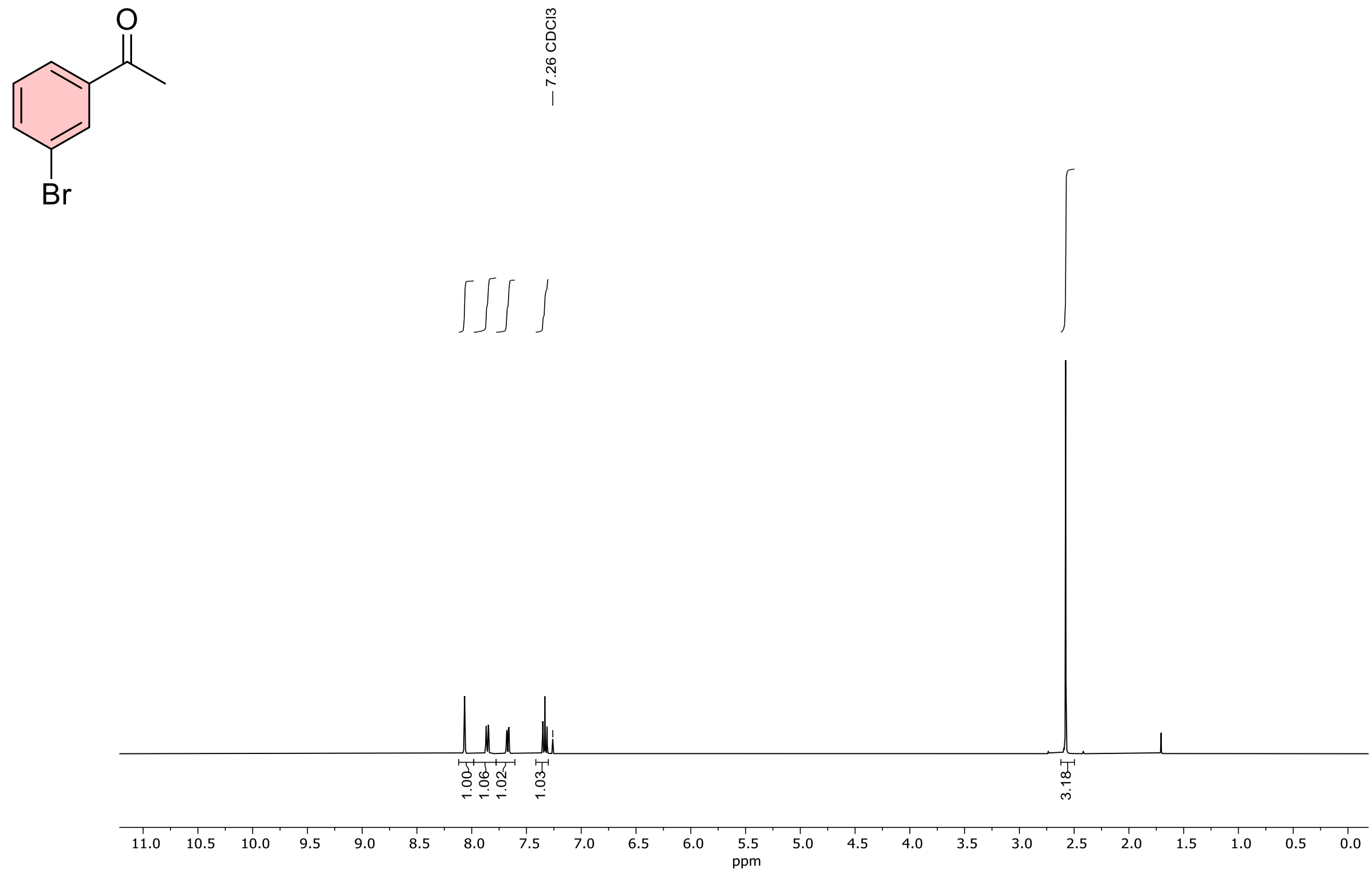

^{13}C-NMR (101MHz)

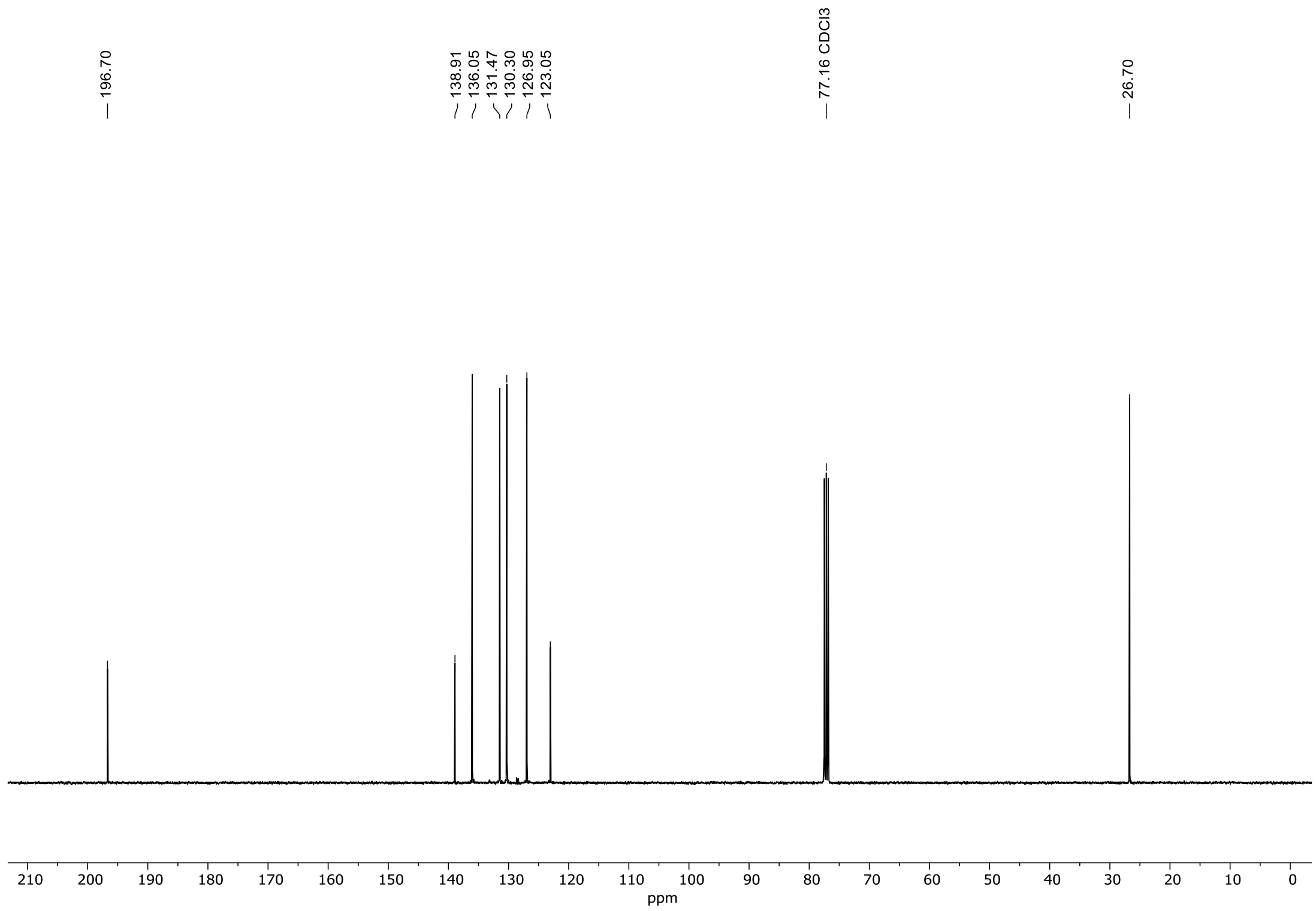

1-(3-Chlorophenyl)ethan-1-one (10)

^{1}H-NMR (400MHz)

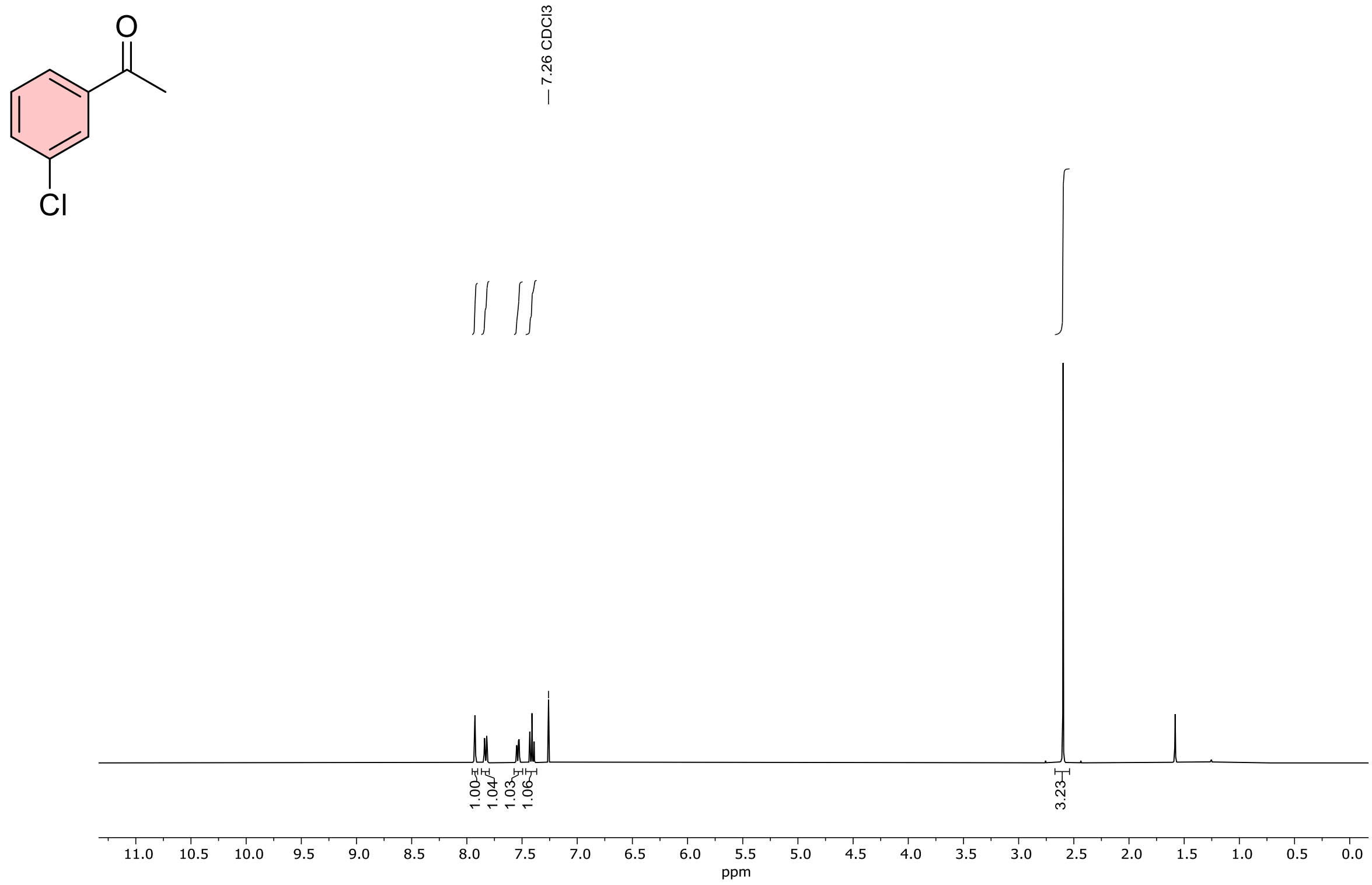

^{13}C-NMR (101MHz)

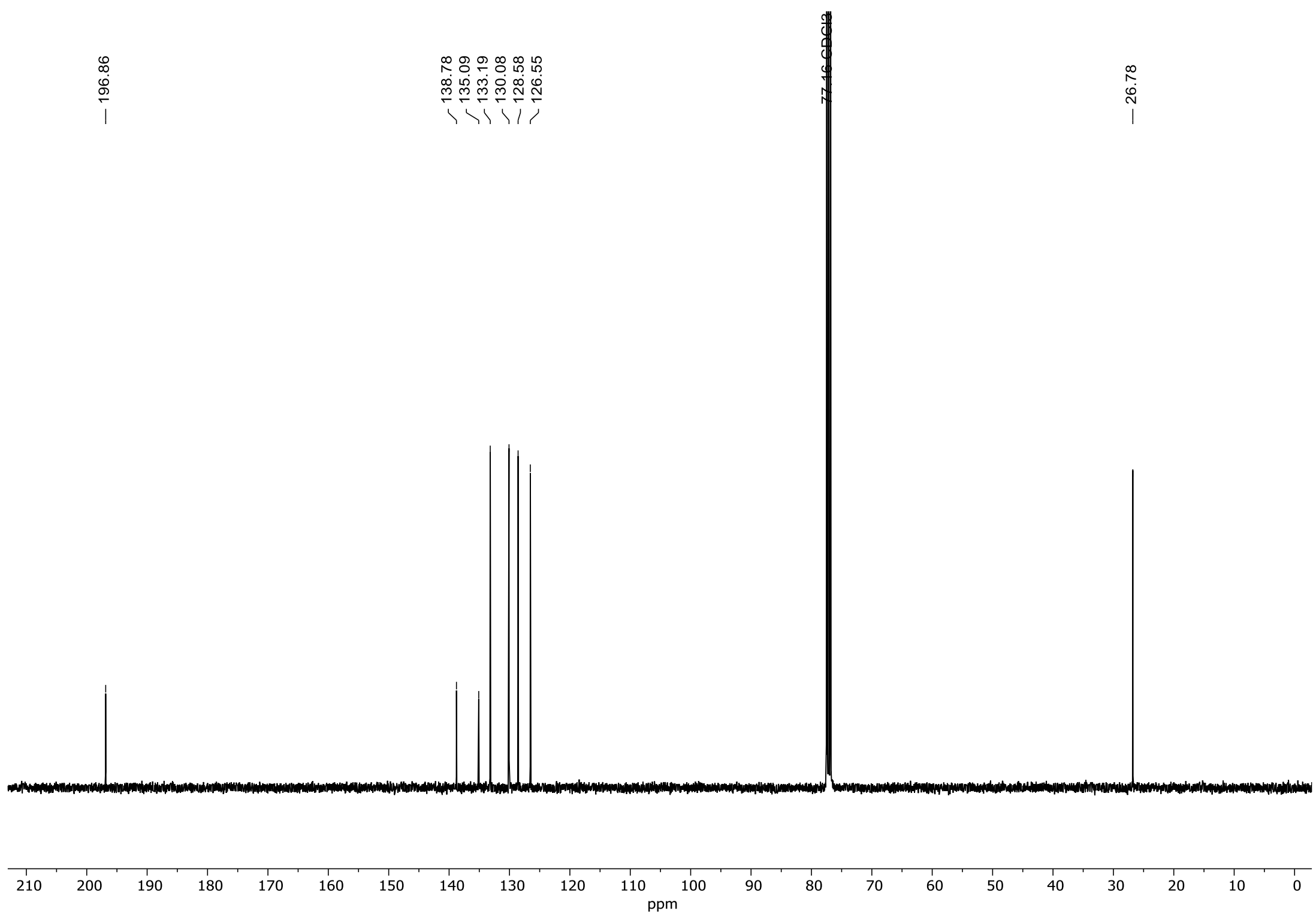

1-(2-Methoxyphenyl)ethan-1-one (11)

^{1}H-NMR (400MHz)

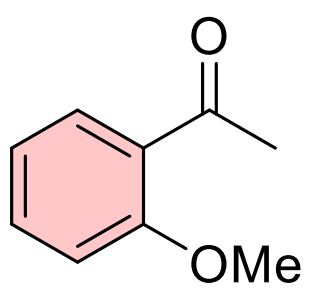

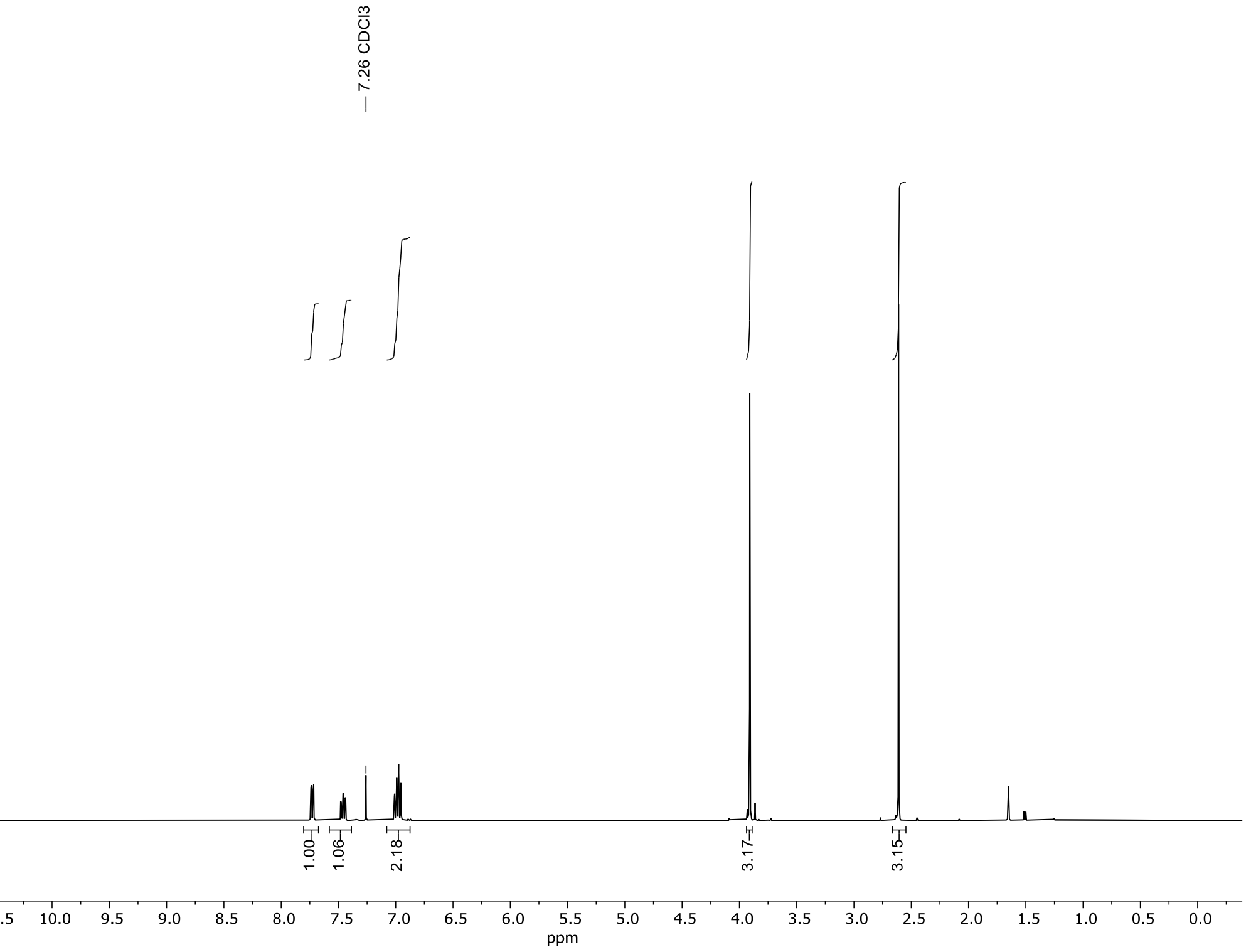

^{13}C-NMR (101MHz)

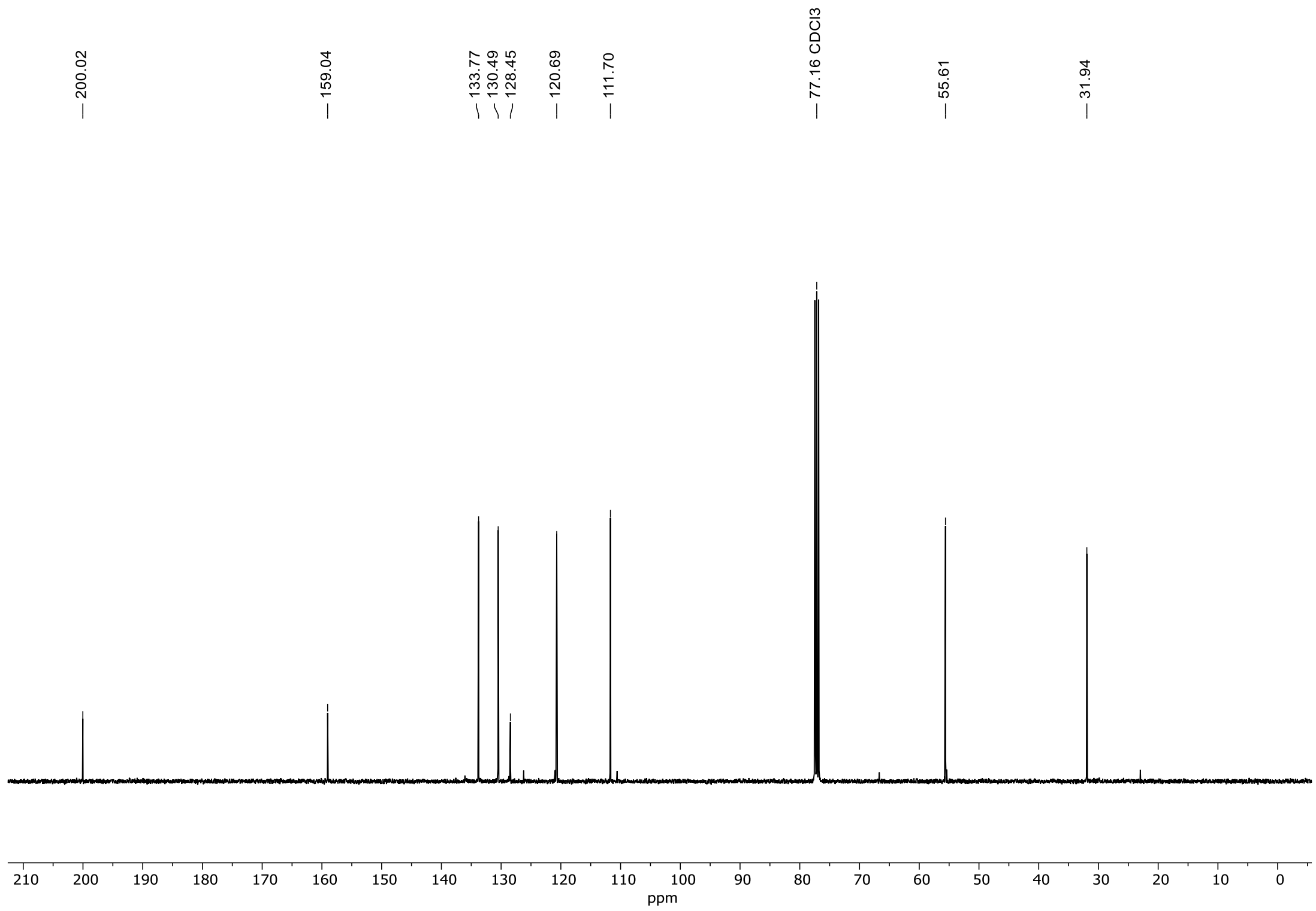

1-(2-Bromophenyl)ethan-1-one (13)

^{1}H-NMR (400MHz)

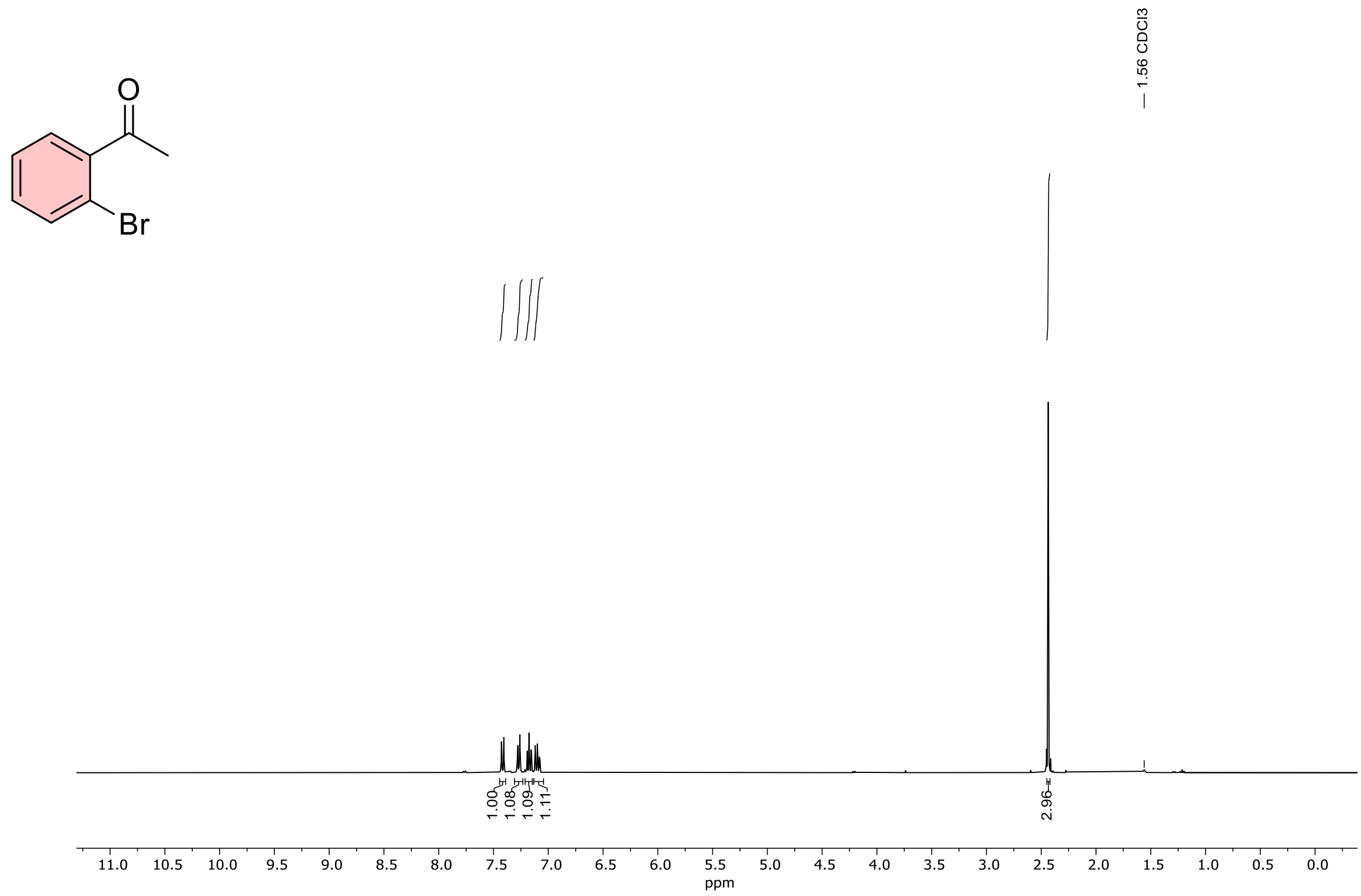

^{13}C-NMR (101MHz)

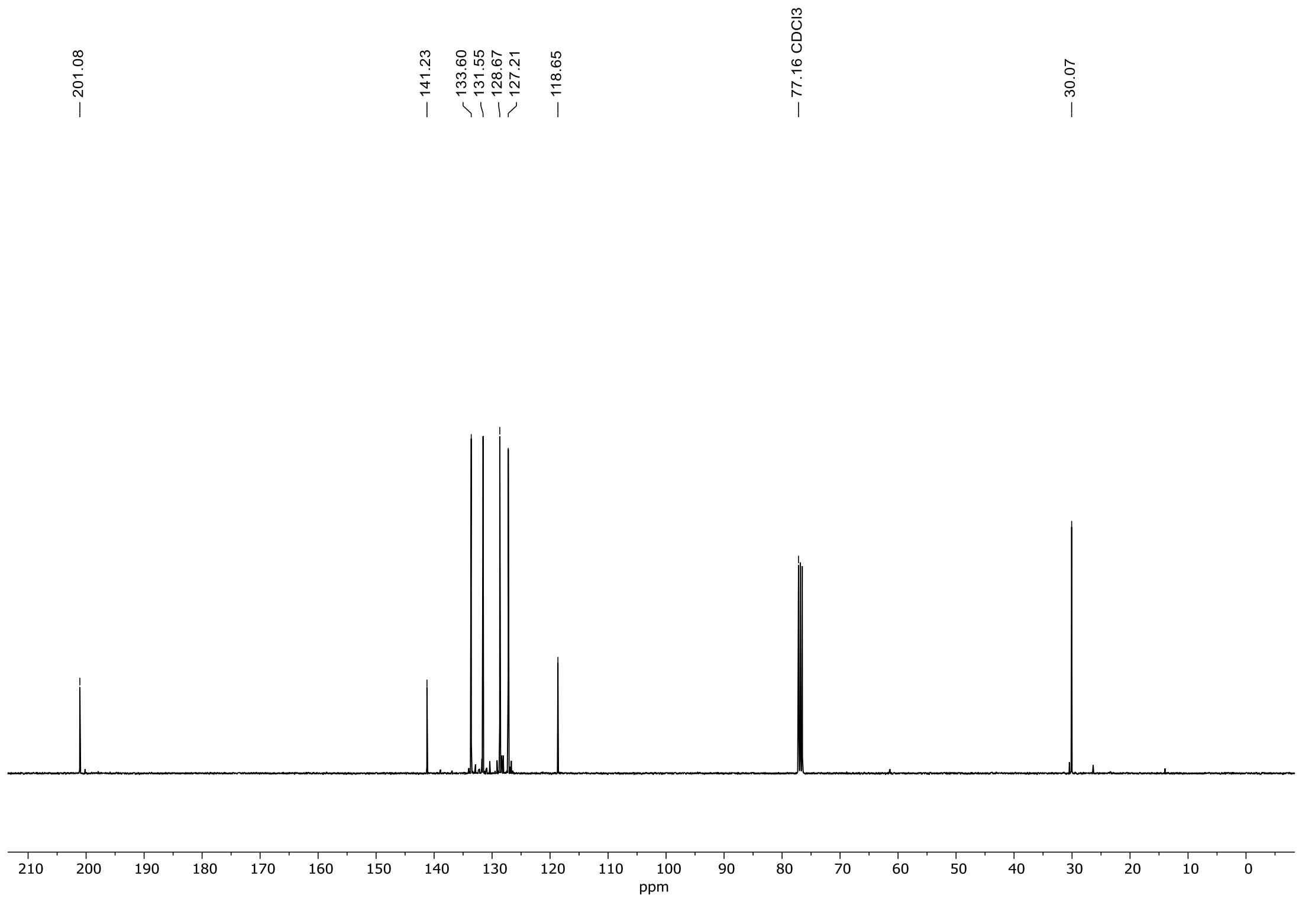

* The ppm scale was referred to the residual residual water peak in $CDCl_3$ (corresponding to 1.56 ppm chemical shift, according to the Mestrenova software database), due to the superimposition of the 7.26 ppm peak with other signals.

1-(2-Chlorophenyl)ethan-1-one (14)

^{1}H-NMR (400MHz)

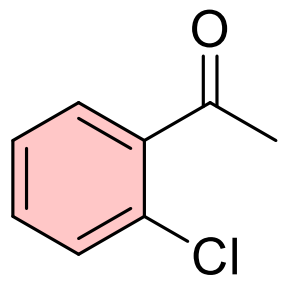

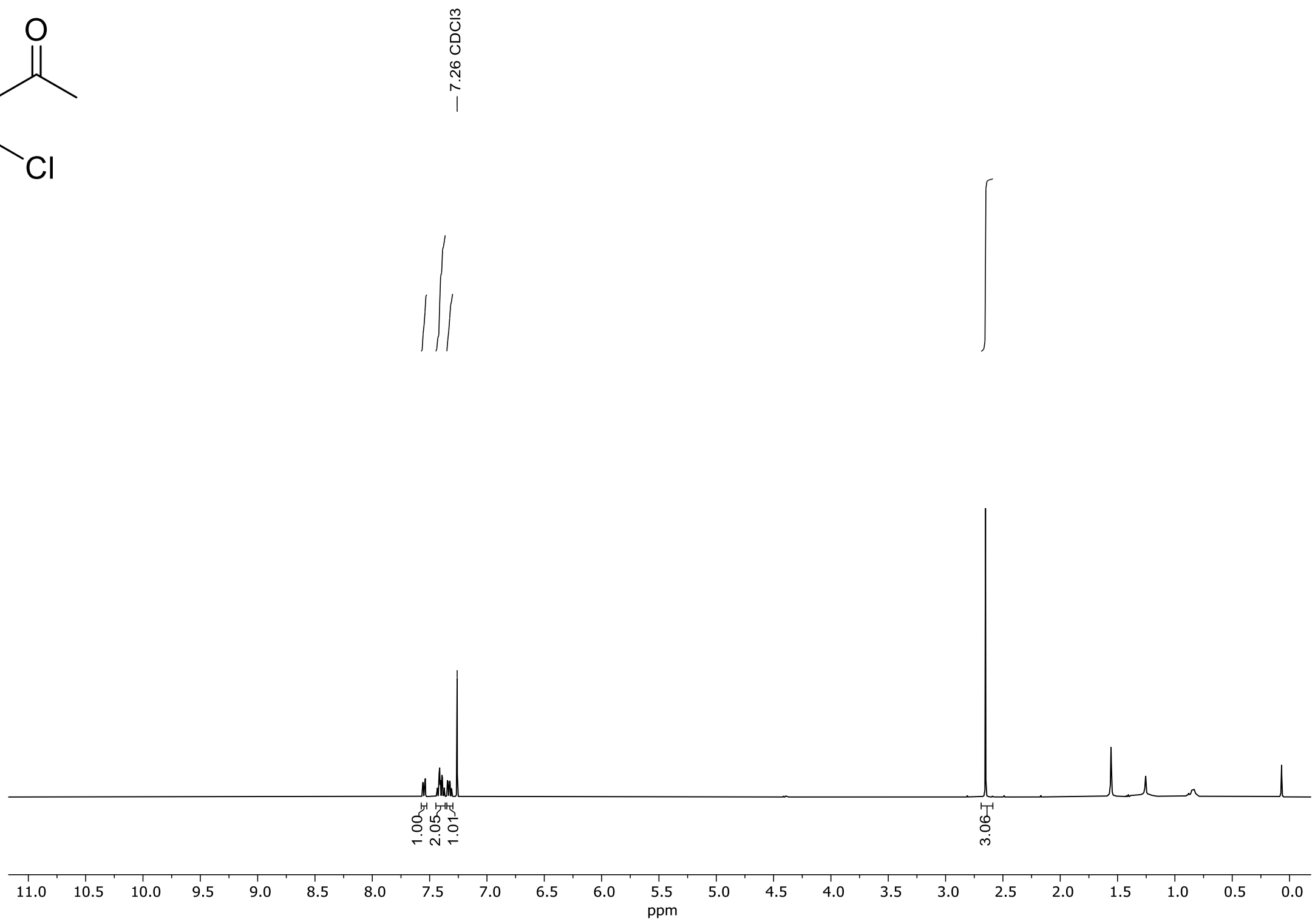

^{13}C-NMR (101MHz)

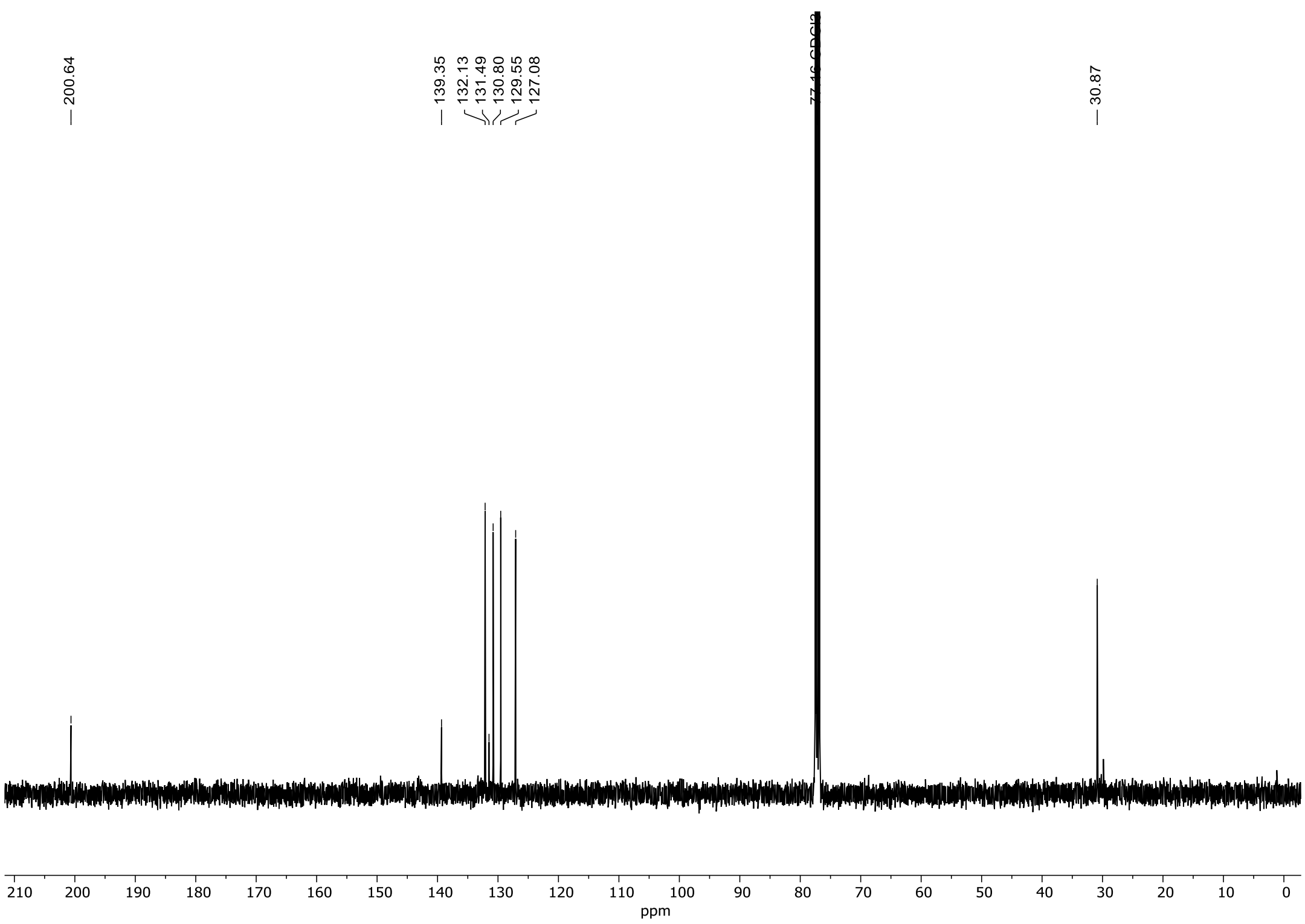

Acetophenone (15)

^{1}H-NMR (400MHz)

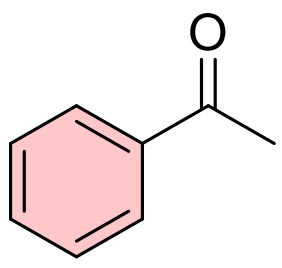

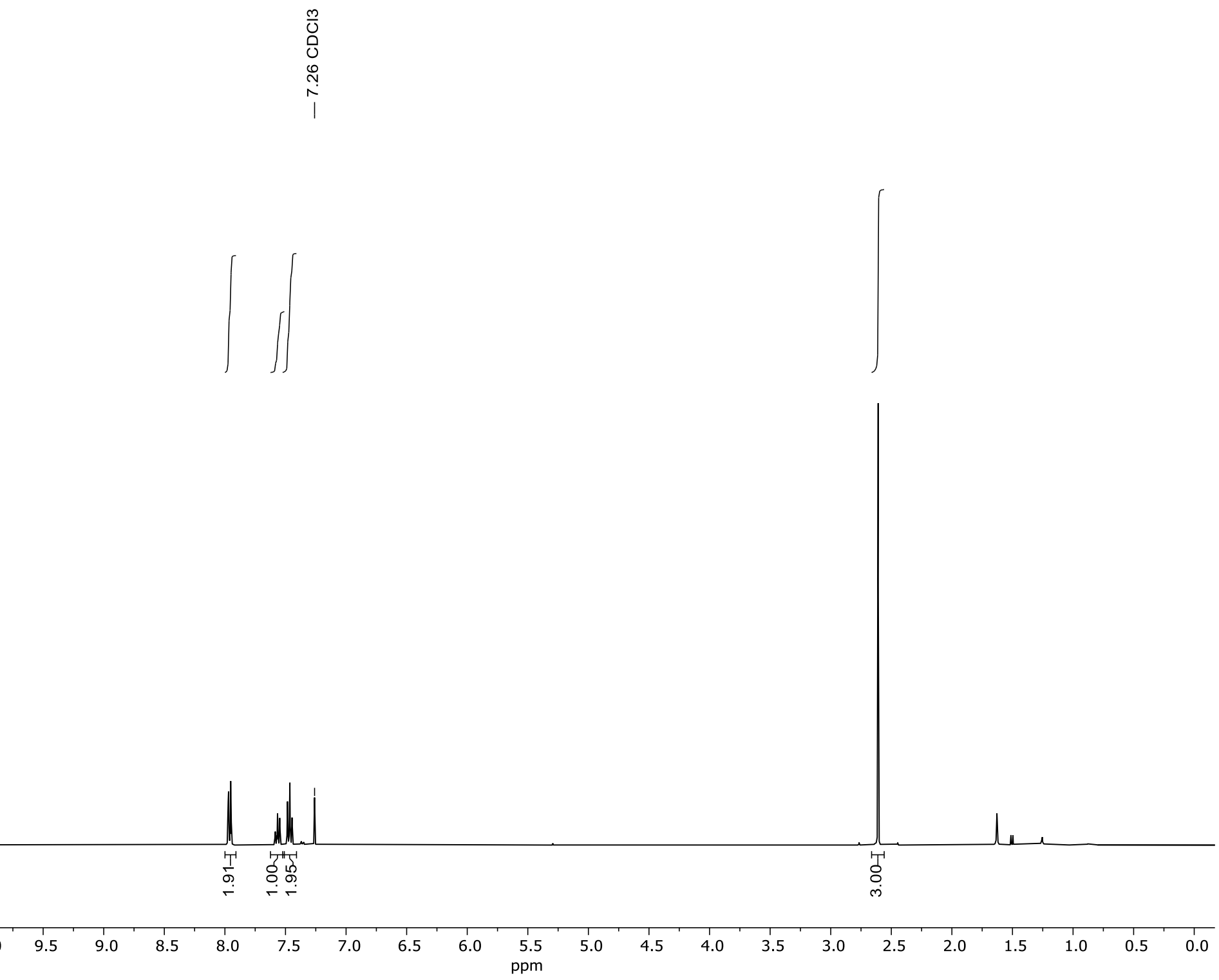

^{13}C-NMR (101MHz)

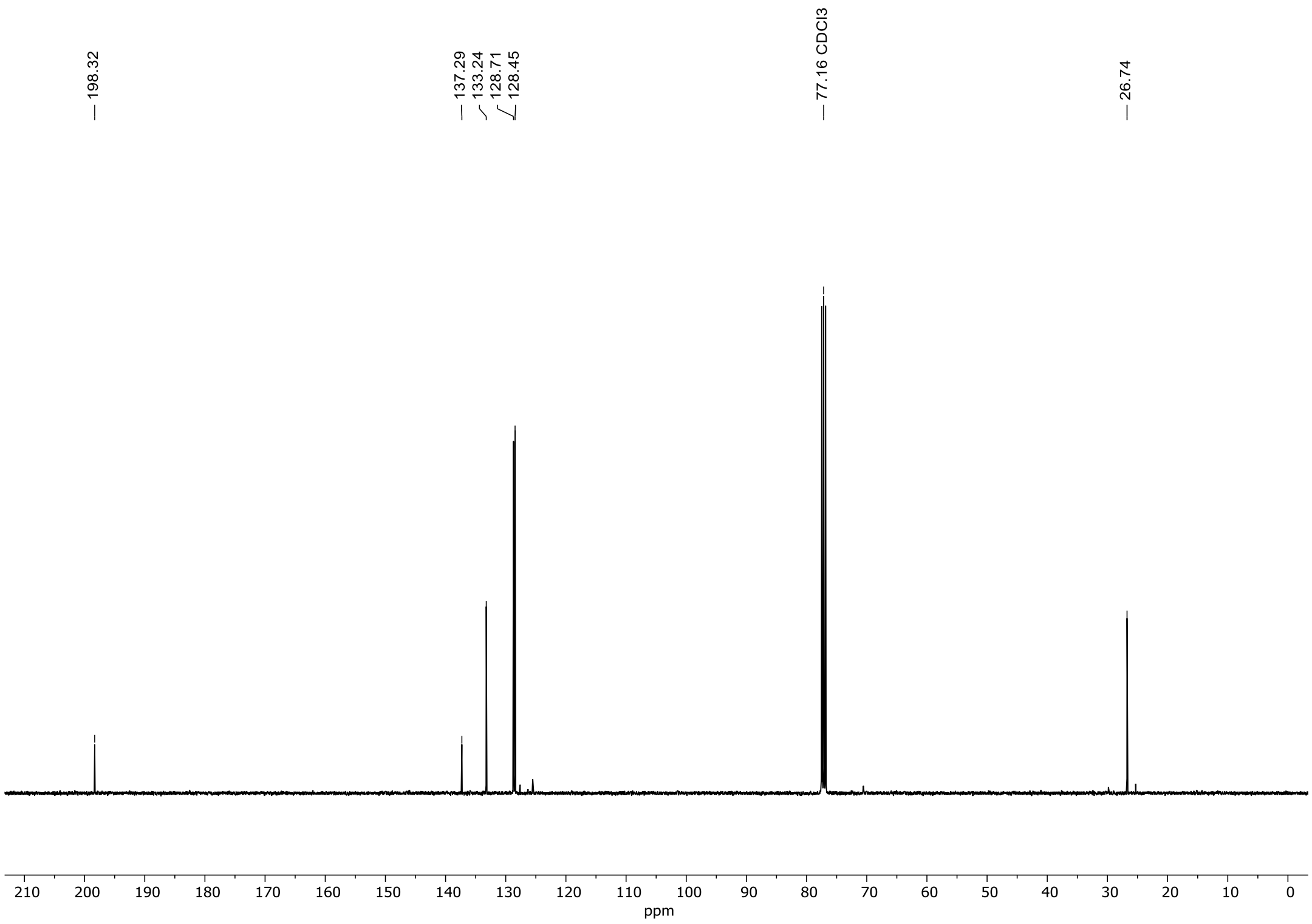

2,3-Dihydro-1H-inden-1-one (17)

^{1}H-NMR (400MHz)

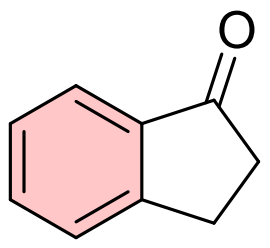

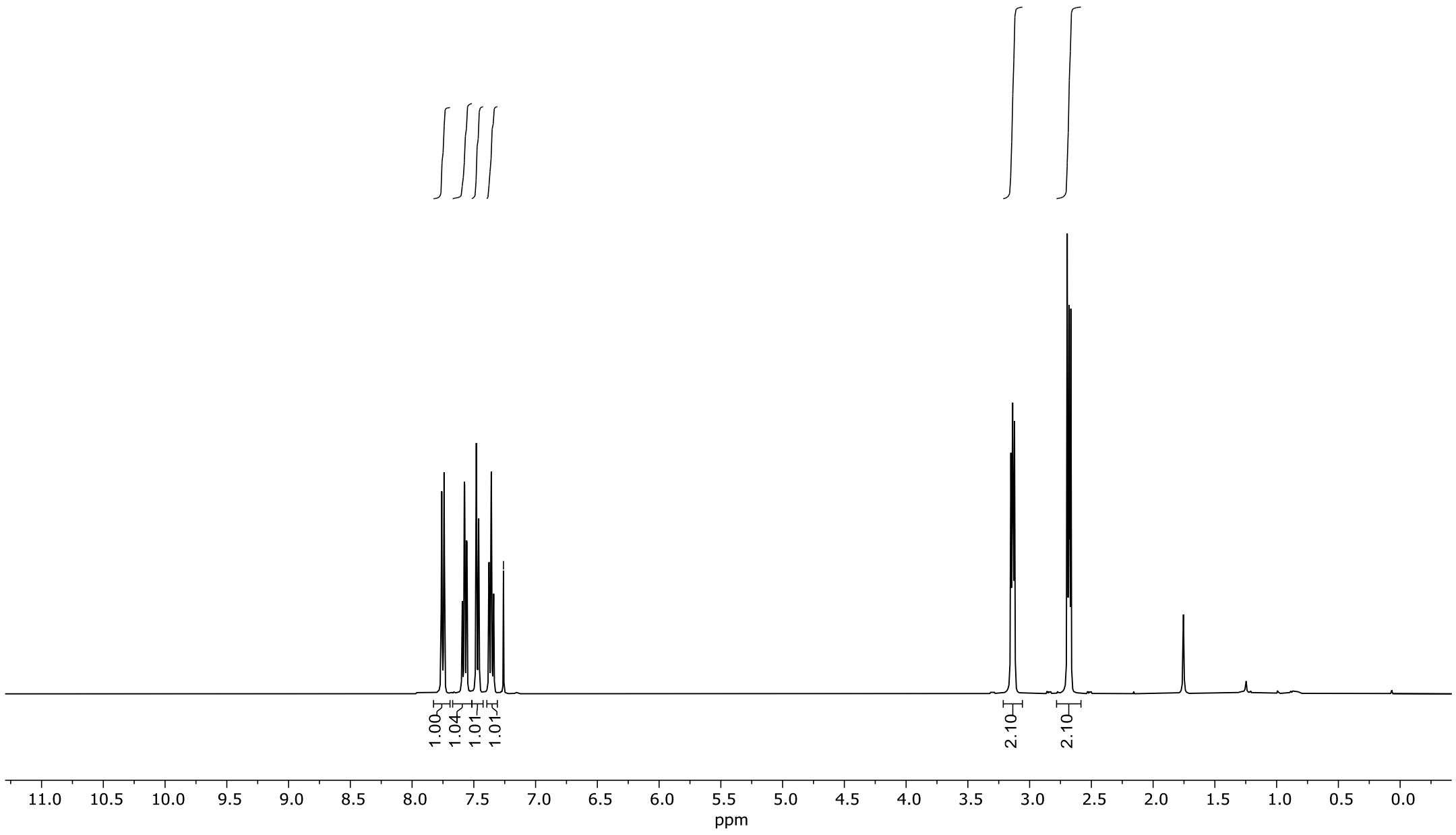

^{13}C-NMR (101MHz)

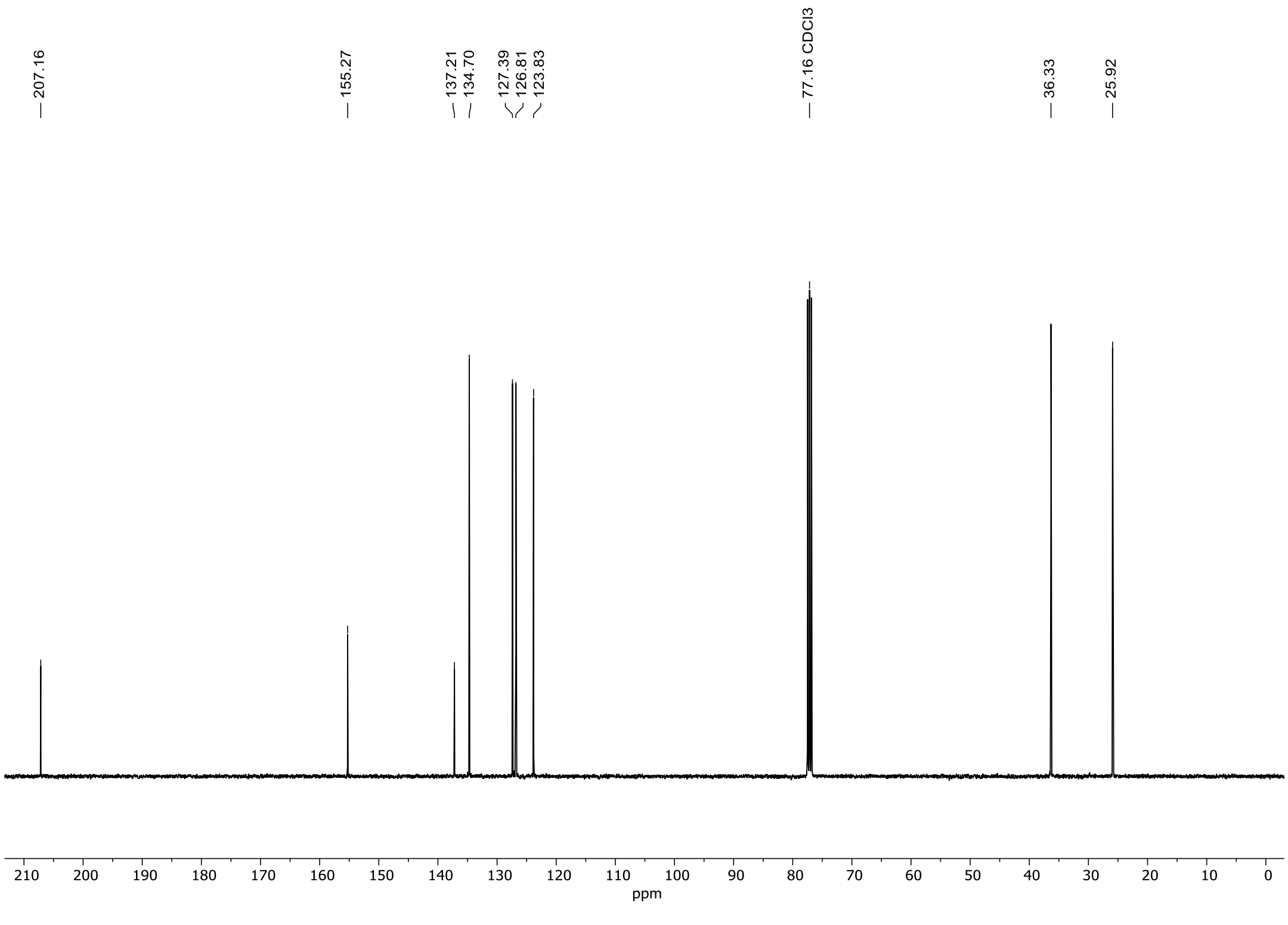

3,4-Dihydronaphthalen-1(2H)-one (18)

^{1}H-NMR (400MHz)

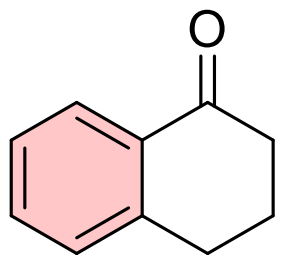

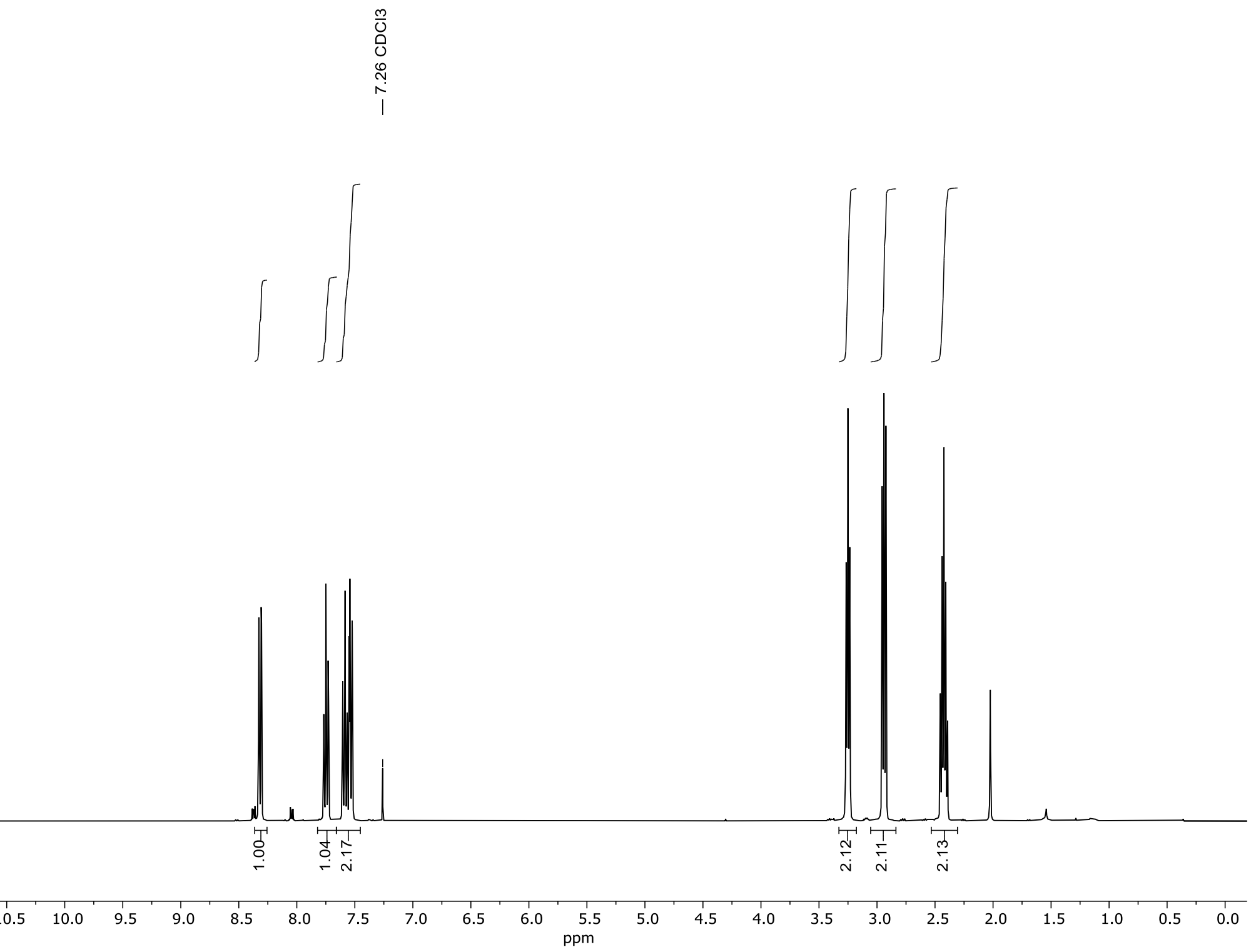

^{13}C-NMR (101MHz)

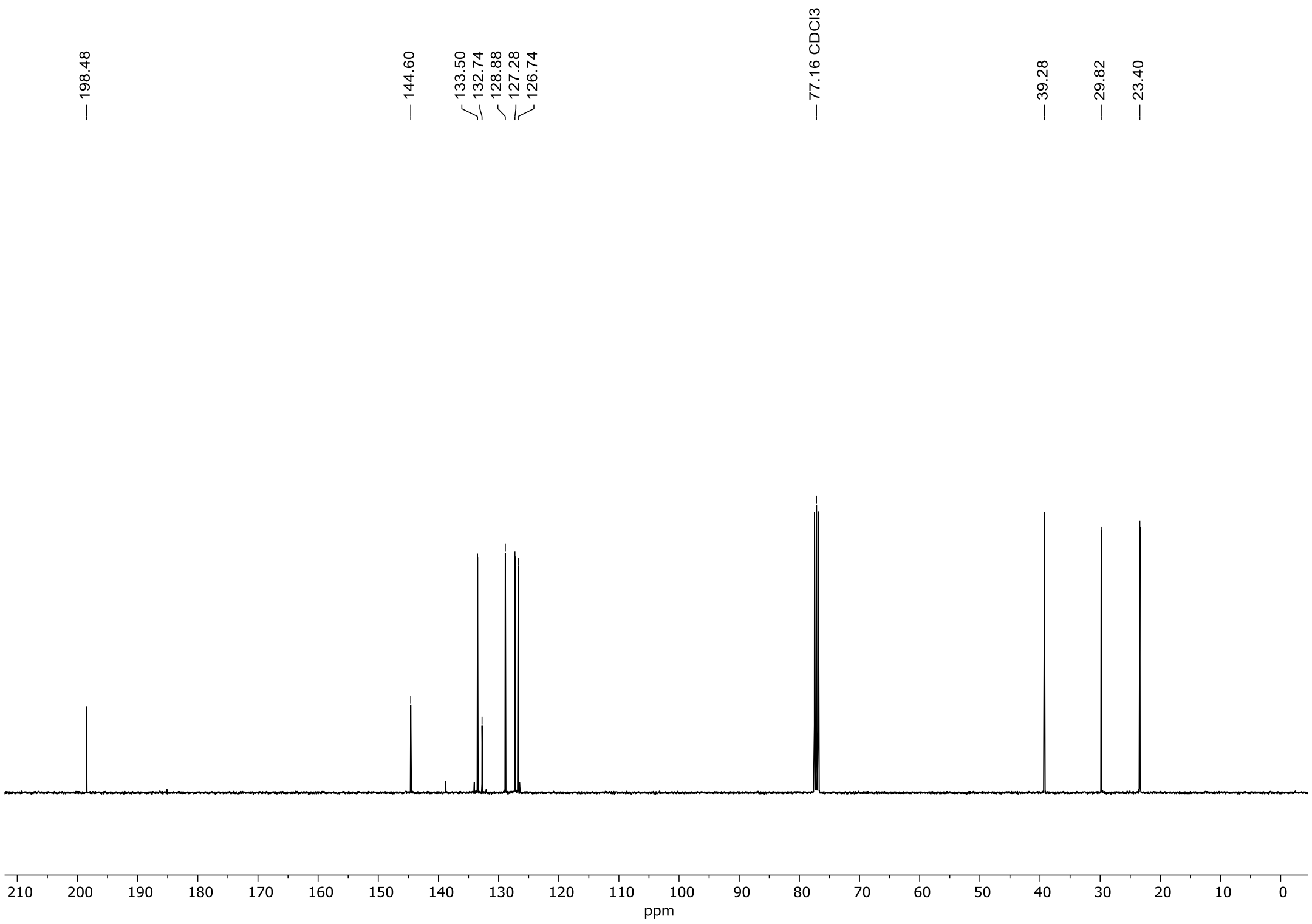

4-Methoxybenzaldehyde (19)

^{1}H-NMR (400MHz)

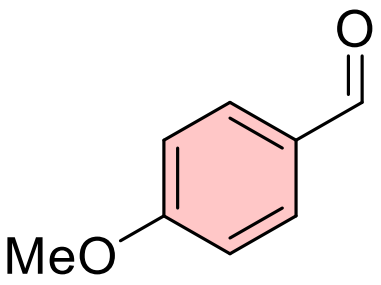

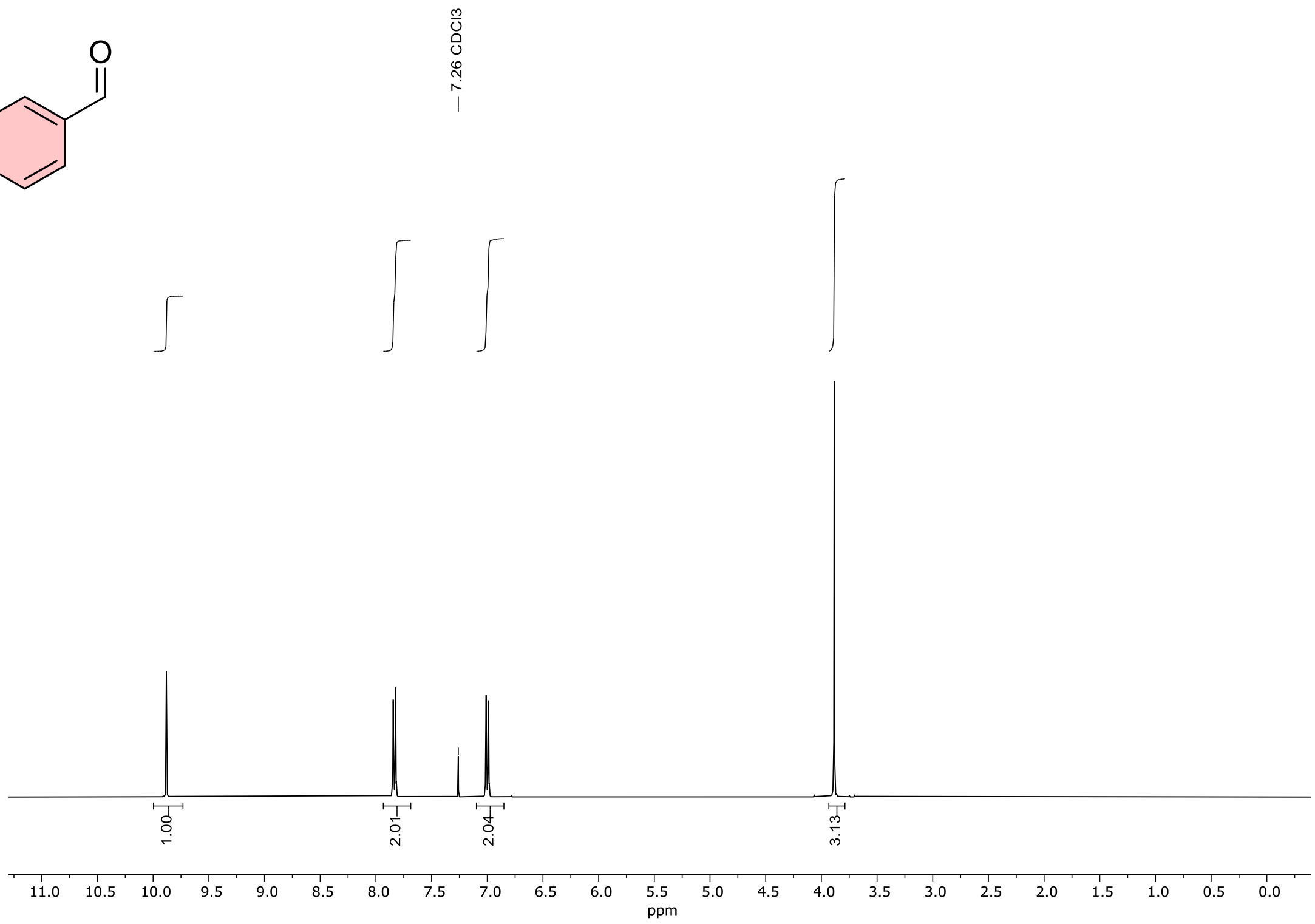

^{13}C-NMR (101MHz)

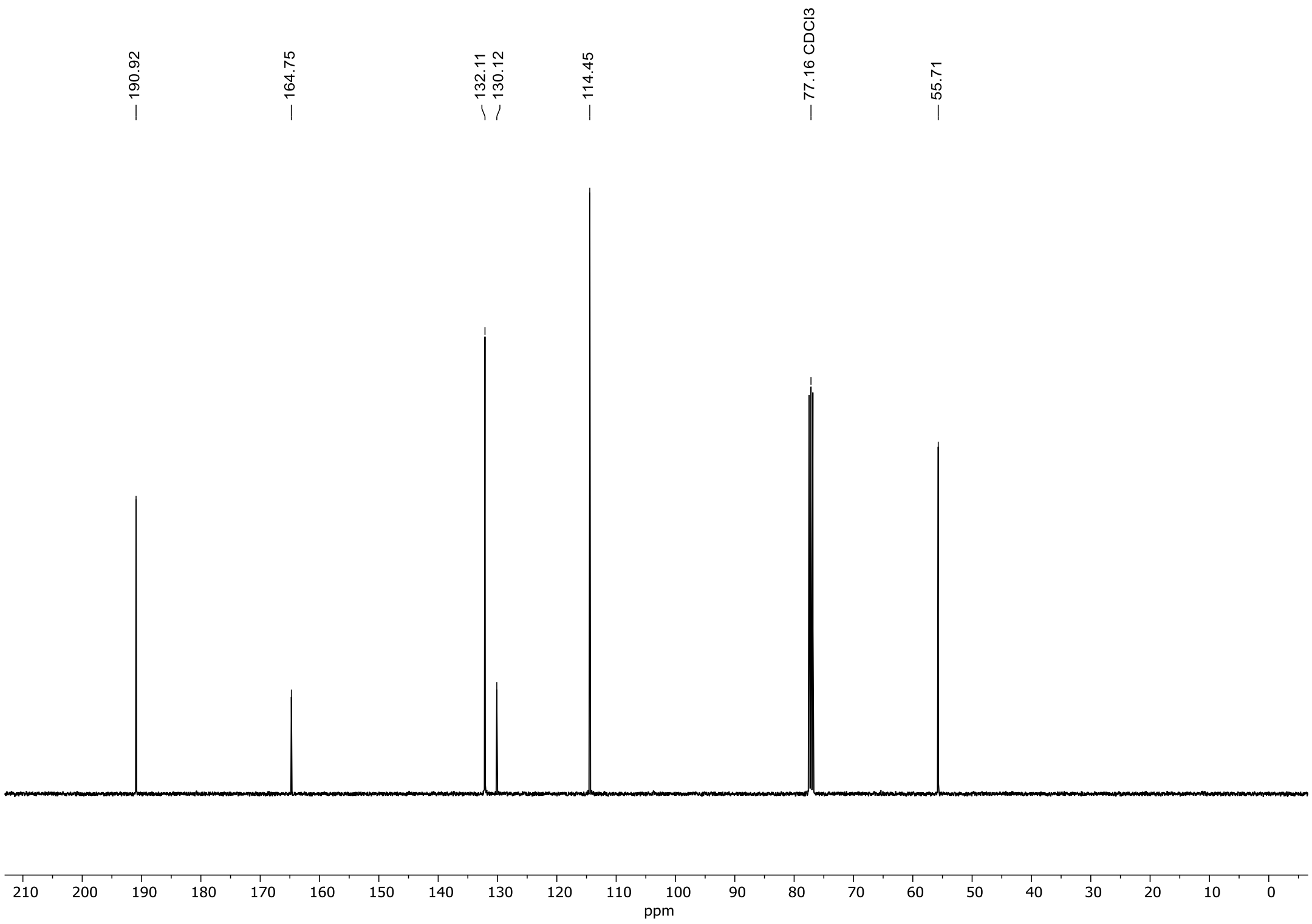

4-Methylbenzaldehyde (20)

^{1}H-NMR (400MHz)

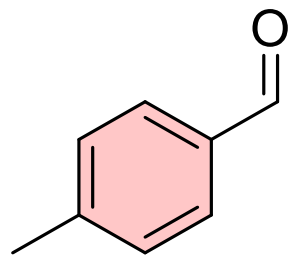

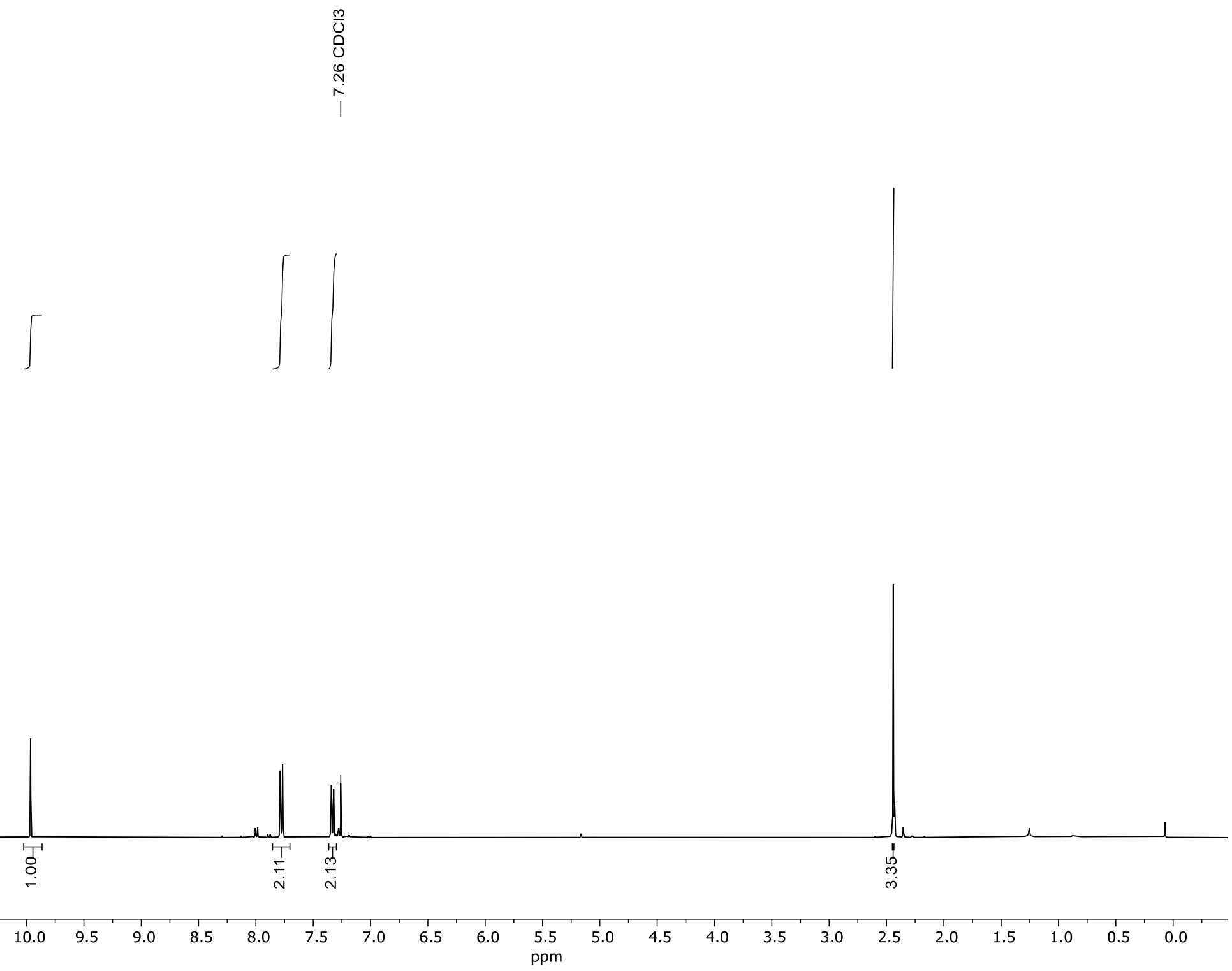

^{13}C-NMR (101MHz)

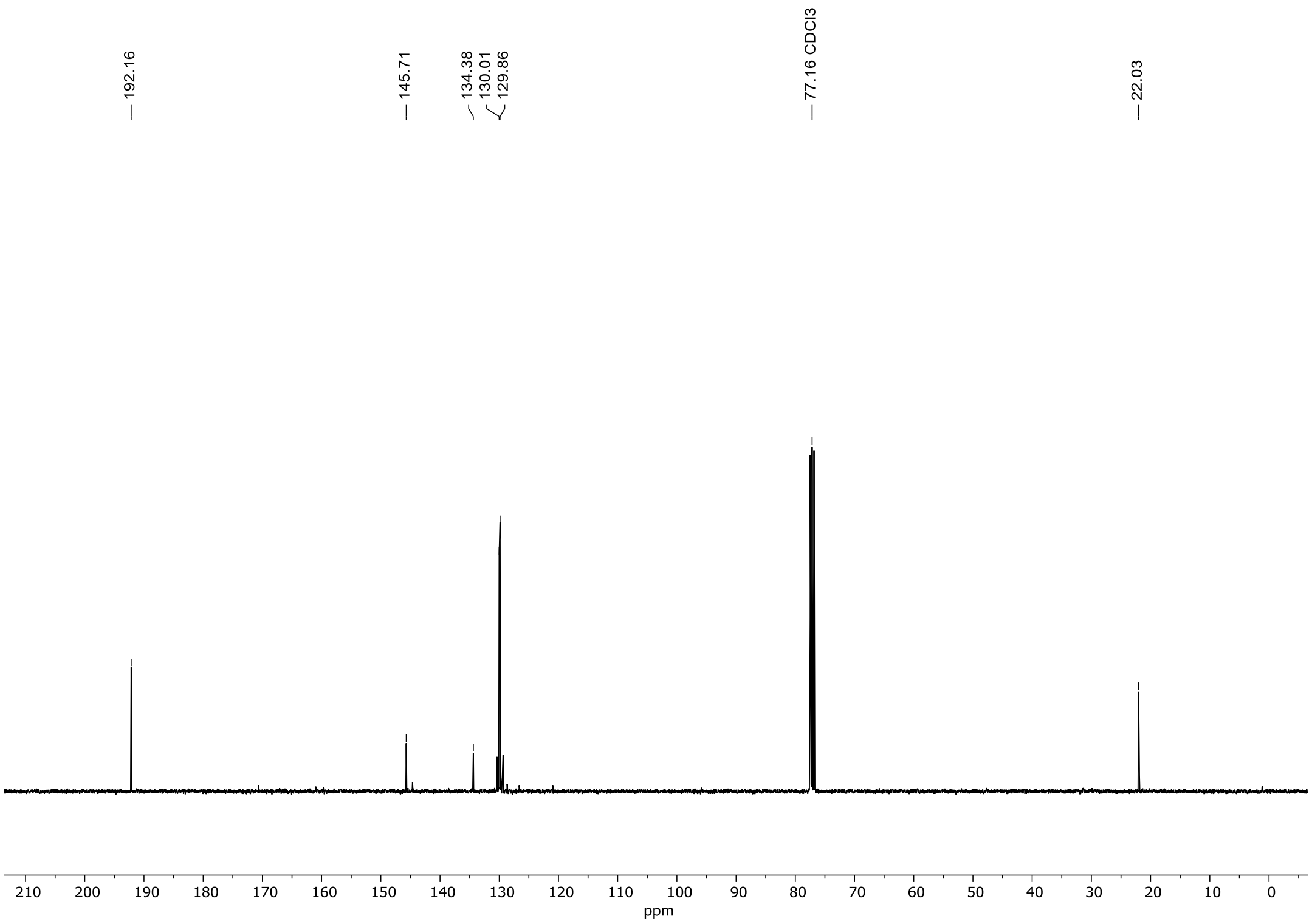

4-(*tert*-Butyl)benzaldehyde (21)

^{1}H-NMR (400MHz)

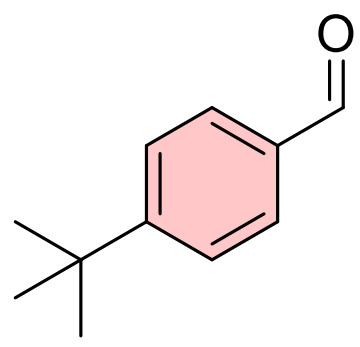

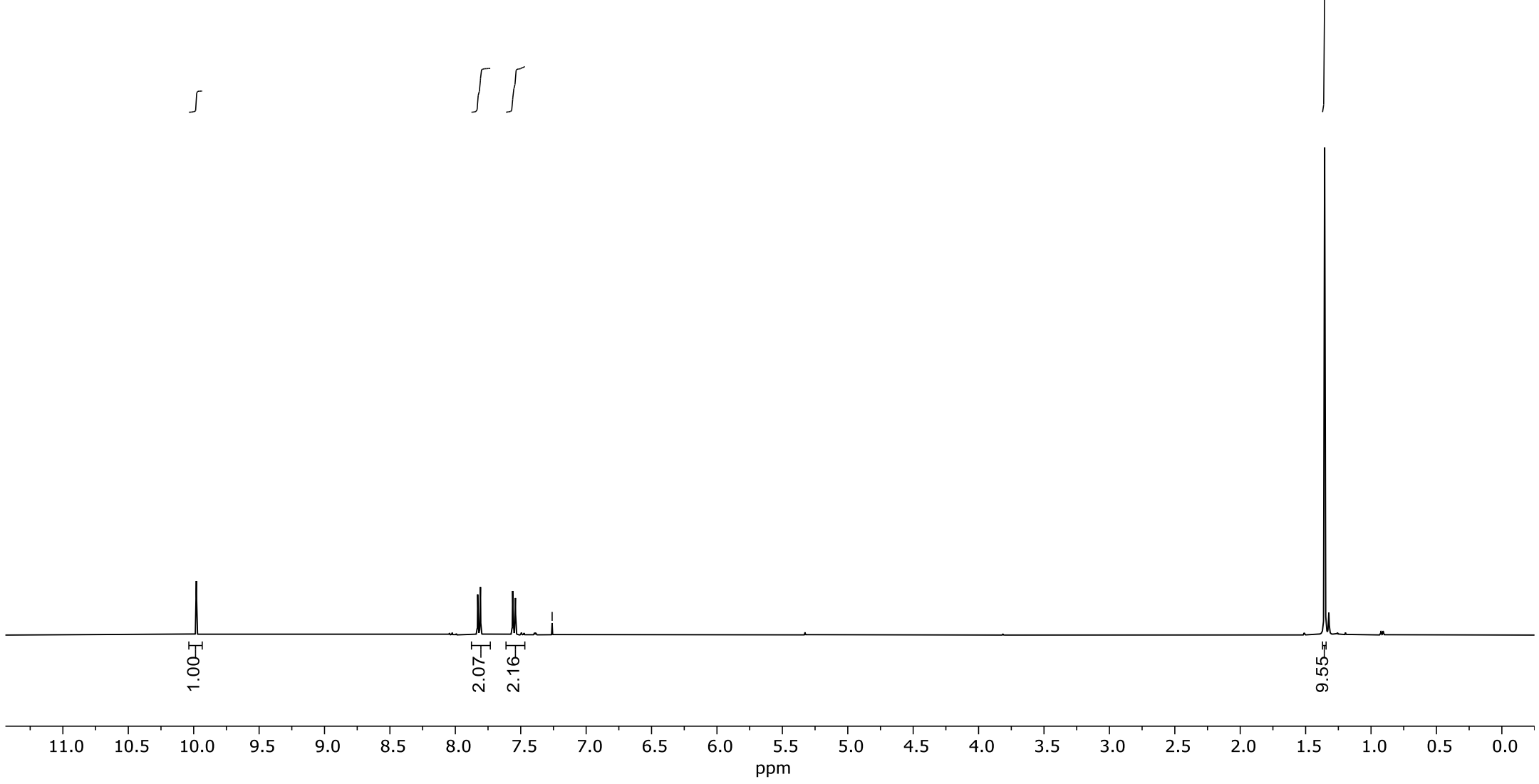

^{13}C-NMR (101MHz)

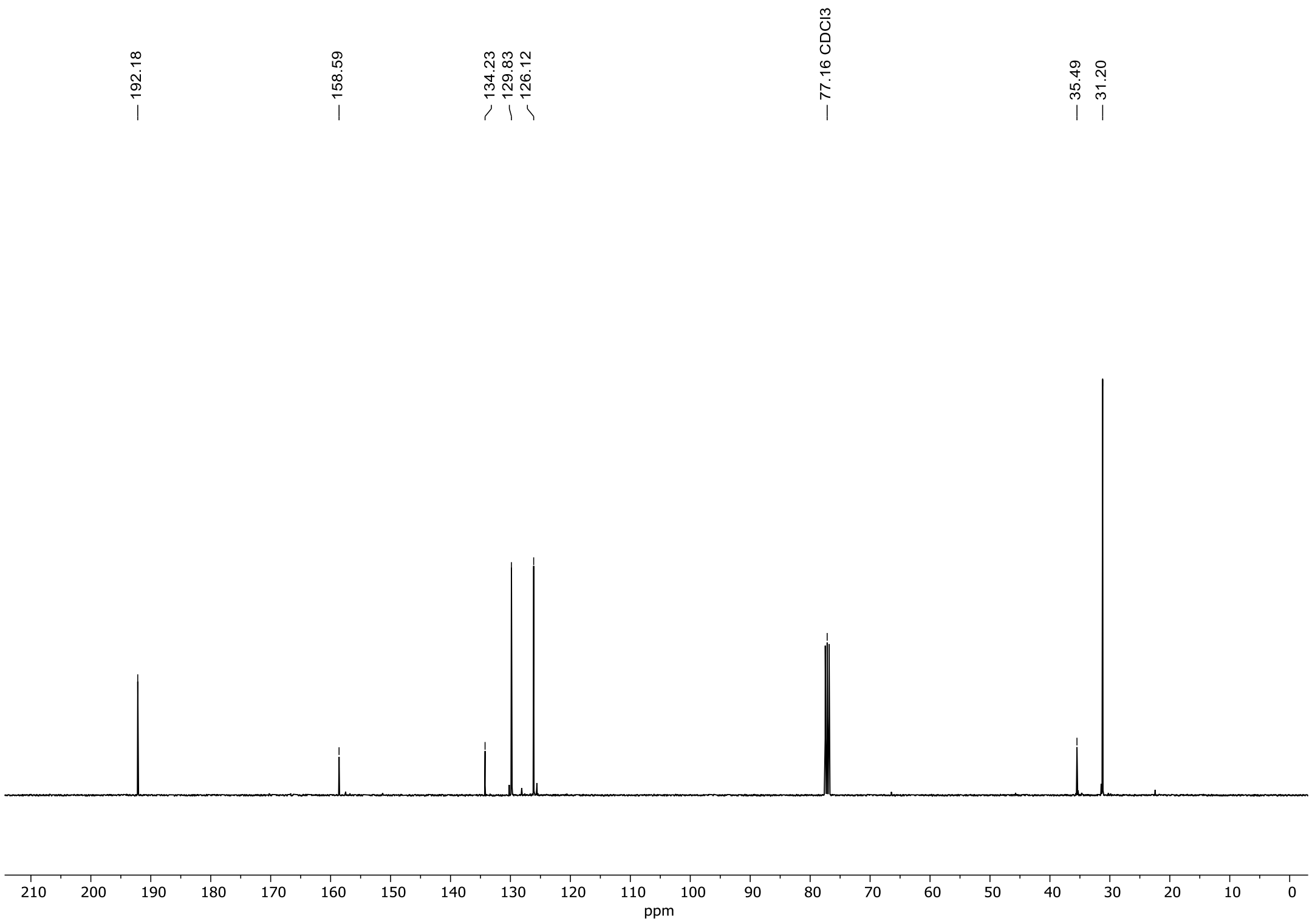

4-Bromobenzaldehyde (22)

^{1}H-NMR (400MHz)

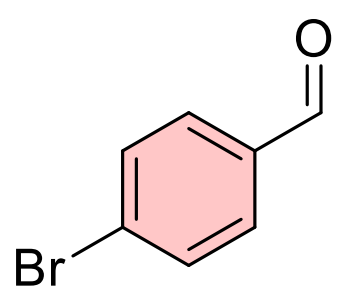

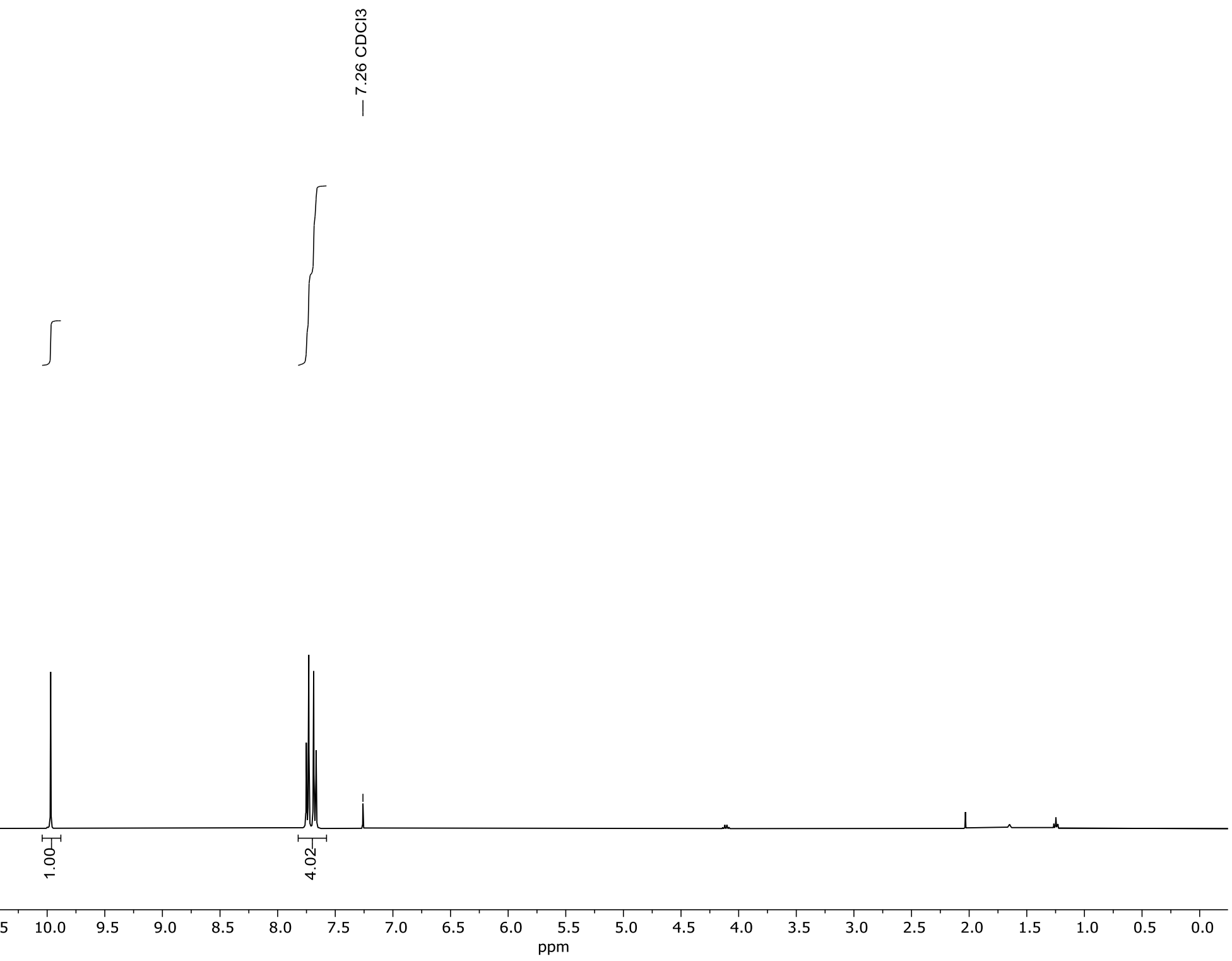

^{13}C-NMR (101MHz)

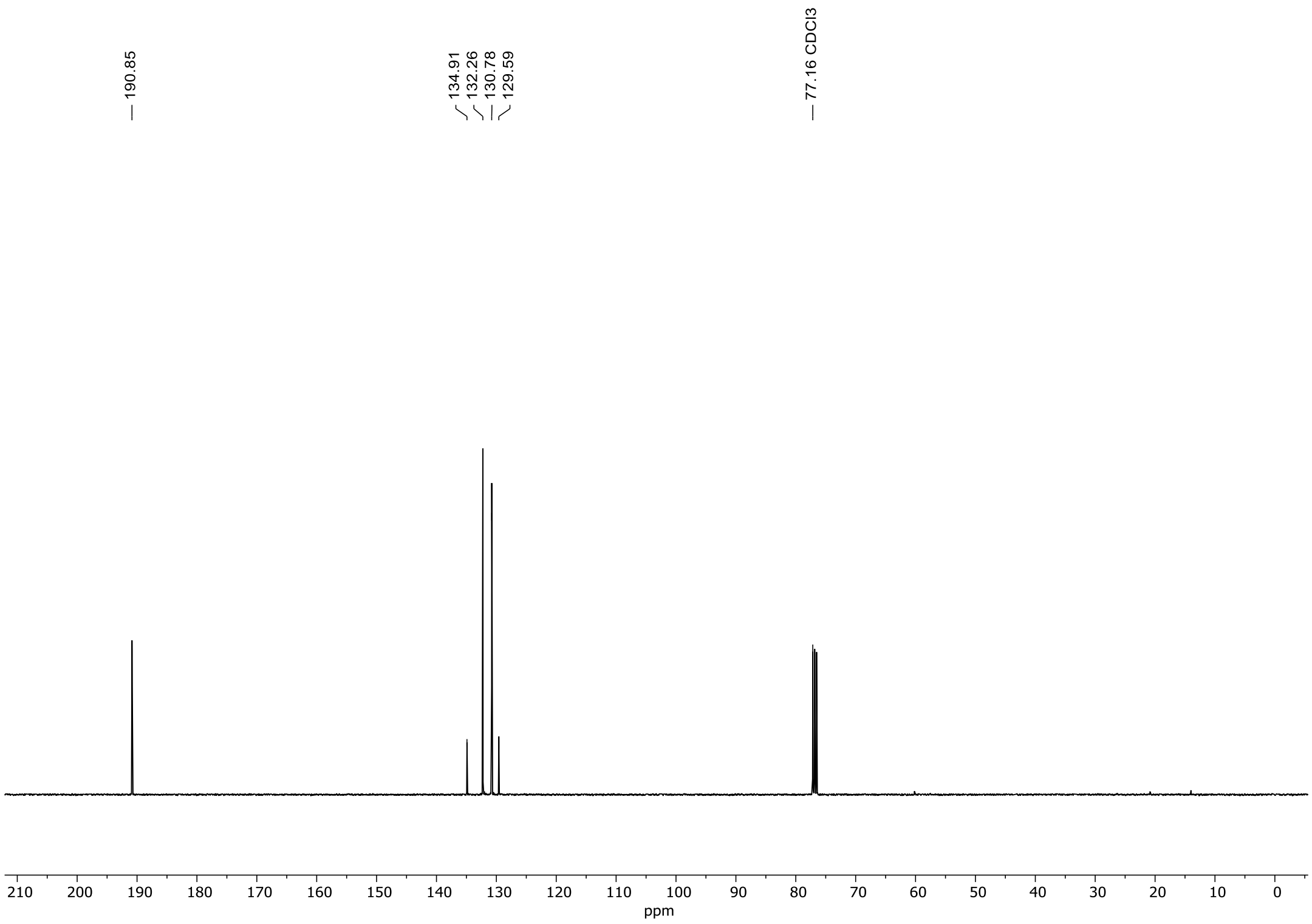

4-Chlorobenzaldehyde (23)

^{1}H-NMR (400MHz)

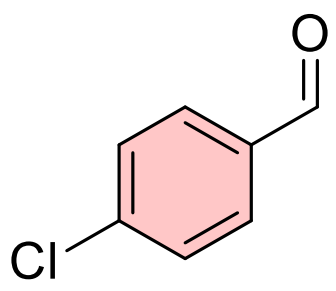

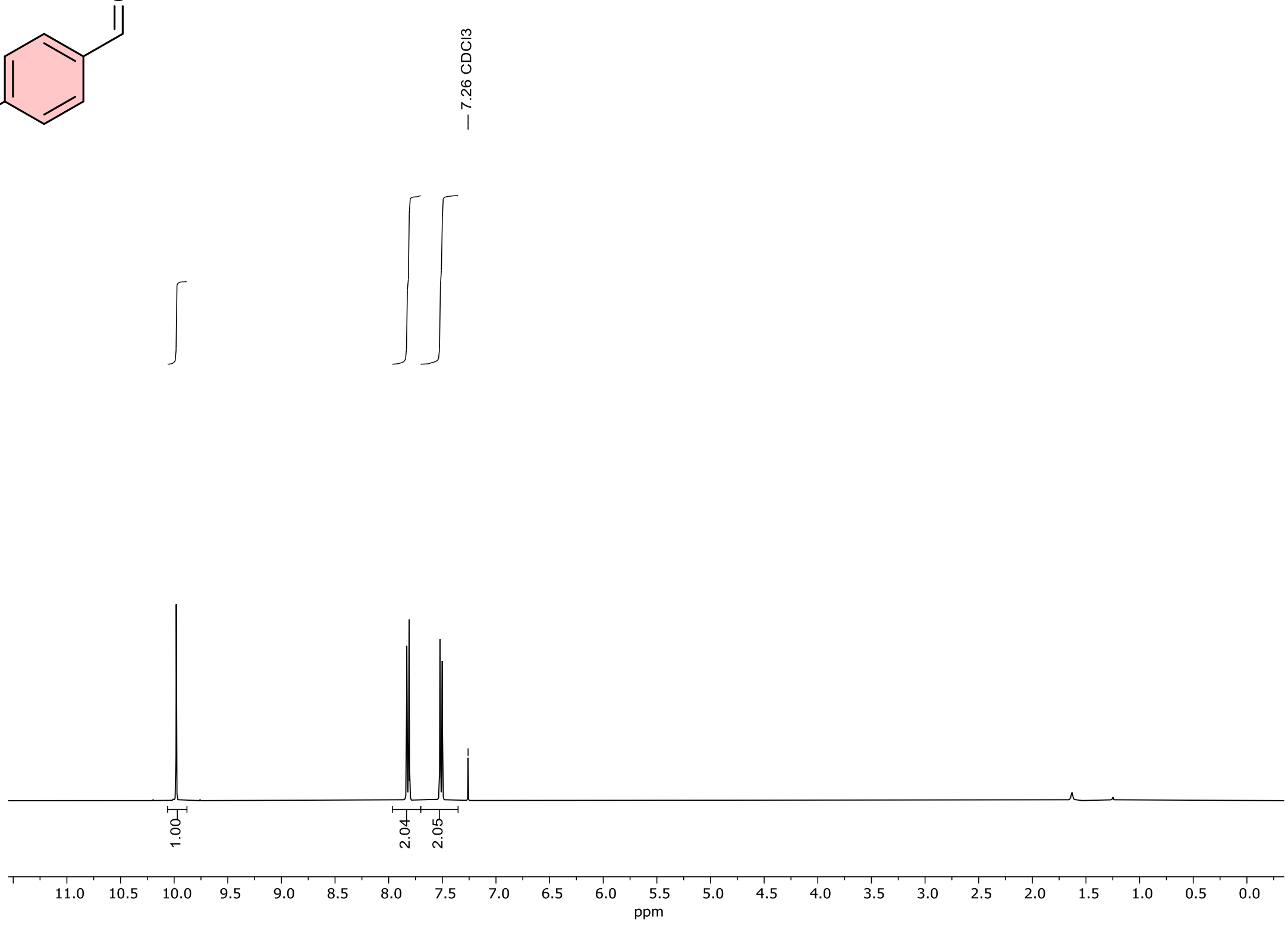

^{13}C-NMR (101MHz)

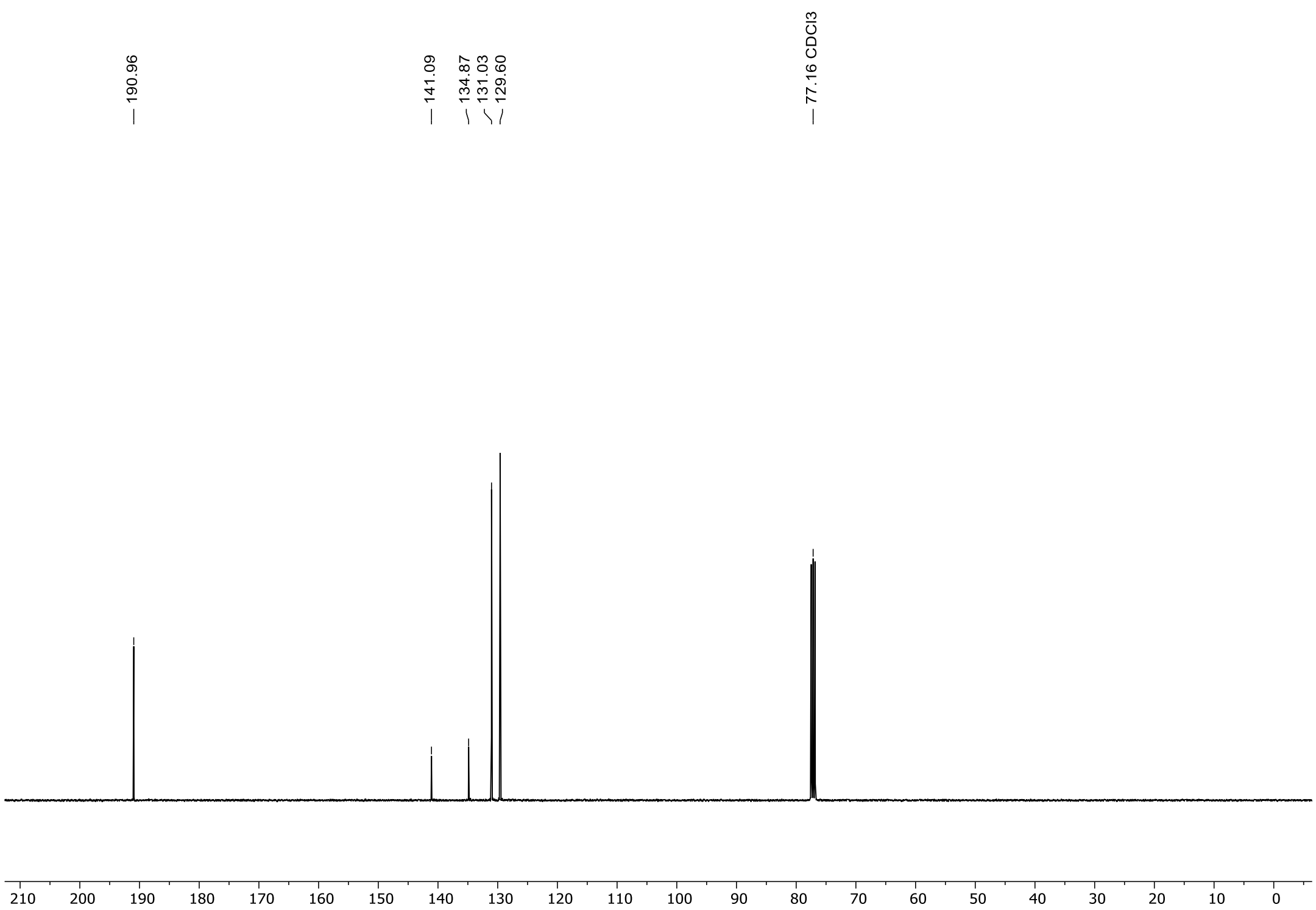

3-Methoxybenzaldehyde (24)

^{1}H-NMR (400MHz)

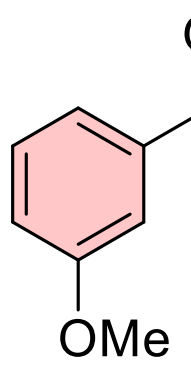

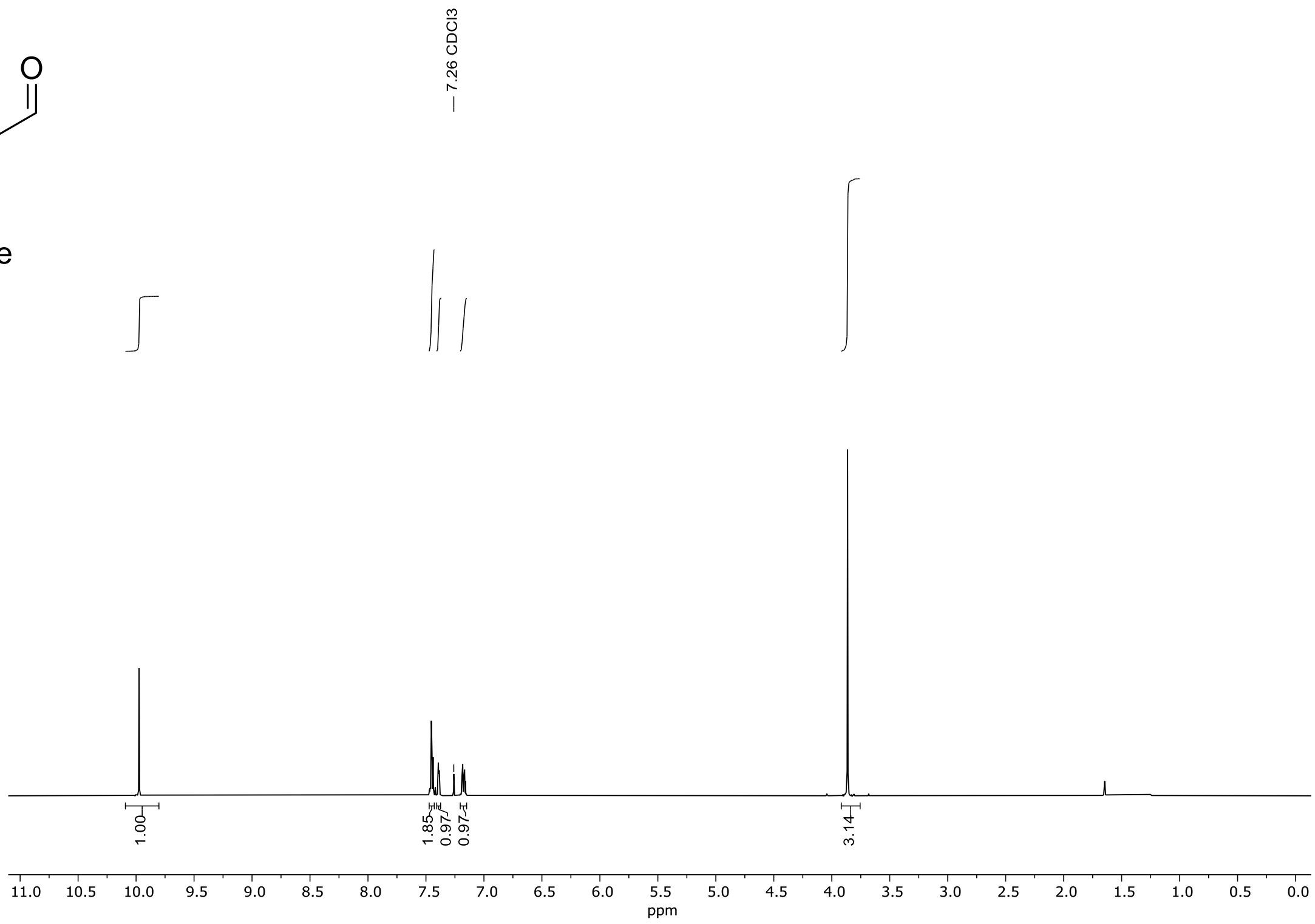

^{13}C-NMR (101MHz)

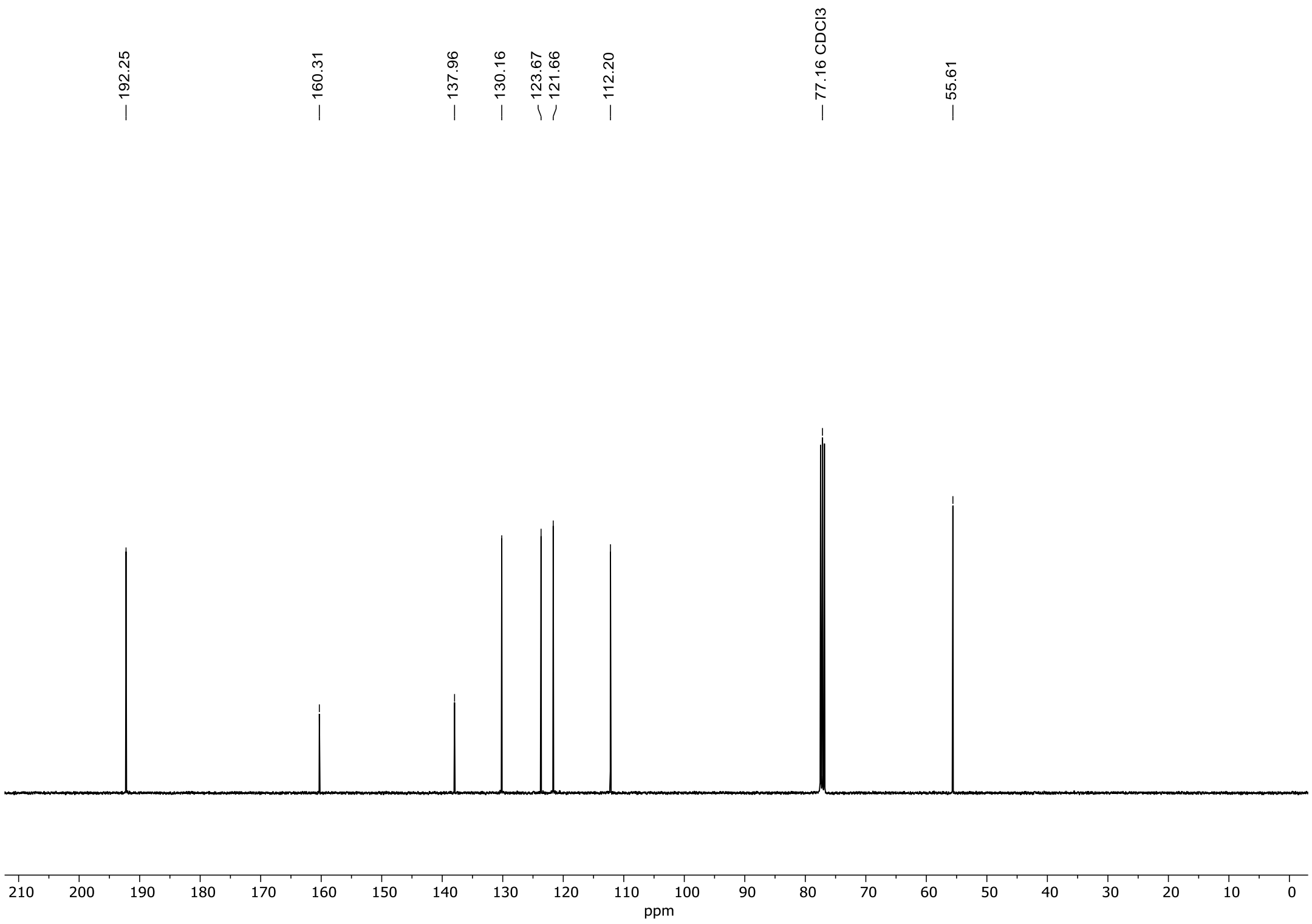

2-Methoxybenzaldehyde (25)

^{1}H-NMR (400MHz)

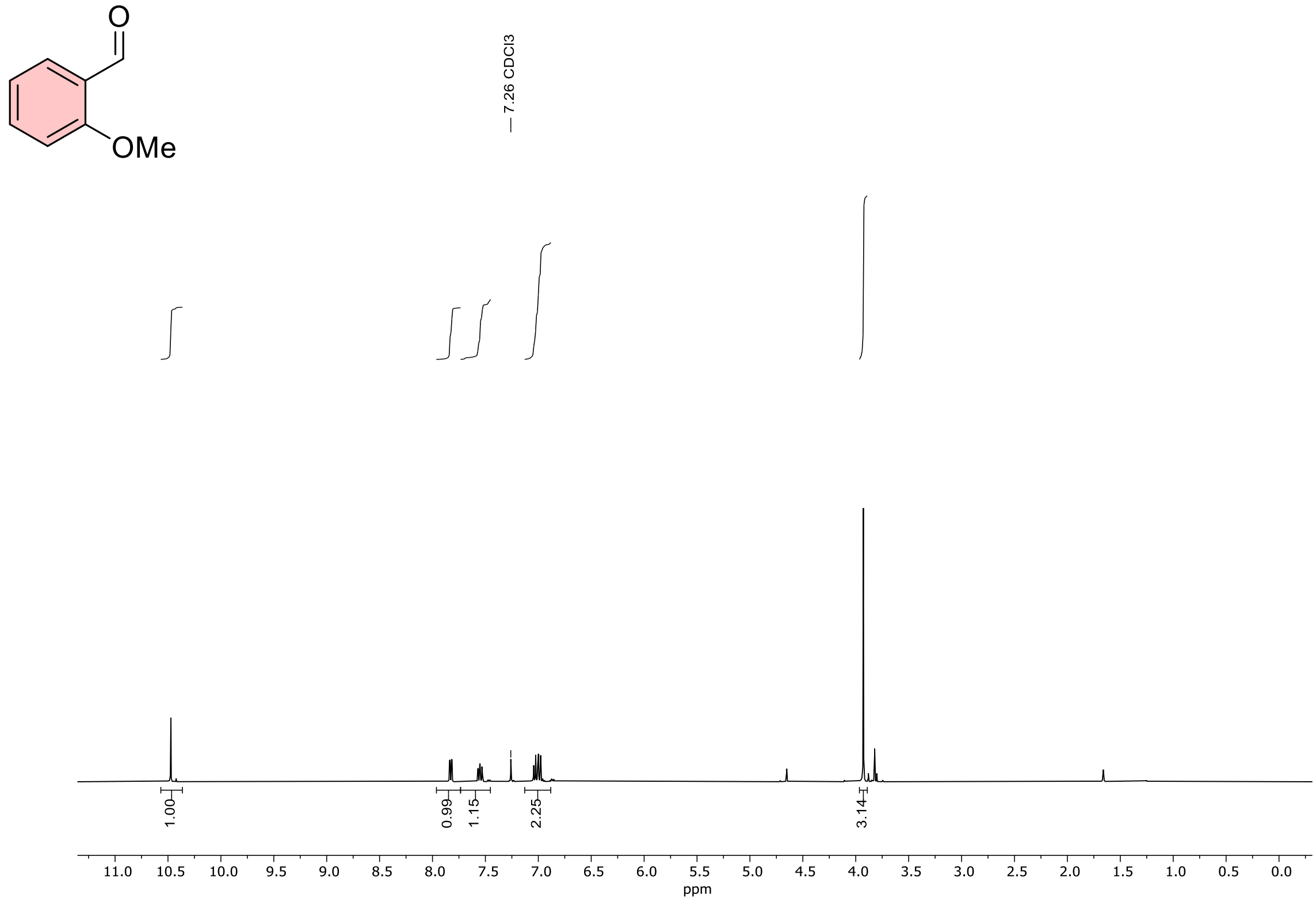

^{13}C-NMR (101MHz)

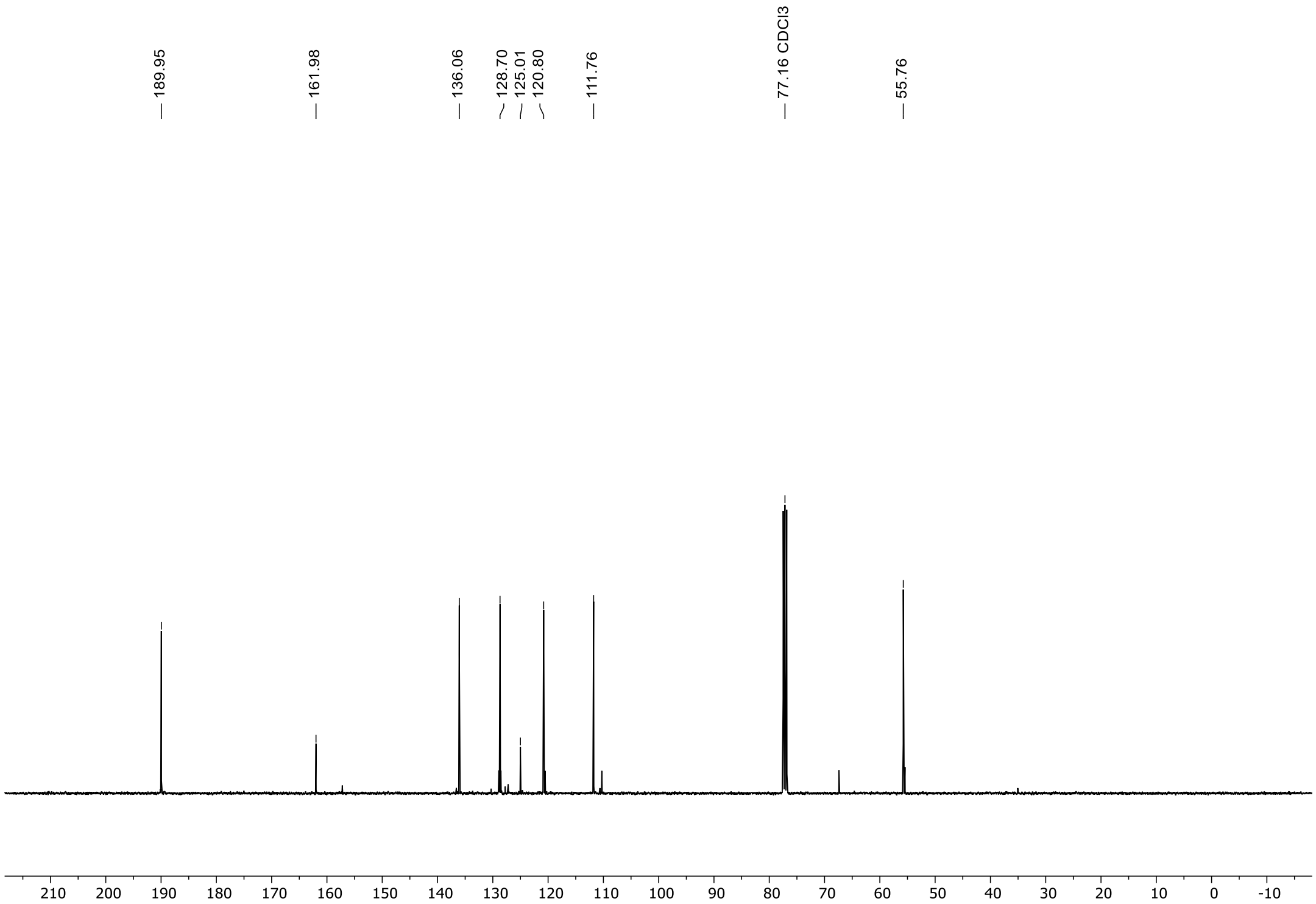

3,4,5-Trimethoxybenzaldehyde (26)

^{1}H-NMR (400MHz)

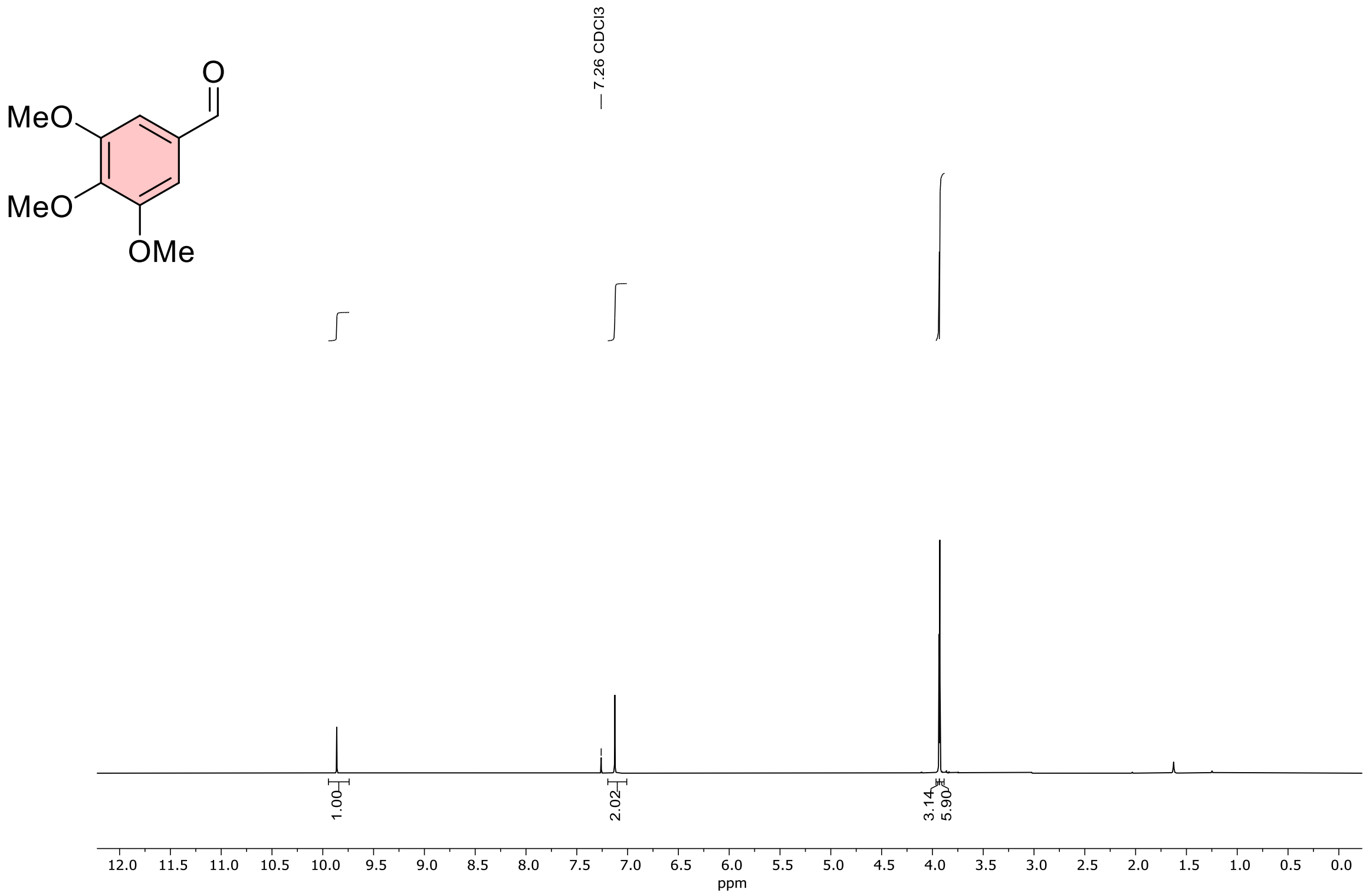

^{13}C-NMR (101MHz)

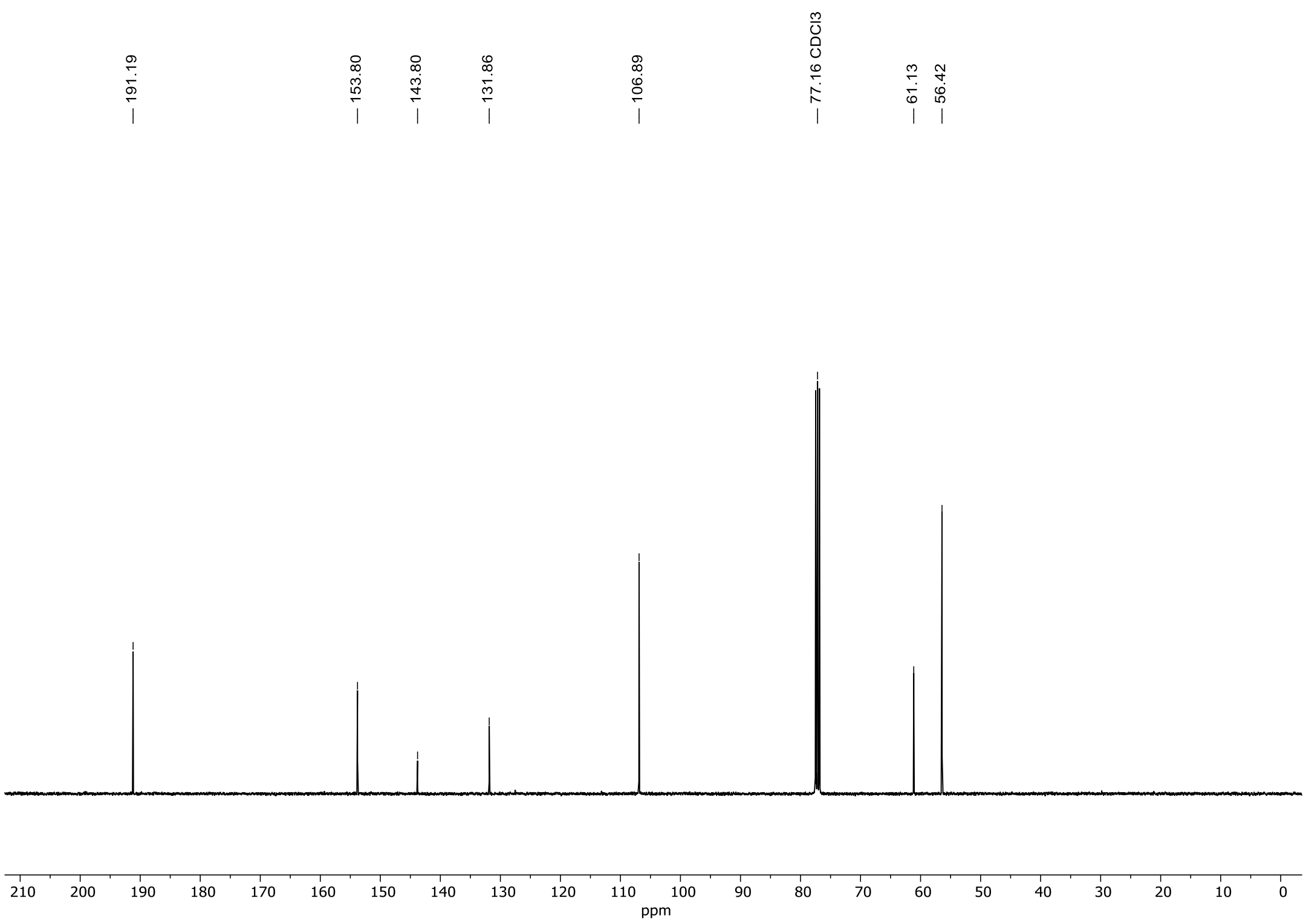

Supplement: Supplementary file 1 [file CSSC-19-e71146-s001.pdf]
